# A randomised, two-arm (1:1 ratio), double blind, placebo controlled phase III trial to assess the efficacy, safety, cost and cost-effectiveness of Rituximab in treating de novo or relapsing NS in patients with MCD/FSGS (TURING)

**DOI:** 10.1186/s12882-024-03576-0

**Published:** 2024-08-07

**Authors:** Lisa C Willcocks, Wendi Qian, Ruzaika Cader, Katrina Gatley, Hira Siddiqui, Endurance Tabebisong, Karlena Champion, Andreas Kronbichler, Liz Lightstone, David Jayne, Edward Wilson, Megan Griffith

**Affiliations:** 1 Cambridge University Hospital NHS Trust, Hills Road, Cambridge, CB2 2QQ UK; 2 Department of Internal Medicine IV, Medical University Innsbruck, Innsbruck, Austria; 3 Imperial College, London, UK; 4 Imperial College Hospital NHS Trust, London, UK; 5 University of Cambridge, Biomedical Campus, Hills Road, Cambridge, UK; 6 University of Exeter, Exeter, UK

**Keywords:** Minimal change disease, Focal segmental glomerulosclerosis, Nephrotic syndrome, Rituximab, Remission maintenance

## Abstract

**Background:**

Minimal Change Disease (MCD) and Focal Segmental Glomerulosclerosis (FSGS) are a spectrum of disease causing the nephrotic syndrome (NS), characterised by proteinuria with debilitating oedema, as well as a high risk of venous thromboembolic disease and infection. Untreated, 50–60% patients with FSGS progress to end stage kidney disease after 5 years. These diseases respond to immunosuppression with high dose glucocorticoids, but 75% will relapse as the glucocorticoids are withdrawn, leading to significant morbidity associated with prolonged use. In children, the B cell depleting monoclonal antibody rituximab reduces relapse risk, but this drug has not been tested in randomised controlled trial in adults.

**Methods:**

130–150 adults with new or relapsing MCD/FSGS, from UK Renal Units, are being randomised to receive either rituximab (two 1 g infusions two weeks apart) or placebo. Partipicipants are recruited when they present with nephrosis, and all are treated with glucocorticoids as per KDIGO guidelines. Once in remission, prednisolone is withdrawn according to a pre-specified regimen. If in remission at 6 months, participants receive a further dose of trial drug. If they relapse, they are unblinded, and if they have received placebo, they are offered open label rituximab with protocolised prednisolone as in the main phase of the trial. The primary end point is time from remission to relapse. A number of secondary endpoints will be assessed including the effect of rituximab on: (1) NHS and societal resource use and hence cost: (2) safety: (3) other measures of efficacy, such as achievement of partial and complete remission of NS and the preservation of renal function: (4) health status of participant.

**Trial Registration:**

TURING received ethical approval on 14 Jun 2019 - REC reference: 19/LO/0738. It is registered on EudraCT, with ID number: 2018-004611-50, with a start date of 2019-06-14.

**Supplementary Information:**

The online version contains supplementary material available at 10.1186/s12882-024-03576-0.

## Background

Minimal Change Disease (MCD) and Focal Segmental Glomerulosclerosis (FSGS) are auto-immune renal diseases that present with a common clinical phenotype, the nephrotic syndrome (NS), characterised by heavy proteinuria and debilitating oedema. When nephrotic, patients require frequent hospital admissions; infections and venous thrombo-embolism are common and can be fatal. With treatment, patients with MCD usually have preserved renal function, however, those with FSGS frequently still progress to end stage kidney disease (ESKD), requiring renal replacement therapy (RRT). Patients with FSGS and NS have five-year renal survival rates (without dialysis or transplantation) of 50–60% [1]. 10 year survival improves to 90–100% if remission from proteinuria is achieved [2, 3].

Primary MCD and FSGS are rare, affecting about 10/million population/year. Together they are responsible for 50% of adult idiopathic NS presentations [4]. MCD and FSGS are historically described as two separate disease entities. However, emerging data indicate that they are part of a disease continuum [5]. Both conditions are characterised by electron-microscopic evidence within the glomerulus of diffuse effacement of podocyte foot processes, resulting in a severe functional defect in selective permeability. In FSGS, there is additional focal (i.e. in some areas of the kidney cortex) and segmental (segments of the glomerulus are affected) fibrosis and occlusion of the glomerular capillaries. It is notable that, in FSGS, glomeruli not displaying segmental lesions demonstrate the classical features of MCD at electron microscopy.

### Pathogenesis

The aetiology of primary MCD and FSGS is not fully understood. However, there is evidence of a circulating factor, or factors, in MCD and FSGS [6]. Definitive evidence of a circulating factor is provided by the recurrence of NS following kidney transplantation in 20–30% of patients with FSGS [7]. This factor, or factors may be an auto-antibody: in a recent study, 29% patients with MCD had anti-nephrin antibodies in the serum at the time of nephrosis, and on renal biopsy, IgG co-localised with nephrin [8] Anti-nephrin antibodies have also been reported in patients with recurrent FSGS [9].

### Current treatment

Both diseases respond to immunosuppression with glucocorticoids in the majority of patients, with MCD typically responding faster than FSGS. Response rates may be higher in MCD at 90%, compared to those in FSGS at 40–80%. In steroid responsive patients with both diseases, recurrent relapses occur in approximately 75% when the steroid dose is reduced or withdrawn. These frequently relapsing (FR) or steroid dependent (SD) patients accrue a high steroid exposure over time, with weight gain, diabetes, infection and osteoporosis. In patients with FSGS, kidney function deteriorates if long term remission of proteinuria cannot be achieved. In one retrospective analysis (*n* = 197), 23% (*n* = 45) progressed to dialysis after a median follow-up of 1.8 years from the time of kidney biopsy diagnosis [9]. Achieving remission is very important for avoiding ESKD– in a cohort study of 281 patients with nephrotic FSGS, achieving at least partial remission was associated with markedly improved kidneyl survival with a hazard ratio of 0.48 (95% confidence interval 0.24–0.96:*p* = 0.04) [10]. ESKD is associated with poor quality of life and a risk of death exceeding most cancers [11] and is expensive for the NHS [12].

Due to the morbidity of prolonged/repeated course of steroids, steroid sparing agents are regularly used in MCD/FSGS. Cyclophosphamide is thought to be most effective at maintaining remission in SD/FR MCD/FSGS [13], but the severe cumulative toxicity precludes long term use. There is also evidence that calcineurin inhibitors (CNIs) are effective at maintaining remission. In an open-label prospective cohort study of 26 adults with FR or SD NS, tacrolimus was as effective as cyclophosphamide at inducing and maintaining remission at 2 years [14]. An RCT in patients with MCD showed tacrolimus was as effective as high dose prednisolone in inducing remission [15]. However, relapse rates are high on stopping therapy, so repeated prolonged courses are often required for many years. CNIs themselves are nephrotoxic, and patients require long term follow up with close monitoring of levels in the clinic for the duration of their therapy.

### Rituximab

Rituximab is a monoclonal antibody that targets and depletes B cells via CD20. It is a licensed treatment for other auto-immune diseases, including rheumatoid arthritis and antineutrophil cytoplasmic antibody (ANCA) associated vasculitis, where it has an excellent safety profile. In a pooled analysis of 3,194 patients with rheumatoid arthritis exposed to 11,962 patient years of rituximab [16], serious infections occurred at a rate of 3.94 per 100 patient years (comparable to both methotrexate and placebo). Importantly, the risk of serious infections was stable over time, even with multiple courses of treatment. Opportunistic infections were very rare, and only two cases of pulmonary tuberculosis occurred. Rates of viral infections, including herpes zoster reactivation (9/1000 patient years), were low. Rituximab may therefore be safer than existing treatments for MCD/FSGS.

Randomised trial evidence to support the use of rituximab in MCD/FSGS is limited to paediatric studies (Table 1). Here, NS is almost always due to MCD, and kidney biopsy is rarely done. Instead, patients are treated on the basis of their clinical presentation with steroid-sensitive (SS), SD or FR NS. Ravani et al. randomised 54 children with SDNS in an open-label non-inferiority design to receive rituximab and reduced dose steroid and cyclosporine, or standard steroid and cyclosporine doses [17]. Proteinuria was lower after 3 months in the rituximab arm, and relapses occurred in only 18.5% of the rituximab arm compared with 48% in the control arm (*p* = 0.029). Notably, the probability of being drug free at 3 months were 62.9% and 3.7%, respectively (*p* < 0.001). In a double-blind randomised trial, Iijima and colleagues randomly assigned 48 children with SDNS to rituximab or placebo. Patients in the rituximab arm demonstrated a 50% increase in the median time to relapse (*p* = 0.0001) [18]. In a further non-inferiority open-label randomised trial [19], Ravani et al. assigned 30 children with SDNS to receive either continued steroid therapy or a single IV infusion of rituximab. After 3 months, proteinuria was 42% lower with rituximab than control. The median time to relapse with rituximab was 18 months, whereas 14/15 control patients relapsed within 6 months.

Evidence to support the use of rituximab in adults with MCD/FSGS is more limited (Table 1). There are no RCTs, but a recent meta-analysis of 16 observational studies included a total of 221 patients (23.1% FSGS, 76.9% MCD) [20]. Mean follow-up duration was 26.3 ± 12.8 months. From the analysis of five studies with FSGS patients (*n* = 51), the overall remission rate and relapse rate of RTX therapy was 53.6% (95% CI, 15.8–87.6%) and 47.3% (95% CI, 25.4–70.2%), respectively. Complete remission occurred in 42.9%. In contrast, from the analysis of 11 studies with MCD patients (*n* = 170), the overall remission rate and relapse rate of RTX therapy was 80.3% (95% CI, 68.5–88.5%) and 35.9% (95% CI, 25.1–48.4), respectively. Complete remission occurred in 74.7%. Incidence of serious adverse events was 0.092 events/year. However, these studies comprised a variety of rituximab regimes, with dosing varying in both frequency and amount, and glucocorticoid dose was also variable. Time spent in the remission state was not given.

**Table 1:** Summary of evidence to support use of rituximab in MCD/FSGS in children and adults

Evidence to support use of rituximab in paediatric steroid-dependent nephrotic syndrome (SDNS)		
Research article	Description	Results
Ravani et al., 2011 [17] *Short-term effects of rituximab in children with steroid- and calcineurin-dependent nephrotic syndrome: RCT*	54 children with SDNS in an open-label non-inferiority design to receive rituximab and reduced dose steroid and cyclosporine, or standard steroid and cyclosporine doses	• Reduction in proteinuria in 3 months • Relapses 18.5% of rituximab vs. 48% of control arm (*p* = 0.029) • Probability of being drug free at 3 months 62.9% (*p* < 0.001)
Iijima K et al., 2014 [18] *Rituximab for childhood-onset, complicated, frequently relapsing nephrotic syndrome or steroid-dependent nephrotic syndrome: a multicentre, double-blind, randomised, placebo-controlled trial*	48 children with SDNS to rituximab or placebo	• 50% increase in the median time to relapse (p-0.0001).
Ravani et al., 2011 [19] *Rituximab in Children with Steroid-Dependent Nephrotic Syndrome: A Multicenter, Open-Label, Noninferiority, Randomized Controlled Trial*	Steroid therapy vs. a single IV infusion of rituximab	• 42% lower proteinuria with rituximab • Median time to relapse 18 months • Control arm– 14/15 relapsed within 6 months
Evidence to support use of rituximab in MCD/FSGS (adult studies)		
Hansrivijit P et al., 2020 [20] *Rituximab therapy for focal segmental glomerulosclerosis and minimal change disease in adults: a systematic review and meta-analysis*	Meta-analysis of 16 observational studies	Treatment with rituximab: Analysis of 5 studies with FSGS: *n* = 51 • Remission 53.6% (95% CI, 15.8–87.6%) • Relapse rate 47.3% (95% CI, 25.4–70.2%) • Complete remission 42.9% Analysis of 11 studies with MCD: *n* = 170 • Remission − 80.3% (95% CI, 68.5–88.5%) • Relapse − 35.9% (95% CI, 25.1–48.4) • Complete remission 74.7% Serious Adverse Events- 0.092/ year

## Methods: participants, interventions, and outcomes

### Design

TURING is a randomised, two-arm (1:1 ratio), double blind, placebo controlled phase III trial to assess the efficacy, safety, cost and cost-effectiveness of rituximab in treating de novo or relapsing NS in patients with MCD/FSGS.

In addition to the main phase of the trial, the participating Renal Units also offer the Open Label Phase (OLP) to the participants in the placebo arm who relapse after achievement of partial or complete remission.

### Participants

130–150 patients are being recruited from 23 Renal Units at hospitals across the UK.

#### Inclusion criteria

To be included in the trial the participant must:

- Have given written informed consent to participate
- Age 16 years or older at the time of consent
- Have confirmed NS at Day 0 (Start of Protocolised Prednisolone regimen (SPPR)) (defined as serum albumin < 35 g/l and PCR > 300 mg/mmol) secondary to MCD/FSGS with,

O De novo disease, or;

O Relapsing disease in a patient previously steroid or CNI responsive (this is defined as patients who have achieved complete or partial remission from their NS in response to treatment with steroid or CNI).

- Biopsy proven MCD or FSGS (from medical records)
- Agreed to be enrolled in the UK National Registry of Rare Renal Diseases (RaDaR)

#### Exclusion criteria

- MCD or FSGS due to secondary causes, including obesity-driven hyperfiltration, remnant kidneys, malignancy of a type likely to be associated with MCD /FSGS and genetic polymorphisms known to be associated with nephrosis.
- MCD/FSGS secondary to malignancy, including lymphoproliferative disorders
- Family history of MCD or FSGS in a first degree relative unless previously shown to be steroid responsive
- Previous rituximab within 18 months preceding Day 0 (SPPR), or 12 months if there is evidence of B cell return in peripheral lymphocyte subsets
- Previous cyclophosphamide within 6 months preceding Day 0 (SPPR)
- Prednisolone daily dose equal to or greater than 60 mg, with a course length of greater than 4 weeks, immediately prior to randomisation
- Evidence of current or past infection with Hepatitis B, C or HIV (unless appropriate prophylaxis is given and no replicating virus is detected)
- Positive serum pregnancy test (within 14 days prior to treatment with IMP in main trial and rituximab in OLP)
- Evidence of active severe infection
- Severe heart failure or severe, uncontrolled cardiac disease
- Pregnant or breast-feeding women
- Live vaccine administration in the four weeks prior to enrolment and while remaining on IMP treatment
- Previous/known hypersensitivity to prednisolone or IMP or to murine proteins (and any excipients as described in the SmPC)
- Co-enrolment in another clinical trial of an investigational medicinal product
- Any other reason which, in the opinion of the Principal Investigator (PI), renders the patient unsuitable for the trial

### Interventions

#### Rituximab

Patients receive the first dose of IMP (rituximab 1 g or placebo) within 4 weeks of starting high dose prednisolone for the treatment of their nephrotic syndrome (SPPR). They receive a second dose two weeks later, and a third dose at 26 weeks (+/- 2 weeks) if still in remission. Biosimilar rituximab is permitted but patients receive the same biosimilar for all doses.

#### Prednisolone

All patients initially receive prednisolone at a starting dose of 1 mg/kg (max 60 mg daily) to treat their nephrotic syndrome. Patients may start this prednisolone treatment prior to enrolment and randomisation. Once remission from the nephrotic syndrome is achieved, patients withdraw prednisolone according to either Regimen 1 (standard dose) or 2 (reduced dose), as pre-specified by the Primary Investigator at the time of randomisation (Table 2). Patients are stratified according to prednisolone regimen at randomisation.

**Table 2:** Dosing regimens for prednisolone once remission is achieved

Regimen 1	Regimen 2
40 mg daily for 2 weeks 30 mg daily for 2 weeks 25 mg daily for 2 weeks 20 mg daily for 2 weeks 15 mg daily for 2 weeks 10 mg daily for 2 weeks	30 mg daily for 2 weeks 20 mg daily for 2 weeks 15 mg daily for 2 weeks 10 mg daily for 2 weeks

* Thereafter, reduction will be 5 mg daily for 2 weeks, 5 mg alternate days for 2 weeks then stop or reduction to a maintenance dose of < 10 mg daily for patients on long term prednisolone (with further reduction determined by the local investigator as clinically appropriate). Variations in prednisolone doses of up to one third of the selected regime will be permitted and will not be considered protocol violations

#### Other immunosuppression

All immunosuppressants are withdrawn at entry into the trial, with the exception of calcineurin inhibitors (CNIs), which are gradually reduced and stopped, with a 25% dose reduction within 48 h of randomisation, then a 50% dose reduction (from dose at randomisation) at week 4 (+/-2 weeks) (from Day 0 SPPR), followed by a 75% dose reduction (from dose at randomisation) at week 8 (+/-2 weeks) from Day 0 SPPR). The CNI will then be stopped at week 12 (+/-2 weeks) from Day 0 SPPR. Variations in the daily CNI dose of up to 25% at each of these time points will be permitted and will not be considered protocol violations.

### Participant timeline

There are two phases to the main trial– *pre-remission*, when patients are treated with prednisolone 1 mg/kg daily (max 60 mg), followed by the *post remission* phase, when prednisolone is weaned according to Regimen 1 or 2. If the patient is in remission at 26 weeks after starting high dose prednisolone (SPPR), they receive a third dose of IMP.

**Fig. 1:**
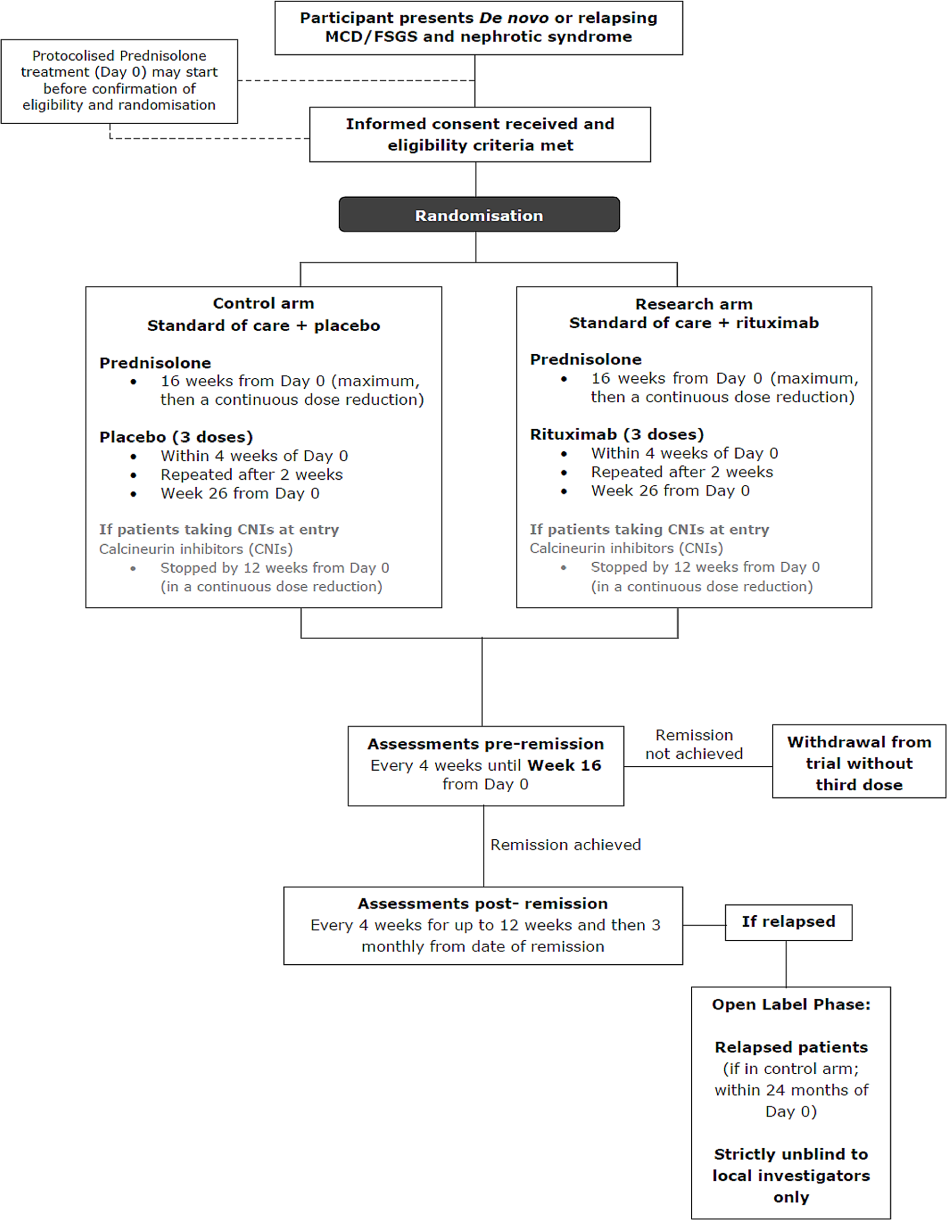
Trial schematic

If patients relapse in the trial, they may be unblinded, and may enter the OLP if they have received placebo (Fig. 1).

Participants will have a minimum of 18 months follow up from Day 0 (Start of Protocolised Prednisolone Regimen) unless a partial remission is not achieved at week 16 or the primary outcome measure event is observed earlier. Participants who achieve the primary endpoint and subsequently enter the Open Label Phase (OLP) will have a minimum of 26 weeks follow up in the OLP.

### Open label phase

Patients randomised to placebo who achieve partial/complete remission by week 16, followed by disease relapse (the primary endpoint event) within 24 months of Day 0 (SPPR) will be invited to participate in the OLP of the trial (Fig. 1).

Data arising from the OLP will not contribute to either the efficacy or safety assessments in the main phase of the trial but will allow exploratory observational analyses, including a comparison of time in remission combining both standard of care or placebo treatment and rituximab treatment.

Those participants in the control arm who consent to the OLP will receive treatment with the intervention described for the rituximab arm in the main trial– i.e. receive rituximab 1 g within 4 weeks of relapse and restarting treatment prednisolone, after 2 weeks (+/-7 days) and, if they continue in remission, after 26 weeks (+/- 2 weeks) of restarting treatment dose prednisolone for relapse. Thus participants will attend 3 additional visits on top of standard care for the rituximab infusions. Serious Adverse Event reviews and concomitant medication reviews will be conducted during these visits.

All participants that enter the OLP will recommence protocolised prednisolone therapy as in the main trial (with the regimen at the discretion of the local investigator).

### Trial objectives

#### Primary objective

• Assess the efficacy of rituximab on the time from partial / complete remission to relapse of NS.

#### Secondary objectives

- Assess the effect of rituximab on NHS and societal resource use and hence cost.
- Assess the effect of rituximab on safety and on secondary measures of efficacy, such as achievement of partial and complete remission of NS and the preservation of renal function.
- Assess the effect of rituximab on health status of participant.

#### Exploratory objective

- To explore the interaction between age of onset with the efficacy of rituximab.
- To explore efficacy of delayed rituximab in NS (data from OLP).

### Trial outcome measures

Primary outcome measure: Time from partial or complete remission (whichever documented first) to relapse of NS as defined as:

- COMPLETE REMISSION (CR): PCR < 50 mg/mmol (only one result required).
- PARTIAL REMISSION (PR): Reduction in Urinary PCR ≥ 50% from Day 0 - SPPR AND PCR ≥ 50 mg/mmol and ≤ 300 mg/mmol on two consecutive results (performed within 14 days of each other). The date of first assessment is the date of partial remission.
- RELAPSE: recurrent NS as defined by albumin < 35 g/l, PCR > 300 mg/mmol and an increase in 100% over the nadir value during remission on two consecutive results (performed within 14 days of each other). The date of first assessment is the date of relapse. For patients with an albumin ≥ 35 g/l prior to relapse, only one PCR result is required (i.e. PCR > 300 mg/mmol that has risen by 100% over the nadir value and an albumin < 35 g/L).

Secondary assessments: will include an evaluation of the effect of rituximab on:

- Proportion of patients achieving partial or complete remission
- Time to partial or complete remission from Day 0 (SPPR)
- Time to treatment failure (not achieving remission by week 16 or relapse) from randomisation
- Serious Adverse Events (SAEs)
- Adverse Events of Special Interest (AESIs), including infection and steroid-associated side effects
- Change in urinary PCR/24 hour proteinuria
- Change in serum albumin
- Kidney function as assessed by the change in glomerular filtration rate (GFR) from Day 0 - SPPR to 24 months and to trial end
- Health Status (EQ-5D-5 L)
- NHS resource use, out of pocket expenditure and morbidity-related lost productivity, (Additional Health Costs)

## Methods: assignment of interventions

Eligible participants are randomly assigned to either the standard of care + placebo.

(control arm) or the standard of care + rituximab (research arm) in a 1:1 ratio using the.

stratified random block method.

Stratification factors are:

- Patient type
- Disease type
- Prednisolone regimen

A web-based central randomisation system allocates each participant a Trial Subject ID.

and treatment kit code.

Participants, and the majority of trial team, including Chief Investigators (CI) and local site teams will be blinded to the treatment allocations. However, there will be an unblinded trial co-ordinator and database manager to oversee the OLP, and the trial statistician and data monitoring committee will also be unblinded. At each site, there will be an unblinded trial pharmacist at each site to prepare IMP.

Emergency unblinding can be performed if required by patient safety. This should be performed by the Principal Investigator, with the site pharmacist and CIs also able to perform emergency unblinding if required.

## Methods: data collection, management and analysis

### Trial assessments

Paper case report forms are used to record trial data which is then transcribed into a bespoken trial database. The following assessments are done at each trial visit, according to the schedule of assessments below:

- Weight
- Blood pressure
- Haematology
- Biochemical analysis
- Urinary PCR
- 24 h urine collection for proteinuria (performed at Month 24 only)
- Concomitant medications - see Sect. 10.3.3
- Adverse event assessments
- Health status questionnaire: EQ-5D-5 L
- Additional Health Costs questionnaire (performed at 12 weeks post remission & then 6 monthly)
- Prednisolone dosing
- Immunoglobulins G and M (performed every 6 months post remission)
- CNI dosing (if applicable) at weeks 4, 8, 12, and 16

### Schedule of assessments

Eligibility of patients for the trial is confirmed with a urine PCR test and a blood test diagnosing nephrotic syndrome. Treatment with steroids start on day 0 of the trial (SPPR) and can be commenced prior to screening and randomisation.

The IMP (rituximab/ placebo) will be administered in 3 doses: dose 1 administered within 4 weeks of SPPR, dose 2 administered 2 weeks later and the dose 3 at week 26. The latter will only be administered if patient achieves remission by week 16.

Pre remission visits take place every 4 weeks with weight, height, blood pressure measurement, urine PCR, blood tests (full blood count, urea, creatinine, e-GFR and albumin), review of adverse events and concomitant medications. A 24 h collection for proteinuria will be done at eligibility screening, week 16, week 24, relapse/ remission confirmation and at month 24. A health status questionnaire will be filled out on each visit. A health cost questionnaires, IgM and IgG blood tests will be carried out every 6 months.

In women of childbearing age, pregnancy tests will be carried out at eligibility screening and within 2 weeks before IMP administration.

Patients are encouraged to test their urine at home and a remission visit will be organised within 2 weeks of detection. Once remission is confirmed, post remission visits are 4 weekly for 12 weeks, and 3 monthly thereon. If patients do not achieve remission by week 16, they will exit the trial.

### Open label phase

If patients achieve remission by 16 weeks, then relapse in the main phase of the trial, they will be unblinded. If they have received placebo, they will be considered for the open label phase.

Eligibility will be assessed for open label, mainly with continued consent from the patients and a negative pregnancy test in female patients of child bearing age. If they choose to proceed, they will receive rituximab, first dose within 4 weeks of relapse, with three doses in total administered in a similar pattern to the main trial. Visits will mirror the main phase, with 4 weekly visits pre remission. Post remission, they will continue with 4 weekly visits for 12 weeks and then 3 monthly thereon. Again, biochemical and urinary parameters will be assessed as before. A health status questionnaire will be filled out on each visit. A health cost questionnaire, IgM and IgG blood tests will be carried out every 6 months.

Patients remain in the open label phase for a minimum of 26 weeks unless they relapse sooner.

Both in the main trial and the open label phase, IMP visits and review visits will be combined wherever possible.

### Covid mitigation

Shortly after TURING opened to recruitment, the COVID-19 pandemic started, resulting in a halt to recruitment for 15 months. The trial restarted recruitment in May 2021, with a number of mitigations. At the discretion of the PI, any trial visit except IMP infusion visits may be done by telephone or NHS-approved videoconferencing facility instead of face to face. Blood and urine tests may be collected at face to face visits, or at more local facilities, if possible. But, if the PI concludes that the risk of attending any healthcare setting is unacceptably high, blood and urine tests not critical for the primary endpoint may be omitted but should be done as soon as the risk level is reduced. Trial participants will have to attend hospital or designated infusion unit to have IMP infusions (as per local policy). Where trial visits and procedures take place over a number of days, the date that laboratory samples are collected shall be taken as the date of the trial visit.

## Statistical methods

### Analysis populations

The following populations will be defined for efficacy and safety analyses:

• Intent-to-treat population (ITT)

The ITT population is defined as all patients randomised in the trial, regardless of whether they actually received treatment. The treatment group will be analysed as randomised.

• Responding patient population

Responding patient population is defined as all patients randomised in the trial, received allocated protocol treatment and achieved partial/complete remission. The treatment group will be analysed as randomised.

• Safety population

The safety population comprises all patients randomised and having received at least one dose of trial treatment. The treatment group will be analysed as treated.

### Primary assessment of the efficacy of rituximab at trial end

The primary objective of the trial is to assess the effect of rituximab on the time from remission to relapse of NS. The most clinically relevant benefit is to maintain remission as long as possible whilst minimising steroid toxicity, that is, the duration of remission in responding patients.

If there is no evidence of difference in the distribution of time to remission between the two arms, the stratified log-rank test stratifying the factors used for randomisation will be used.

Otherwise, the probability-of-being-in-response function (PBRF) will be used as a means of estimating the expected duration of remission (EDoR) across all randomised patients given as the estimated response rate times mean duration of remission for responding patients. The analysis will be based on ITT population.

**Fig. 2:**
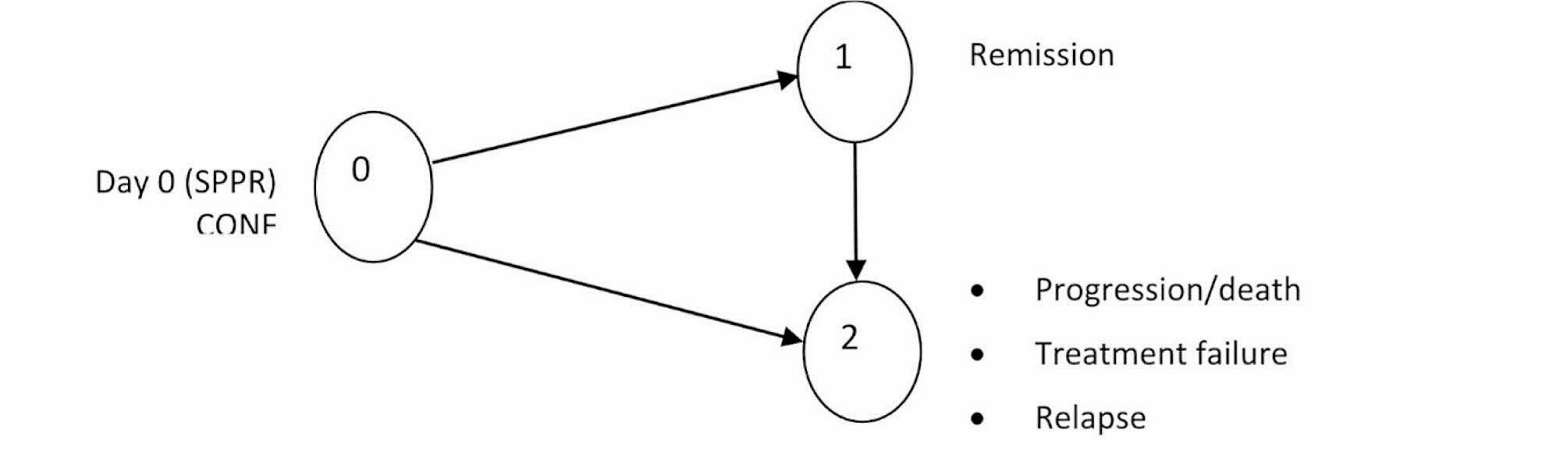
Probability-of-being-in-response function (PBRF)

Briefly, considering a stochastic process in which a patient must start in State 0 (that is at Day 0) and eventually progress to an absorbing state 2 (progression, death in the absence of progression, treatment failure at week 16, relapse (or died from disease), possibly passing through a transient state 1 (remission) (Fig. 2). Response and duration of response are assumed to be independent.

Summary statistics together with the Kaplan-Meier curves for the duration of remission in responding patients by treatment arm and estimated will also be presented. Detailed statistical analysis methods will be included in the statistical analysis plan.

### Censoring

For patients who have achieved partial/complete remission but neither relapsed or died from the disease at the time of the analysis or who are lost to follow up, their data will be censored at the date that they were last known not to have relapsed.

### Safety analysis

The safety analyses will be based on the safety population. All safety parameters will be summarised. Summary tables will be presented for incidence rates (number of patients with at least one incidence) of AESI and SAE.

### Number of patients to be enrolled

The primary outcome measure is time from remission to relapse from partial/complete remission. With 78 patients randomised at a steady rate over a period of 30 months, who subsequently achieved a partial/complete remission, and an additional 24 months follow-up after the last patient randomised (estimated at 54 months after the first patient was enrolled), the trial will have 80% power (two-sided test, significance level of 5%) to show a 50% change in time to relapse from a median value of 9 months in the control arm to 18 months in the research arm, (i.e. a hazard ratio of 0.50). It is expected that the 67 primary outcome measure events required will have occurred at this point. To achieve 85% power (two-sided test, significance level of 5%), 76 events are required. To achieve 90% power (two-sided test, significance level of 5%), 89 events are required.

With an estimated 75% remission rate, allowing for non-compliance of the order of 5%, a minimum of approximately 112 (112*70%=78.4) patients need to be enrolled, 56 in each arm.

Although the effect size of HR = 0.50 upon which sample size calculations are based appears large, two placebo RCTs of rituximab in children with FR NS have given lower HR at 0.267 (CI 0.135–0.528) [19] and 0.02 (CI 0.01–0.15) [20].

The assumptions used in the sample size estimation are being monitored by the Independent Data Monitoring Committee (IDMC). On the recommendations of the iDMC, the sample size was increased to 130–150, to further inform subgroup analyses by disease subtype (MCD and FSGS).

### Economic evaluation

Cost-effectiveness, cost-utility and value of information analyses will be conducted from the perspectives of NHS and society over the trial duration (within-trial analysis), and over a patient lifetime (decision model-based analyses) comparing rituximab versus placebo.

Cost categories will be drugs (sourced from ‘concomitant medications’ measurement), secondary care contacts (sourced from Hospital Episode Statistics), out of pocket costs and lost productivity accrued by the patient plus carer where applicable (patient questionnaires). The NHS perspective is defined as drugs and secondary care contacts. The societal perspective is defined as NHS plus out of pocket costs and lost productivity.

Outcome for the cost-effectiveness analysis will be adapted from the primary outcome (reported as disease and adverse event free period), and for the cost-utility analysis will be quality-adjusted life years (QALYs), using utilities calculated from the Eq. 5D5L using the recommended valuation set at the time of analysis, and integrated with respect to time.

A decision model will combine evidence from the clinical trial with other relevant evidence obtained from the literature to predict longer term costs and outcomes and hence cost-effectiveness. The model will be used to perform a value of information analysis which will predict the expected return on investment from further research into rituximab in MCD/FSGS.

### Ethics and dissemination

Ethical permission has already been granted:

REC reference: 19/LO/0738, Protocol number: CCTU0228, Version 4.4, 28.09.2023.

IRAS project ID: 258,589. Date of initial first favourable REC Opinion: 14 Jun 2019.

EudraCT number: 2018-004611-50.

#### Consent

The investigator or designee ensures that each trial participant is fully informed about the nature and objectives of the trial and possible risks associated with their participation. Written informed consent is obtained from each participant before any trial-specific activity is performed using the ethically approved Informed Consent Form (see supplementary information).

#### Confidentiality

All personal information about potential and enrolled participants will be collected, shared, and maintained in order to protect confidentiality before, during, and after the conduct of the trial, according to the requirements of the EU General Data Protection Regulation (GDPR), Data Protection Act 2018 and Cambridge University Hospital NHS Trust policy.

#### Trial oversight and safety

The IDMC regularly reviews the data from the trial, including safety and efficacy data. This committee reports to the Trial Steering Committee, who oversee the conduct and progress of the trial.

#### Adverse event reporting

SAEs and other adverse events related to steroids or rituximab are regularly reviewed by Trial Management Group (TMG) and IDMC. Due to the underlying clinical condition of the trial population it is not practicable to report all adverse events in this trial and it is thought that excessive safety reporting may detract from the main objectives of the trial. Rather, only AESI are being reported.

AESIs are as follows:

- new onset diabetes mellitus
- osteoporosis/fracture
- peptic ulceration/gastritis requiring medication/endoscopy
- glaucoma
- cataracts
- weight gain > 10% (not attributed to oedema)
- mood changes requiring medication

Patients with NS have frequent hospital admissions related to their underlying disease. Hospital admissions related to hypovolaemia or fluid overload secondary to nephrotic syndrome, or to thromboembolic events, will be exempt from expedited SAE reporting. However, these events will be designated as AESI and details will be collected in the CRFs.

Each Principal Investigator must report all AESIs to the CI using the CRF in a timely manner.

All SAEs should be reported to the CI and TURING trial team using the trial specific SAE form within 24 h of knowledge of the event.

All suspected Adverse Reactions that are related to IMP and that are both unexpected and serious (SUSARs) are subject to expedited reporting. The CI will report all the relevant safety information to the Sponsor, MHRA and the Ethics Committee.

The CI shall inform all investigators concerned of relevant information about SUSARs that could adversely affect the safety of participants.

#### Dissemination policy

Once completed, the results of TURING will be published in a peer reviewed journal, presented at UK Kidney Week, and data will also be made available on request.

### Electronic supplementary material

Below is the link to the electronic supplementary material.

**Supplementary Material 1:** Patient Information sheet and consent form

**Supplementary Material 2:** Complete trial protocol: Version Number: 4.4 Date: 28.09.2023

**Supplementary Material 3:** SPIRIT Checklist for TURING trial

## Data Availability

The full protocol and participant information sheet are included in the supplementary data. Any further trial related information that has obtained ethical approval may be obtained by emailing add-tr.turing@nhs.net. The full anonomised data set will be available from the TURING team at the Cambridge Clinical Trial Unit once the trial has been completed and published.
