# Supplementary material for: A randomised, two-arm (1:1 ratio), double blind, placebo controlled phase III trial to assess the efficacy, safety, cost and cost-effectiveness of Rituximab in treating de novo or relapsing NS in patients with MCD/FSGS (TURING)
Source: BMC Nephrol. 2024 Aug 7;25:253. doi: 10.1186/s12882-024-03576-0 (PMC11308314; doi:10.1186/s12882-024-03576-0)
Supplement: Supplementary file 1 — Supplementary Material 1: Patient Information sheet and consent form [file 12882_2024_3576_MOESM1_ESM.pdf]

PARTICIPANT INFORMATION SHEET & INFORMED CONSENT FORM

The **U**se of **Rituximab I**n the Treatment of **N**ephrotic **G**lomerulonephritis (TURING)

You are being invited to take part in a research trial. Before deciding whether to take part, you need to understand why this research is being done and what it involves. Please take time to read the following information carefully and talk to others about the trial if you wish. Please ask us if anything is not clear or if you would like more information. Please take time to decide whether or not you wish to take part.

Section 1 tells you the purpose of this trial and what will happen to you if you take part.
Section 2 gives you more detailed information about the conduct of the trial.

Section 1: Purpose of the trial and what will happen

1. What is the purpose of the trial?

You have a condition called nephrotic syndrome caused by Minimal Change Disease (MCD) or Focal Segmental Glomerulo-Sclerosis (FSGS). This causes the kidneys to leak protein into your urine, which means the protein level in your blood goes down, and you may develop swelling of your legs and face. The precise cause is unknown, but we do know that it is an autoimmune disease. This means your immune system, which would normally fight infections, is overactive and causing your kidneys to leak the protein. If left untreated, nephrotic syndrome can be very serious, resulting in an increase in blood clots, infections and even kidney failure.

Standard treatment for this disease is with oral steroids (prednisolone). This is effective in most patients, but often the disease comes back (relapses) and repeated courses of treatment are needed which can cause severe side effects. Rituximab is a drug which has been used in children with nephrotic syndrome, and has been shown to be effective in preventing relapses of the disease and increasing the time between relapses. Small studies in adults suggest it may also prevent disease relapse in adults too, but it is not known how effective it is, or how often the rituximab should be given.

The purpose of this trial is to test whether giving rituximab is effective in preventing relapses of the disease. It will also tell us how long patients remain well after the rituximab treatments are stopped, and whether repeated treatment with rituximab is safe. The trial will also look at the costs of rituximab to the NHS, and compare the incurred costs with the treatment effects to determine whether rituximab represents a good use of NHS funds. It is hoped that the results of this trial will help doctors decide the best course of treatment for future patients with new or relapsing nephrotic syndrome.

2. What is the drug being tested?

Rituximab is a medicine used to reduce the activity of the immune system when it is overactive. It contains a protein called a 'monoclonal antibody' which sticks to the surface of a specific type of white blood cell called a 'B-cell'. When rituximab sticks to the surface of this cell it dies, reducing the over activity of your immune system which is causing your disease. It is a licensed treatment for other autoimmune diseases, such as rheumatoid arthritis and a type of vasculitis known as ANCA associated vasculitis, where it seems to be generally well tolerated. The risk of severe infections is low in patients who take rituximab and rituximab may therefore

be safer than the repeated courses of steroids currently used to treat nephrotic syndrome.

You will also receive steroids (prednisolone), which are a standard treatment for nephrotic syndrome. Steroids are a type of drug that has been used in the treatment of nephrotic syndrome for 60 years. You may already have been prescribed these previously as part of your standard care. In this trial, they will be given in tablet form and the dose will decrease (taper) over time, according to how your disease is responding. You will be provided a diary to fill in with number of tablets you take each week – this is not mandatory to do. If you decide to use this paper diary, your trial doctor will explain how to fill it in. You will need to bring this diary with you to every visit until you have stopped taking steroid completely.

If you are on medication which suppresses your immune system like Calcineurin inhibitors, you will be gradually taken off these over the first few weeks of being on this trial. If you are taking certain other immunosuppressant medicines, you may be asked to stop taking these at the start of the trial.

Please note: Covid-19

Covid-19 is caused by a viral infection affecting the lungs that has arisen recently and affected many people around the world. Although we don't know whether patients with nephrotic syndrome have an increased risk of more severe disease, there is evidence that patients who are immunosuppressed after an organ transplant tend to get sicker. We also think that high dose steroids increase the risk of a worse outcome. We do not yet know whether rituximab increases the risk of a more severe infection from Covid-19. However, in other viral infections such as flu, rituximab does not seem to make people more susceptible or to have worse disease.

If rituximab is effective in treating nephrotic MCD/FSGS, it will result in patients needing fewer courses of steroids, which could be beneficial if Covid-19 continues to be widespread across the UK.

Entering the trial will mean you have to make a maximum of 3 extra trial visits in the main part of the trial, and a further 3 if you enter the open label phase. This may increase the risk of you coming into contact with the virus, although your hospital will reduce the risk as far as possible.

One other possible risk of rituximab is that it may reduce your body's ability to make protective antibodies to the virus for up to a year after the last dose of rituximab. This may mean that you might be more likely to catch the virus a second time, and that you may not be as protected by a vaccine as if you had not had rituximab.

3. Why have I been invited?

You have been invited to participate in this trial because you have been diagnosed with nephrotic syndrome (shown to be caused by Minimal Change Disease (MCD) or Focal Segmental GlomeruloSclerosis (FSGS) on your kidney biopsy taken previously). You are experiencing your first episode of nephrotic syndrome or are experiencing a relapse of your disease.

For this trial, we plan to include 112 patients with new or relapsing nephrotic syndrome secondary to MCD or FSGS from approximately 30 to 40 hospitals across the UK.

4. Do I have to take part?

Participating in this trial is completely voluntary. You will have the opportunity to speak to the trial team and ask any questions you have about the trial. If you decide to participate you will be asked to sign an Informed Consent Form. However, you are still free to change your mind and leave the trial at any time without giving a reason. If you choose not to participate or to leave the trial, your future medical treatment and standard of care will not be affected in any way.

5. What will happen to me if I take part?

As you are already showing symptoms of nephrotic syndrome, you will be prescribed steroid treatment right away and this will not affect your participation in the trial.

Informed Consent and Eligibility

Your trial doctor will explain the trial to you and you will be given enough time to decide whether you would like to take part in the trial. You will have the opportunity to ask any questions you may have about the trial and to discuss these with your doctor. If you agree to participate, you will be asked to sign the consent form at the end of this information sheet and be given a copy of this to take away and refer to later.

Eligibility for trial participation will be checked after you have given consent to do so. If you are a woman who is able to get pregnant, you will be asked to have a pregnancy test, which will be done with your usual clinic blood tests (approximately 1 teaspoon of blood). We are unable to include pregnant women in this trial.

If you meet all the eligibility criteria, you will be randomly allocated, by a computer, to one of two, equal sized groups (randomised). You will have a 50% chance of being in either of the following two treatment groups:

i. **Standard Care and Rituximab Group:** If you are assigned to this group you will receive all the usual treatment for nephrotic syndrome as recommended by your doctor. In addition, you will also be prescribed the trial drug, rituximab, which will be given via a drip in your hand or arm. Before each dose, you will also receive a solution of intravenous steroids and anti-histamines to reduce the chance of any rituximab- related infusion reactions. You will receive **three** doses of rituximab in total. The first dose will be given at the beginning of the trial, the second dose one to four weeks later and the third dose after approximately six months.

ii. **Standard Care and Placebo group:** If you are assigned to this group you will receive all your usual treatments as recommended by your doctor. In addition, you will be prescribed a placebo infusion. This is sometimes called the 'dummy drug'. It looks the same as the treatment but does not contain any of the active ingredients. To keep the groups the same except for the rituximab treatment, you will also receive a solution of intravenous steroids and anti-histamines which reduces the chance of reactions to the rituximab therapy. You will receive **three** doses of placebo in total. The first dose will be given at the beginning of the trial, the second dose one to four weeks later and the third dose after approximately six months.

While you participate in the trial, neither you nor your doctor will be told which treatment group you have been assigned to. This is called a double-blind trial. However, in an emergency, your doctors can find out which treatment you are receiving if necessary.

All patients will be reviewed regularly in the trial. If your disease has not improved sufficiently with treatment 16 weeks after starting your steroid treatment, you will not continue in the trial and your doctor will discuss other treatment options with you. You will not then have the third treatment infusion (of either rituximab or placebo) after six months.

Trial Visits

Once you have provided consent, the trial doctor or nurse will record your demographic information including height, weight, date of birth, and race. This will happen in the clinic you are attending for your usual treatment and will not usually require any additional visits. Your trial doctor will prescribe the medicines you need to treat your nephrotic syndrome and book for you to come in for your first dose of rituximab or placebo. You will have your blood tests done in the clinic in the usual way; there will not be any extra blood tests required for this trial however, we will use the results from these tests to ensure that it is safe for you to continue in the trial.

For the duration of the trial, we will carry out further trial assessments at your routine clinic reviews every four weeks until you go into remission, then every four weeks for the first three months and then every three months for a minimum of two years. You will also have a trial visit if your nephrotic syndrome has relapsed. Your doctor may ask to see you more frequently in the clinic in addition to this if he/she thinks this is required to monitor your condition.
Assessments to be performed throughout the trial will include:

- Routine blood and urine tests for your condition and to ensure it is safe for you to continue in the trial
- Urine tests to help us monitor the effect that the drug might be having on your condition (research samples)
- Blood pressure measurements
- A review of any other medication you may be taking
- A review of any side affects you may have experienced
- Short questionnaires at every clinic visit
- Review of steroid tapering diary

If you have a telephone/video assessment, instead of attending the hospital, then we will arrange for your blood and urine tests to be done at your GP surgery or another clinical setting, and the results forwarded to us electronically.

Questionnaires

At certain points during this trial you will be asked to complete a short questionnaire about your quality of life and one about any out-of-pocket costs and related issues incurred because of your nephrotic syndrome.

You will be asked questions about the following topics: Mobility, Self-Care, Usual Activities, Pain and Discomfort, Anxiety and Depression and overall how good or bad you feel your health is. This is so we can get an idea if you feel your health is currently impacting on your quality of life, and whether this is affected by the rituximab therapy. You will need to complete this short questionnaire about your quality of life at every visit.

You will also be asked whether you are receiving any additional care or complementary or alternative medicine paid for either by yourself or private health insurance, any time spent by family or friends helping you with activities such as feeding, bathing, housekeeping and transportation, and how your ability to work and do your usual activities is affected by your nephrotic syndrome. This will let us know whether any out of pocket costs and your ability to do your usual activities are affected by the kidney disease. You will be provided with a diary which you can complete with any additional information you would like us to know.

These questions will be asked at the trial visits at the beginning of the trial, and then every three months until the end of the trial. The questionnaires should take no more than 20 minutes to complete and a member of the research team will be present should you require any assistance. If your trial visit is completed over the phone then these questionnaires will either be posted to you with a pre-paid returns envelope or your trial nurse will complete the questionnaire for you while asking you questions over the phone.

Urine Collection

In addition to the usual small sample of urine you are asked to bring to your clinic appointments, you will be asked to collect your urine for 24 hours, at four different points during the trial (once at the beginning, once at 16 weeks, once at 24 months and if your nephrotic syndrome recurs). The research nurse will give you the container for collection and will ask you to bring this back to the clinic when you next come. You will be asked to collect all the urine you produce over 24 hours starting from the second urine of the day till the first urine of the following day. You can keep this container in your bathroom at room temperature. You must return this urine sample to your trial team within one week of collection.

If your disease responds to treatment and you are in complete remission you will also be encouraged to test your urine for protein when you are home on a weekly basis. Your doctor will provide you with dipstick tests for this purpose. Your doctor will also provide you with a paper diary which you will use to record your protein levels. The dipsticks will display a result with either colours or '+' symbols. If you have a result of '+++' or '++++' you should re-test your urine the next day. If the result is still '++++' then please contact your trial team and arrange to meet your trial doctor. These instructions are also repeated in the diary to help you remember what to do next. Please bring this diary along to all your hospital visits for a review by the trial team.

After you have been told you have achieved partial remission, please let your local trial doctor know if you feel your disease is worsening.

Steroid tapering diary completion - optional

Your trial team can provide you with a paper diary to take note of the number of tablets of steroids you are taking weekly. You do not have to complete this diary but it will be helpful to collect this information. You will need to bring this diary with you to all your trial visits until you are no longer taking any steroids.

Visits for Rituximab or placebo

Rituximab or placebo will be given via a needle which is put into a vein in your arm. The needle is connected to a bag ('a drip') which contains the treatment and the

needle is taken out of your arm at the end of the visit. This type of drug administration is called an infusion. The infusions of rituximab or placebo will happen at the beginning of the trial, one to four weeks later and at six months (if your disease has improved after 16 weeks of treatment in the trial). Each infusion will take approximately 4 to 6 hours. You will be monitored for any drug reactions by the trial doctor/research nurse. After your infusion, you can resume your normal activities straight away.

Completion of the Trial and Open Label Phase

You will be followed up for at least two years after entry into the trial or until your nephrotic syndrome relapses if this happens sooner.

Some patients may go into remission during the trial and no further treatment may be required once you have completed the trial.

If you were originally allocated placebo in the trial you may be eligible to receive rituximab if your disease initially responds to steroid treatment by 16 weeks but you then experience a relapse during the two year follow up period of the trial. If you agree to participate in this part of the trial, your trial doctor will find out via the trial database if you were given placebo and then prescribe three doses of rituximab for you. These three doses will be given over 6 months; the first two doses will be given within one to four weeks of each other and the final dose approximately 6 months from when you begin the open label phase. At these visits you will only have rituximab infusions. In addition you will also be required to complete questionnaires at every visit except the infusion visits. These questionnaires will not take more than 20-30 minutes of your time and your research nurse will be available to answer any queries you may have. Your usual blood and urine test results will be reviewed to ensure you are able to continue treatment. If you are female and of child bearing potential, you will need to have a blood test to ensure you are not pregnant as rituximab cannot be given to pregnant women. Your doctor will also review your steroid tapering diary and review your medication chart.

In this open label phase, you will be seen for clinic visits every 4 weeks until you achieve remission. When you are in remission you will be seen every 4 weeks for 12 weeks and then every 3 months until you relapse or until the trial ends whichever happened sooner.

Your standard blood and urine test results will be recorded by trial team as part of trial data. This information will be also be collected from RaDaR. RaDaR is run by the UK Renal Association and is linked to the UK Renal Registry (UKRR). It is a UK wide registry collecting long term health data from all enrolled patients.

If it is determined that you already received rituximab as part of the trial, once you have completed your trial visits, you will return to standard clinical care locally.

Your trial doctor may also discuss other trials with you that might be of interest to you, like the NephroS trial. If you are interested in participating, your trial doctor can provide a separate information sheet and consent form.

6. What will I have to do?

Medication

You will need to attend the hospital for the infusions of rituximab or placebo and to take all the other medications as prescribed by your doctor in clinic. You should continue using any regular prescription medicine from your GP– we will check this list with you prior to starting the trial and throughout treatment, but you **must** inform your trial doctor if any changes are made by your GP. You should tell the trial team if you feel unwell or different in anyway. If you have any major concerns or are feeling very unwell, please contact the trial team using the contact numbers at the end of this information sheet.

Participation in this trial may affect any existing life insurance, critical illness or income protection insurance which you have and you should discuss your participation in this trial as failure to notify them could affect or invalidate your cover. Private medical insurance does not usually cover the cost or consequences of taking part in any clinical research so you may also wish to discuss your participation in this trial with any private medical insurance provider you have.

Contraception

Please share this information with your partner if it is appropriate.

Rituximab could harm an unborn baby or nursing infant. You will not be able to take part in this trial if you are pregnant or breastfeeding. You should not participate in this trial if you are planning to become pregnant during the trial.

Women of childbearing potential must use one of the following, reliable forms of contraception for 12 months after your last treatment with rituximab/placebo:

Oral contraceptive (either combined or progestogen alone)
- Contraceptive implant, injections or patches
- Vaginal ring
- Intrauterine device (IUD, coil or intrauterine system)
- Condom **and** cap or diaphragm **plus** spermicide (chemical that kills sperm)
- True abstinence where this reflects your usual and preferred lifestyle

You do not need to use contraception if:
- you have only one partner, and the man has had an operation to cut the tubes that carry sperm (vasectomy)
- you (or your partner) are a woman who cannot become pregnant
- You practice true abstinence as part of your usual and preferred lifestyle (no sexual activity from the day you consent to take part in the trial and until 12 months after the last dose of trial medication). If you become sexually active, you must use one of the methods listed above.

If you become pregnant during the trial or within 12 months of having rituximab or placebo, you should inform your trial doctor immediately. Your trial doctor will discuss all the options available to you. The outcome and progress of any pregnancy would be followed and you would be asked questions about the pregnancy and baby, if appropriate.

7. What are the side effects of the drug being tested?

Some patients sometimes experience side effects when they take medication. These are organised into 'Common', 'Uncommon' and 'Rare'. If you get any of these side effects your doctor will discuss this and try to help.

Rituximab:

Very Common (10% and more of patients)
Infections such as pneumonia (bacterial), pain on passing water (urinary tract infection). Allergic reactions that are most likely to occur during an infusion, but can occur up to 24-hours after infusion, with changes in blood pressure, nausea, rash, fever, feeling itchy, runny or blocked nose and sneezing, shaking, rapid heartbeat, and tiredness, headache. During the infusion, the nurses looking after you will be monitoring you closely for any of these reactions. The frequency of these reactions decreases during subsequent infusions. You will be given steroids and anti-histamines through a vein prior to receiving the infusion, to minimise these side effects, and may also be given oral paracetamol and antihistamines.
Changes in results of laboratory tests carried out by your doctor. These include a decrease in the amount of some specific proteins in the blood (immunoglobulins) which help protect against infection.

Common (between 1% and 10% of patients)
Infections such as bronchial tube inflammation (bronchitis), a feeling of fullness or a throbbing pain behind the nose, cheeks and eyes (sinusitis), pain in the abdomen, vomiting and diarrhoea, breathing problems, fungal foot infection (athlete's foot), high cholesterol levels in the blood, abnormal sensations of the skin, such as numbness, tingling, pricking or burning, sciatica, migraine, dizziness, loss of hair, anxiety, depression, indigestion, diarrhoea, acid reflux, irritation and /or ulceration of the throat and the mouth, pain in the tummy, back, muscles and/or joints.

Uncommon (less than 1% of patients)
Severe infusion reactions, including allergic reactions with shortness of breath, swelling of the face and tongue, collapse.
A reduction in white blood cells which does not happen straight away. Heart problems – such as reduced heart rate or chest pain (angina), heart failure, heart attack, uneven or fast heart rate.

In less than 0.01% patients, rituximab increases the risk of getting a rare but very serious (possibly fatal) brain infection (progressive multifocal leukoencephalopathy-PML). If you develop loss of coordination, weakness, confusion, difficulty talking /walking seizure or vision changes please contact your trial team urgently.
Use of rituximab has been also associated with two very rare, but life threatening skin reactions. Redness, often associated with blisters, may appear on the skin or on mucous membranes, such as inside the mouth, the genital areas or the eyelids, and fever may be present. **Tell your doctor immediately if you have any of these symptoms.** Some of the skin reactions occurred on the day of the infusion or within a few days of the infusion. However in some cases, the event occurred weeks or months after infusion. You will be monitored for such reactions. If these reactions were to occur, your trial doctor may stop your rituximab treatment, and will treat you according to local practice at your hospital. If you feel generally unwell, and have any **new** joint pains, fevers, or skin lesions, please contact your trial team immediately.

Steroids:

All patients will receive steroids for standard treatment of their nephrotic syndrome. Steroids can cause thinning of the bones and skin as well as weight gain, high blood pressure, diabetes, muscle wasting and eye cataracts over a long period of use.

We do take precautions to avoid these side effects. For example, initially whilst receiving high dose steroids your trial doctor will give advice on the protection of your bones against osteoporosis, and your stomach against ulcers and you may be given an antibiotic to help prevent infections. We will also monitor your blood test results closely to check for any changes in your liver function. Occasionally the extra treatments used to prevent problems may themselves have side effects. Your trial doctor will inform you of these depending on the treatments used.

8. What are the possible disadvantages and risks of taking part?

Wherever possible we will book your trial visits at the same time as your routine hospital visits. However, you will be required to come into the hospital for 3 separate visits at specific times for the infusions. The trial treatment/placebo infusions take between 4 to 6 hours to complete and you will be required to stay in hospital during this time. It is possible you may experience bruising or soreness at the needle site of the infusions.

As mentioned in question 2 of this information sheet, trial visits will take place in a hospital and this may increase your chances of coming in contact with the Covid-19 virus.

Please talk to your trial doctor before considering having any vaccinations whilst participating in this trial as certain vaccines including all live vaccines should not be taken with the trial medication. However, if a vaccine against Covid19 is developed using a live vaccine that is considered safe in immunosuppressed patients it would be appropriate for you to be vaccinated. Your trial doctor will be able to advise you further.

9. What are the possible benefits of taking part?

There is no guarantee you will benefit from taking part in this trial, although it is expected that the treatment you receive as part of the trial will help to control your nephrotic syndrome in the short term. The information collected from your participation in this trial may help doctors to make decisions about treating patients with nephrotic syndrome secondary to MCD/FSGS in the future.

However, the weekly urine dipstick test results can alert you to a relapse and help you receive treatment faster.

10. What are the alternatives for treatment?

There are alternative treatments for nephrotic syndrome that suppress the immune system, including repeated course of steroids alone, treatment with Calcineurin inhibitors such as tacrolimus and cyclosporine, alkylating agents such as cyclophosphamide, and antiproliferative drugs such as mycophenolate mofetil. Your doctor will discuss whether these would be suitable for you as an alternative to entering the trial.

11. What happens when the trial stops?

If you have achieved remission and your disease has not relapsed during the trial, no further treatment will be required and you will return to standard clinical care locally.

If your nephrotic syndrome initially responds to treatment and then relapses during the trial, and you were allocated to the placebo arm, you may be eligible for treatment with rituximab as part of this trial (and be able to enter the open label phase as described in section 5 above).

If you have already been treated with rituximab during the trial, you may or may not be able to have further treatment with rituximab outside of the study. Your doctor will be able to discuss with you what is available to you.

As part of the trial, your disease will be assessed 16 weeks after entering the trial. If your disease has not responded sufficiently to treatment, you will not continue in the trial as it is likely this is not the best treatment for you. In this event, your doctor will discuss alternative treatments with you.

12. Expenses & Payment?

You will be reimbursed reasonable expenses incurred for travel to hospital for the infusion visits as well as refreshments during your visits.

Section 2: Trial Conduct

13. What if new information becomes available?

Sometimes during the course of a trial, new information becomes available which might affect your decision to continue participating in this trial. Your trial doctor will contact you to discuss the new information and whether you wish to continue participating in the trial. If you still wish to continue on the trial, you will be asked to sign a new Informed Consent Form.

The trial sponsor, the regulatory authority or the trial doctor may decide to stop the trial at any time. If that happens we will tell you why the trial has been stopped and arrange for appropriate care and treatment for you.

14. What if I decide I no longer wish to participate in the trial?

You are free to stop participating in this trial at any time without giving a reason and without affecting your future care or medical treatment. If you decide not to participate any further, you will no longer receive the trial treatment. No further tests will be performed on you and no further research samples will be collected. Any data already collected or results from tests already performed on you or your samples will continue to be used in the trial analysis.

The trial doctor may also choose to withdraw you from the trial if they feel it is in your best interests or if you have been unable to comply with the requirements of the trial. Reasons for trial withdrawal could include:

- You have experienced a serious side effect
- You are unable to complete the visits, medication or trial documentation as required
- You become pregnant or plan to become pregnant
- The trial doctor feels you no longer appear to benefit from the treatment.
- If you are unwilling to attend hospital for infusion visits as per the trial schedule

If you have experienced any serious side effects during the course of the trial which require you to withdraw from the trial, your trial doctor will follow-up with you regarding your progress until the side effect has stabilised or resolved.

15. What if there is a problem?

Any complaint about the way you have been dealt with during the trial or any possible harm you might suffer will be addressed. If you have any concerns about any aspect of this trial you should speak to your trial doctor who will do their best to answer your questions.

In the event that something does go wrong and you are harmed by taking part in the research and this is due to someone's negligence then you may have grounds for a legal action for compensation against Cambridge University Hospitals. If your claim is successful your legal costs will be met. The normal National Health Service complaints mechanisms will still be available to you (if appropriate).

The NHS does not provide no-fault compensation i.e. for non-negligent harm, and NHS bodies are unable to agree in advance to pay compensation for non-negligent harm. They are able to consider an ex-gratia payment in the case of a claim.

If you wish to complain or have any concerns about any aspect of the way you have been approached or treated during this trial, you can do this through the NHS complaints procedure. In the first instance it may be helpful to contact the the Patient Advice and Liaison Service (PALS) at your hospital.

16. Will my taking part in this trial be kept confidential?

Cambridge University Hospitals NHS Foundation Trust (CUH) is the Sponsor for this clinical trial based in the United Kingdom. They will be using information from you and your medical records in order to undertake this trial and will act as the data controller for this trial. This means that they are responsible for looking after your information and using it properly. The Sponsor organisation(s) will keep identifiable information about you for 5 years after the trial has finished ensuring your safety and allowing the trial to be reviewed by the authorities after it is finished.

Your rights to access, change or move your information are limited, as the Sponsor organisation(s) need to manage your information in specific ways in order for the research to be reliable and accurate. To safeguard your rights, we will use the minimum personally-identifiable information possible.

You can find out more about how the Sponsor (s) use(s) your information using the information below:

- For Cambridge University Hospitals NHS Foundation Trust, please visit: https://www.cuh.nhs.uk/corporate-information/about-us/our-responsibilities/looking-after-your-information, or email the Data Protection Officer at: gdpr.enquiries@addenbrookes.nhs.uk

For participants recruited at CUH:
Cambridge University Hospitals will collect your name, NHS number, contact details and date of birth to contact you about this trial, and make sure that relevant information about the trial is recorded for your care, and to oversee the quality of the trial. Individuals from the Sponsor and regulatory organisations may look at your medical and research records to check the accuracy of this trial. Cambridge

University Hospitals will pass these details to the Sponsor along with the information collected from you and your medical records. The only people in the Sponsor organisation who will have access to information that identifies you will be people who need to contact you in relation to this trial and to audit the data collection process. Cambridge University Hospitals will keep identifiable information about you from this trial for 5 years after the trial has finished.

For participants recruited at other participating sites:
Your site will keep your name, NHS number, contact details and date of birth to contact you about this trial, and make sure that relevant information about the trial is recorded for your care, and to oversee the quality of the trial. Certain individuals from the Sponsor and regulatory organisations may look at your medical and research records to check the accuracy of this trial.

Your site will keep identifiable information about you from this trial for *5* years after the trial has finished.

We will pass personal information about you (name, NHS number and contact details, date of birth) to the Sponsor organisation to collect and monitor your blood and urine results via the renal registry RaDaR. Your identifiable information will be stored on secure University of Cambridge servers and access will be limited to a small number of authorised individuals who are responsible for the central management of the trial.

The TURING trial will be linked to RaDaR and UKRR to allow health data from trial participants to be included in the trial database. The data is held on UKRR servers in two separate Data Centres. Only substantive employees of the Renal Association working within the Renal Registry function have access to this data. The data centres host the hardware and provide connectivity. Data will be collected to allow follow up for trial participants, including from the Open Label Phase. This data collection will ensure data we collect about your kidney function is as complete as possible. The data will then be destroyed in accordance with the trial regulations in place at the time.

If you consent to taking part in the TURING trial, you will be required to sign a consent form which adds you to this registry if you have not already done so.. You can choose to withdraw from this registry at any point in time after participation in TURING.

Your data will also be collected from NHS Digital via the Hospital Episode Statistics (HES). The NHS collects information on all hospital admissions, including when, why and for how long they happen. In England, this is known as **Hospital Episode Statistics (HES)**. By collecting information from HES, we can tell what happens to the health of participants in this trial. For example, if someone suffers a heart attack, this should result in admission to hospital and would show up in the information we collect. By doing things in this way, it means that we can use the information the NHS already holds rather than having to ask patients to attend hospital for regular extra study visits.

By consenting to this trial, you agree that the trial team will provide your personal data (your full name, NHS/CHI number, date of birth and gender) to NHS Digital for linkage to HES data.

Equivalent systems to HES exist in Wales (**Patient Episode Database for Wales, PEDW**) and Scotland (**Information Services Division Scotland**, **ISD**). If you live in these areas, the trial team will similarly obtain information on hospital admissions from these sources.

Once you have agreed to participate in this trial you will be allocated a Trial ID Number. This is a unique trial number which will be used on all your trial

documentation along with your date of birth. Your date of birth is considered to be personal information. We collect this personal information on trial documentation to help ensure that the data we receive as part of your trial participation is correctly allocated to you. By cross checking these two unique references we can ensure the integrity of the data.

Your personal and medical information will be kept in a secured file and be treated in the strictest confidence.

This information will not identify you and will not be combined with other information in a way that could identify you. The information will only be used for the purpose of health and care research, and cannot be used to contact you or to affect your care. It will not be used to make decisions about future services available to you, such as insurance.

All trial related information (data) from your trial visits will be sent onto the trial centre in Cambridge. All research data collected in this way will be stored on highly secure encrypted servers held within the University of Cambridge and will be accessible only to the team of researchers directly involved with the trial.

Only anonymous trial data, without any personal information will be published at the end of the trial.

Your trial doctor may also discuss other similar trials like the NephroS trial with you. If you are interested in participating, your trial doctor can provide a separate information sheet and consent form.

We will need to inform your GP of your participation in this trial so that any medical decisions made by your GP account for any treatment you are receiving as part of this trial.

17. What will happen to my samples?

All blood and urine samples will be processed in your local hospital laboratory and the results will be sent onto your local trial team. All samples will be destroyed after these tests as per local practice.

The urine you use to measure protein levels at home can be discarded down the toilet once you have got a result from the dipstick.

18. What will happen to the results of the trial?

The results of the trial will be anonymous and you will not be able to be identified from any of the data produced. When the results of this trial are available they may be published in peer reviewed medical journals and used for medical presentations and conferences. They will also be published on the EU Clinical Trials Register website, a central registry for all clinical trials conducted in the EU.

Anonymous datasets from the trial may also be made available to other researchers in line with national and international data transparency initiatives.

19. Who is funding the trial?

The trial is being funded by the National Institute for Health Research (NIHR).

20. Who has reviewed this trial?

All research within the NHS is reviewed by an independent group of people called a Research Ethics Committee, to protect your interests. This trial has been reviewed and given favourable opinion by the London - City & East Research Ethics Committee. The Medicines and Healthcare Products Regulatory Agency (MHRA) who are responsible for regulating medicines in the UK have also reviewed this trial.

21. Further information and contact details

If you have any questions concerning this trial, please contact your:

Trial Doctor
Name: Dr Lisa Willcocks
Telephone: 01223 217180

Research Nurse
Name: Wanwanauch Sumransukrat (Yok)
Telephone: 01223 256429

In the event of an emergency please contact:

24-hour contact details
Contact: Dr Lisa Willcocks/ Renal Registrar on call
Tel: +44 (0)1223 245151 (Addenbrooke's main hospital)

If you wish to complain or have any concerns about any aspect of the way you have been approached or treated during this trial, you can do this through the NHS complaints procedure. In the first instance it may be helpful to contact the Patient Advice and Liaison Service (PALS) at your hospital.

Patient Advice and Liaison Service (PALS) at:
Hospital Name: Cambridge University Hospitals NHS Foundation Trust
Tel: 012230 216756 / pals@addenbrookes.nhs.uk

Cambridge University Hospitals NHS Foundation Trust

TURING

INFORMED CONSENT FORM

Trial Title: **T**he **U**se of **Rituximab** In the **T**reatment of **N**ephrotic **G**lomerulonephritis (TURING)

Principal Investigator: **Participant Number:** _________

If you agree with each sentence below, please initial the box **INITIALS**

1	I have read and understood the Participant Information Sheet version 3.2 dated 04/08/2023 for the above trial and I confirm that the trial procedures and information have been explained to me. I have had the opportunity to ask questions and I am satisfied with the answers and explanations provided.	
2	I understand that my participation in this trial is voluntary and that I am free to withdraw at any time, without giving a reason and without my medical care or legal rights being affected.	
3	I understand that personal information about me will be collected and used in accordance with this information sheet. This information will be kept in the strictest confidence and none of my personal data will be published.	
4	I understand that sections of my medical notes or information related directly to my participation in this trial may be looked at by responsible individuals from the sponsor, regulatory authorities and research personnel where it is relevant to my taking part in research and that they will keep my personal information confidential. I give permission for these individuals to have access to my records.	
5	I understand that my GP will be informed of my participation in this trial and sent details of the TURING trial.	
6	I understand that my personal data might be transferred between the trial team at different trial sites in relation to my participation in this trial. I understand that any personal data will be sent using secure mail servers.	
7	I have read and understood the compensation arrangements for this trial as specified in the Participant Information Sheet.	
8	I understand that the doctors in charge of this trial may close the trial, or stop my participation in it at any time without my consent.	
9	I have read and understood my responsibilities for the trial including using appropriate contraception as listed in section 6.	
10	I understand that I will be added to the RaDaR registry to allow long-term follow up data to be collected.	

OPTIONAL

		YES	NO
12	I consent to be approached to take part in the open label extension.		
13	I consent to being approached about other research studies.		

I agree to participate in the main phase/open label phase of the trial (delete as appropriate):

Name of patient	Signature	Date
Name of person taking consent	Signature	Date

Time of Consent (24hr clock) ________:________

1 copy for the patient, 1 copy for the trial team, 1 copy to be retained in the hospital notes.
