# Supplementary material for: A randomised, two-arm (1:1 ratio), double blind, placebo controlled phase III trial to assess the efficacy, safety, cost and cost-effectiveness of Rituximab in treating de novo or relapsing NS in patients with MCD/FSGS (TURING)
Source: BMC Nephrol. 2024 Aug 7;25:253. doi: 10.1186/s12882-024-03576-0 (PMC11308314; doi:10.1186/s12882-024-03576-0)
Supplement: Supplementary file 2 — Supplementary Material 2: Complete trial protocol: Version Number: 4.4 Date: 28.09.2023 [file 12882_2024_3576_MOESM2_ESM.pdf]

The **U**se of **R**ituximab **I**n the treatment of **N**ephrotic **G**lomerulonephritis

CLINICAL TRIAL PROTOCOL

**Trial Title:**	A randomised, two-arm (1:1 ratio), double blind, placebo controlled phase III trial to assess the efficacy, safety, cost and cost-effectiveness of rituximab in treating *de novo* or relapsing NS in patients with MCD/FSGS (**TURING**)
**EudraCT Number:**	2018-004611-50
**ISRCTN Number:**	ISRCTN 16948923
**Investigational Product:**	Rituximab
**Protocol Version:**	Version 4.4

**Chief Investigator:**	Dr Lisa Willcocks
Address:	Cambridge University Hospitals NHS Foundation Trust
Telephone:	01223 245151
**Co-Lead Investigator:**	Prof Megan Griffith
Address:	Imperial College NHS Trust
Telephone:	02083835272

**Trial Sponsor:**	Cambridge University Hospitals NHS Foundation Trust
**Trial Funder:**	National Institute for Health Research

**SAE Reporting:**	Cambridge Clinical Trials Unit (CCTU)<br>Unblinded Trial Coordinator<br>Telephone: 01223 254684<br>Email: add-tr.turingolp@nhs.net

1 PROTOCOL SIGNATURES

I give my approval for the attached protocol entitled "The Use of Rituximab In the treatment of Nephrotic Glomerulonephritis (TURING)" dated 28.09.2023.

Chief Investigator

Name: Dr Lisa Willcocks

Signature: ________________________

Date: ___________________________

Trial Statistician

Name: Dr Wendi Qian

Signature: _________________________

Date: _____________________________

Site Signatures

I have read the attached protocol entitled "The Use of Rituximab In the treatment of Nephrotic Glomerulonephritis (TURING)" dated 28.09.2023 and agree to abide by all provisions set forth therein.

I agree to comply with the conditions and principles of Good Clinical Practice as outlined in the European Clinical Trials Directives 2001/20/EC and 2005/28/EC, the Medicines for Human Use (Clinical Trials) Regulations 2004 (SI 2004/1031) and any subsequent amendments of the clinical trial regulations, the Sponsor's SOPs, and other regulatory requirements as amended.

I agree to ensure that the confidential information contained in this document will not be used for any other purpose other than the evaluation or conduct of the clinical investigation without the prior written consent of the Sponsor.

Principal Investigator

Name:

Signature: ________________________

Date: ____________________________

2 TRIAL CONTACTS

**Chief Investigator**<br><br><br><br><br>**Co-lead Investigator**	Dr Lisa Willcocks<br>Cambridge University Hospital NHS Foundation Trust,<br>Hills Road, Cambridge CB2 0QQ, UK<br>lisa.willcocks@doctors.org.uk<br><br>Professor Megan Griffith<br>Renal Unit, 4th Floor Ham House, Hammersmith Hospital, Du Cane Road, London W12 OHS, UK<br>m.e.griffith@imperial.ac.uk
**Trial Coordinators**	Hira Hamza, Cambridge Clinical Trials Unit,<br>Addenbrookes Hospital, Cambridge CB2 0QQ, UK<br>01223 254684<br>hira.hamza@nhs.net
**Statistician**	Dr Wendi Qian, Cambridge Clinical Trials Unit, Addenbrooke's Hospital, Cambridge CB2 0QQ, UK<br>wq211@medschl.cam.ac.uk
**Health Economist**	Dr. Ed Wilson, University of Exeter
**Trials Pharmacist**	Dr Robyn Staples Central Pharmacy, Addenbrookes Hospital, Cambridge CB2 0QQ, UK<br>01223 217045<br>robyn.staples@nhs.net
**Co-Investigators**	Professor David Jayne, Experimental Medicine and Immunotherapeutics, University of Cambridge, Cambridge CB2 0QQ, UK<br><br>Professor Liz Lightstone, Renal Medicine, Imperial College London, London W12 OHS, UK<br><br>Professor Moin Saleem, Bristol Renal, Bristol Medical School, Bristol BS1 3NY, UK
**Patient Advisor**	Mr Alan Truswell

CONTENTS

1 PROTOCOL SIGNATURES 2

2 TRIAL CONTACTS 44

CONTENTS..55

3 ABBREVIATIONS 88

4 TRIAL SYNOPSIS...1010

5 TRIAL FLOW CHART...1414

6 INTRODUCTION 1515

6.1 Background.. 1515

6.2 Clinical data 1616

7 RATIONALE FOR TRIAL...1818

7.1 Clinical need 1818

7.2 Financial considerations 1818

8 TRIAL DESIGN...1919

8.1 Statement of design 1919

8.2 Number of centres 1919

8.3 Number of participants 1919

8.4 Participants trial duration 1919

8.5 Trial objectives 1919

8.6 Trial outcome measures... 2020

9 SELECTION AND WITHDRAWAL OF PARTICIPANTS...........................2121

9.1 Inclusion criteria 2121

9.2 Exclusion criteria 2121

9.3 Treatment assignment and randomisation number.............................. 2121

9.4 Method of blinding 2323

9.5 Participant Withdrawal Criteria 2323

10 TRIAL TREATMENT 2424

10.1 Treatment summary.. 2424

10.2 Open Label Phase (OLP)... 2626

10.3 Non IMPs 2626

10.4 Emergency Unblinding 2929

10.5 Accountability and Dispensing.. 2929

11 PROCEDURES AND ASSESSMENTS 2929

11.1 Participant Identification 2929

11.2 Consent.. 3030

11.3 Screening Evaluation... 3131

11.4 First and second dose of IMP 3232

11.5 Pre-remission trial visits – aligned with standard of care........................ 3232

11.6 Remission Visit .... 33~~33~~
11.7 Post-remission trial visits - aligned with standard of care .... 33~~33~~
11.8 Week 24 (pre 3rd IMP dose) assessment .... 34~~34~~
11.9 Third dose of treatment .... 34~~34~~
11.10 24 months assessment .... 34~~34~~
11.11 Relapse Visit .... 35~~35~~
11.12 Final trial visit .... 35~~35~~
11.13 Open label phase visits (See table 11.15) .... 35~~35~~
11.14 All Trial Visits except IMP infusions: .... 36~~36~~
11.15 Table of assessments for main phase of trial .... 37~~37~~
11.16 Table of assessments for Open Label Phase .... 39~~39~~
12 END OF TRIAL PARTICIPATION .... 41~~41~~
12.1 Trial Restrictions .... 41~~41~~
13 ASSESSMENT OF SAFETY .... 41~~41~~
13.1 Definitions .... 41~~41~~
13.2 Expected Adverse Reactions/Serious Adverse Reactions (AR /SARs) .... 42~~42~~
13.3 Expected Adverse Events/Serious Adverse Events (AE/SAE) .... 43~~43~~
13.4 Evaluation of AEs .... 44~~44~~
13.5 Reporting SAEs/SARs .... 45~~45~~
13.6 Reporting of SUSARs .... 45~~45~~
13.7 Pregnancy reporting .... 47~~47~~
14 EVALUATION OF RESULTS (Definitions and response/evaluation of outcome measures) .... 47~~47~~
14.1 Definitions of Remission and Relapse .... 47~~47~~
14.2 Evaluation Procedure .... 47~~47~~
15 STORAGE AND ANALYSIS OF SAMPLES .... 47~~47~~
16 STATISTICS .... 48~~48~~
16.1 Statistical Methods .... 48~~48~~
16.2 Number of Patients to be Enrolled .... 49~~49~~
16.3 Interim Analyses .... 49~~49~~
16.4 Economic Evaluation .... 50~~50~~
16.5 Definition of the End of the Trial .... 50~~50~~
17 DATA HANDLING AND RECORD KEEPING .... 51~~51~~
17.1 CRF .... 51~~51~~
17.2 Source data .... 51~~51~~
17.3 Data protection and participant confidentiality .... 52~~51~~
18 TRIAL COMMITTEES .... 52~~52~~
18.1 Trial Management Group (TMG) .... 52~~52~~

18.2 Independent Trial Steering Committee (TSC) .... 52~~52~~
18.3 Independent Data Monitoring Committee (IDMC)................................ 52~~52~~
19 ETHICAL AND REGULATORY CONSIDERATIONS ....53~~53~~
19.1 Consent.. 53~~53~~
19.2 Ethical Committee Review .... 53~~53~~
19.3 Regulatory compliance.. 53~~53~~
19.4 Protocol amendments .... 53~~53~~
19.5 Peer review.. 54~~54~~
19.6 Declaration of Helsinki and Good Clinical Practice .... 54~~54~~
19.7 GCP training.. 54~~54~~
20 SPONSORSHIP, FINANCIAL AND INSURANCE ....54~~54~~
21 MONITORING, AUDIT AND INSPECTION ....55~~55~~
22 PROTOCOL COMPLIANCE AND BREACHES OF GCP ....55~~55~~
23 PUBLICATIONS POLICY ....55~~55~~
24 REFERENCES ....56~~56~~
25 APPENDICES ....59~~59~~
25.1 Appendix 1 - Trial management / responsibilities................................ 59~~59~~
25.2 Appendix 2 – Authorisation of participating sites................................. 60~~60~~
25.3 Appendix 3 – Safety Reporting Flow Chart .... 61~~61~~
25.4 Appendix 4 – EQ-5D-5L Questionnaire .... 62~~62~~
.... 63~~63~~
25.5 Appendix 5 - Sample size output... 64~~64~~
25.6 Appendix 6 – Primary Outcome Measure Events .... 67~~67~~

3 ABBREVIATIONS

AAV	ANCA associated vasculitis
ACEi	Angiotensin Converting Enzyme Inhibitor
AE/AR	Adverse event/Adverse Reaction
AESI	Adverse Event of Special Interest
ARB	Angiotensin Receptor Blocker
CCTU	Cambridge Clinical Trials Unit
CI	Chief Investigator
CNI	Calcineurin Inhibitor
CR	Complete Remission
CRF	Case Report Form
CTA	Clinical Trial Authorisation
DSUR	Development Safety Update Report
eCRF	Electronic Case Report Form
EDoR	Expected Duration of Remission
ESKD	End Stage Kidney Disease
FR	Frequently relapsing
FSGS	Focal Segmental Glomerulosclerosis
GCP	Good Clinical Practice
GDPR	General Data Protection Regulation
GFR	Glomerular filtration rate
GP	General Practitioner
HIV	Human Immunodeficiency Virus
HRA	Health Research Authority
HTA	Health Technology Assessment
IB	Investigator’s Brochure
ICF	Informed Consent Form
IDMC	Independent Data Monitoring Committee
IMP	Investigational Medicinal Product
ISF	Investigator Site File
ISO	International Organisation for Standardisation
ITT	Intention To Treat
IUD	Intrauterine Device
IV	Intravenous
KDIGO	Kidney Disease Improving Global Outcomes
MCD	Minimal Change Disease
MCGN	Membranoproliferative glomerulonephritis
MesGN	Mesangiocapillary glomerulonephritis
MHRA	Medicines Healthcare products Regulatory Agency
MMF	Mycophenolate Mofetil
NHS	National Health Service
NephroS	National Study of Nephrotic Syndrome
NIHR	National Institute for Health Research
nIMP	Non Investigational Medicinal Product
NS	Nephrotic syndrome
OLP	Open Label Phase
PBRF	Probability-of-being-in-response function

PCR	Protein Creatinine Ratio
PI	Principal Investigator
PIS	Participant Information Sheet
PPR	Protocolised Prednisolone Regimen
PR	Partial Remission
QALY	Quality Adjusted Life Year
R&D	Research and Development
RaDaR	Registry of Rare Kidney Disease
RCT	Randomised controlled trial
REC	Research Ethics Committee
RRT	Renal Replacement Therapy
RSI	Reference Safety Information
SAE	Serious Adverse Event
SAR	Serious Adverse Reaction
SD	Steroid dependent
SLE	Systemic Lupus Erythematosus
SmPC	Summary of Product Characteristics
SPPR	Start of Protocolised Prednisolone Regimen
SUSAR	Suspected Unexpected Serious Adverse Reaction
TMF	Trial Master File
TMG	Trial Management Group
TSC	Trial Steering Committee
TURING	The Use of Rituximab In the treatment of Nephrotic Glomerulonephritis
UK	United Kingdom

4 TRIAL SYNOPSIS

**Title of clinical trial**	The Use of Rituximab In the treatment of Nephrotic Glomerulonephritis (TURING)
**Sponsor name**	Cambridge University Hospitals NHS Foundation Trust
**EudraCT number**	2018-004611-50
**Medical condition or disease under investigation**	Adult patients with de novo or relapsing nephrotic syndrome (NS) secondary to Minimal Change Disease (MCD) or Idiopathic Focal Segmental Glomerulosclerosis (FSGS).
**Purpose of clinical trial**	To assess the efficacy, safety, cost and cost-effectiveness of rituximab in treating de novo or relapsing NS in patients with MCD/FSGS.
**Primary objective**	To assess the efficacy of rituximab on the time to relapse of NS from partial/complete remission.
**Secondary objective (s)**	• To assess the effect of rituximab on NHS and societal resource use and hence cost.<br>• To assess the effect of rituximab on safety and on secondary measures of efficacy, such as achievement of remission of NS and the preservation of renal function.<br>• To assess the effect of rituximab on patient reported health status.
**Exploratory objective (s)**	• To explore the interaction between age of onset with the efficacy of rituximab.<br>To explore efficacy of delayed rituximab in nephrotic syndrome (data from OLP)
**Trial design**	• A randomised, two-arm (1:1 ratio), double blind, placebo controlled phase III trial<br>• An open label phase for participants randomised to placebo who achieve remission but relapse within 24 months of trial start.
**Trial outcome measures**	Time from partial/complete remission to relapse.
**Sample size**	130-150
**Summary of eligibility criteria**	***Inclusion Criteria:***<br>• Age 16 years or older.<br>• NS at Day 0 (Start of Protocolised Prednisolone regimen (SPPR)) (defined as serum albumin < 35g/l and protein creatinine ratio (PCR)

	>300mg/mmol) secondary to MCD/FSGS.<br>• De novo disease or relapsing disease in a patient previously steroid or calcineurin inhibitor (CNI) responsive.<br>• Latest biopsy (at any time) proven MCD/FSGS.<br>• Ability to provide written informed consent.<br>• Agreed to be enrolled in the National Registry of Rare Kidney Disease (RaDaR).<br><br>***Exclusion Criteria:***<br>• MCD or FSGS due to secondary causes, including obesity-driven hyperfiltration, remnant kidneys, malignancy of a type likely to be associated with MCD /FSGS and genetic polymorphisms known to be associated with nephrosis<br>• MCD/FSGS secondary to malignancy, including lymphoproliferative disorders<br>• Family history of MCD or FSGS in a first degree relative unless previously shown to be steroid responsive<br>• Previous rituximab within 18 months preceding Day 0 (Start of Protocolised Prednisolone regimen (SPPR)), or 12 months if there is evidence of B cell return in peripheral lymphocyte subsets<br>• Previous cyclophosphamide within 6 months preceding Day 0 (SPPR)<br>• Prednisolone daily dose equal to or greater than 60mg, with a course length of greater than 4 weeks, immediately prior to randomisation<br>• Evidence of current or past infection with Hepatitis B, C or HIV (unless appropriate prophylaxis is given and no replicating virus is detected).<br>• Positive serum pregnancy test (within 14 days prior to treatment with IMP in main trial and rituximab in OLP)<br>• Evidence of active severe infection<br>• Severe heart failure or severe, uncontrolled cardiac disease<br>• Pregnant or breast-feeding women<br>• Live vaccine administration in the four weeks prior to enrolment and while remaining on IMP treatment<br>• Previous/known hypersensitivity to prednisolone or IMP or to murine proteins (and any excipients as described in section 6.1 of the SmPC).

	• Co-enrolment in another clinical trial of an investigational medicinal product<br>• Any other reason which, in the opinion of the Principal Investigator (PI), renders the patient unsuitable for the trial.
**Investigational medicinal product (IMP) and dosage**	Rituximab<br>2 x 1g doses Intravenous (IV) 14 days apart, 1g at week 26
**Comparator product**	Placebo
**nIMPs**	Prednisolone and CNIs
**Route(s) of administration**	IV
**Maximum duration of treatment of a participant**	Main study phase: 26 weeks<br><br>Open Label Phase: additional 26 weeks post relapse
**Procedures: Screening & enrolment**	Demographics information, medical history, height and weight, blood pressure, haematology, biochemical analysis, immunoglobulins G and M, urinary PCR, serology for hepatitis B, C and HIV, concomitant medications, symptom check (AE review), serum pregnancy test (if applicable), 24-hour urine collection, prednisolone dosing, CNI dosing (if applicable), health status questionnaire (EQ5D5L), Additional Health Costs Questionnaire
**Procedures: IMP dosing visits**	Dose 1 within 4 weeks of Day 0 (SPPR)<br>Dose 2 given 2 weeks (- 1 week or + 2 weeks) after dose 1<br>Dose 3 26 (+/- 2 weeks) weeks after Day 0.<br>AE and concomitant medication reviews to take place at nearest preceding clinic visit.
**Procedures:**<br>**Pre remission** (every 4 weeks till remission)	Weight, blood pressure, haematology, biochemical analysis, urinary PCR, 24 hour urine collection for proteinuria (week 16 only), concomitant medications, adverse event (AE) assessments, health status questionnaire: EQ-5D-5L,Additional Health Costs Questionnaire (week 16 only), prednisolone dosing, CNI dosing (weeks 4, 8 and 12 only if applicable),
**Procedures: Post remission** (every 4 weeks post remission for 12 weeks then 3 monthly until relapse or end of trial)	Weight, blood pressure, haematology, biochemical analysis, urinary PCR, 24 hour urine collection for proteinuria (month 24 only), concomitant medications, AE assessments, urine protein stick testing (only if CR achieved), health status questionnaire: EQ-5D-5L (3 monthly only), Additional

	Health Costs questionnaire (6 monthly only).<br>IMP dosing (week 26 only), immunoglobulins G and M (week 26 then 6 monthly) and serum/urine pregnancy test (if applicable; week 26 only).
**Procedures: Relapse** (within 2 weeks after relapse indicated)	Weight, blood pressure, Haematology, Biochemical analysis, Urinary PCR, 24 hour urine collection for proteinuria, concomitant medications, AE assessments, Health status questionnaire: EQ-5D-5L, Prednisolone compliance/dosing
**Procedures: End of Trial**	End of Trial visits are defined as per section 11.9.
**Procedures for safety monitoring during trial**	Serious adverse events (SAEs) and other adverse events related to steroids or rituximab will be regularly reviewed by Trial Management Group (TMG) and Independent Data Monitoring Committee (IDMC).
**Criteria for withdrawal of participants**	Failure to achieve complete or partial remission at 16 weeks will result in withdrawal from the trial with no further trial visits

5 TRIAL FLOW CHART

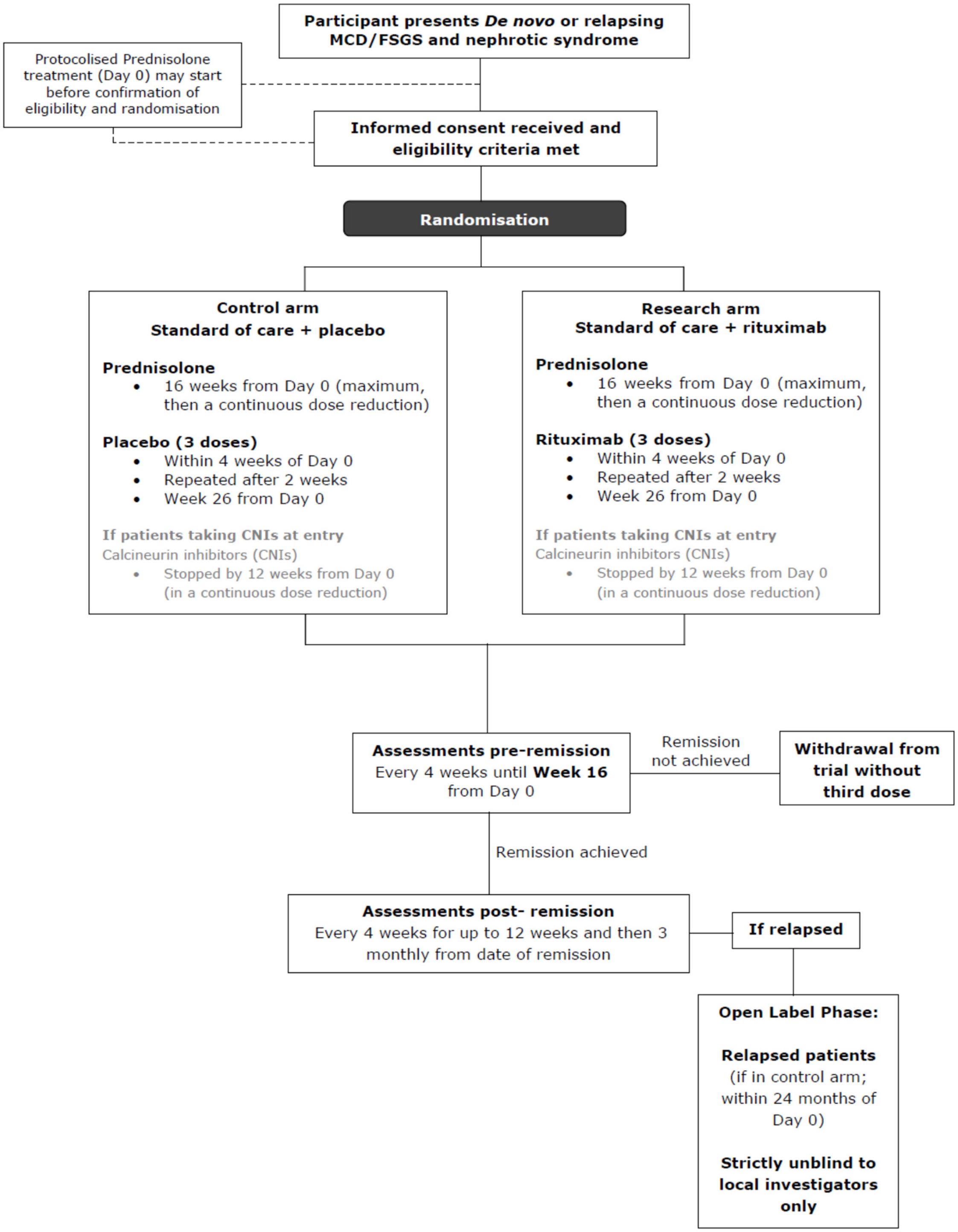

6 INTRODUCTION

6.1 Background

Minimal Change Disease (MCD) and Focal Segmental Glomerulosclerosis (FSGS) are auto-immune renal diseases that present with a common clinical phenotype, the nephrotic syndrome (NS), characterised by heavy proteinuria and debilitating oedema. When nephrotic, patients require frequent hospital admissions; infections and venous thrombo-embolism are common and can be fatal. With treatment, patients with MCD usually have preserved renal function, however, those with FSGS frequently still progress to end stage kidney disease (ESKD), requiring renal replacement therapy (RRT). Patients with FSGS and NS have five year renal survival rates (without dialysis or transplantation) of 60-90% and ten year renal survival rates of 30-55%[1,2]. 10 year survival improves to 80% if remission from proteinuria is achieved[3,4].

Primary MCD and FSGS are rare, affecting about 10/million population/year. Together they are now responsible for 50% of adult idiopathic NS presentations[5]. MCD and FSGS are historically described as two separate disease entities. However, emerging data indicate that they are part of a disease continuum[6]. Both conditions are characterised by electron-microscopic evidence within the glomerulus of diffuse effacement of podocyte foot processes, resulting in a severe functional defect in selective permeability. In FSGS, there is additional focal (i.e. in some areas of the kidney cortex) and segmental (segments of the glomerulus are affected) fibrosis and occlusion of the glomerular capillaries[8]. It is notable that, in FSGS, glomeruli not displaying segmental lesions demonstrate the classical features of MCD at electron microscopy.

RELATIVE FREQUENCY (%) OF PRIMARY GLOMERULAR DISEASE UNDERLYING NEPHROTIC SYNDROME

Disease	1960s and 70s Adults < 60 years	1960s and 70s Adults > 60 years	1990s to the present
Focal Segmental Glomerulosclerosis (FSGS)	15	2	35
Minimal Change Disease (MCD)	20	20	15
Other glomerular disease	65	78	50

Adapted from Hull and Goldsmith[5]

The aetiology of primary MCD and FSGS has not been completely delineated. However, there is strong evidence of a circulating factor, or factors, in MCD and FSGS[7]. Definitive evidence of a circulating factor is provided by the recurrence of NS following kidney transplantation in 20-30% of patients with FSGS[6]. It is likely that circulating factors are released by immune cells, particularly at the time of intercurrent infection. Several other strands of evidence support the view that MCD and FSGS have an immune aetiology. Both diseases respond to immunosuppression with glucocorticoids. There is evidence for T cell involvement: in children with idiopathic NS regulatory T-cell numbers were reduced and cytokine profiles altered during relapse[8, 9]. In a rat model of FSGS, transfer or induction of regulatory T-cells results in disease attenuation[10]. B cells have also been implicated: increased B cell numbers were present in the kidneys of children with FSGS compared with mesangiocapilliary glomerular nephritis MCGN (another kidney disease characterised by proteinuria) [11]. In a murine model, transfer

of glomerular antigen-specific B cells induced glomerular injury and proteinuria. This effect was mediated by IL-4, as transfer of IL-4-deficient B cells did not induce proteinuria. Overexpression of IL-4 in mice was sufficient to induce kidney injury and proteinuria and could be attenuated by JAK kinase inhibitors. In kidney biopsies of patients with MCD, STAT6 activation (induced by IL-4) was increased, suggesting IL-4 exposure in these patients. These data also suggest that the role of B cells in NS could be mediated by cytokines[12].

Thus existing evidence supports the hypothesis that MCD and FSGS represent manifestations on a disease continuum, and are likely to have an auto-immune aetiology. Given that existing treatments overlap, this trial addresses MCD and FSGS as a single disease entity.

6.2 Clinical data

There is a paucity of evidence to support existing treatments for MCD/FSGS. The Kidney Disease Improving Global Outcomes (KDIGO) recommendations concludes that no evidence graded higher then 'C' exists for the treatment of these diseases in adults[13,14,15].

6.2.1 Glucocorticoids

Glucocorticoids are the mainstay of treatment in MCD/FSGS, with MCD typically responding faster than FSGS. Response rates are higher in MCD at 90%, compared to those in FSGS at 40-80%. In steroid responsive patients with both diseases, recurrent relapses occur in 75% when the steroid dose is reduced or withdrawn. These frequently relapsing (FR) or steroid dependent (SD) patients accrue a high steroid exposure over time, with weight gain, diabetes, infection and osteoporosis. In patients with FSGS, renal function deteriorates if long term remission of proteinuria cannot be achieved. In one retrospective analysis (n=197), 23% (n=45) progressed to dialysis after a median follow-up of 1.8 years from the time of renal biopsy diagnosis[16]. Achieving remission is very important for avoiding ESKD – in a cohort study of 281 patients with nephrotic FSGS, achieving at least partial remission was associated with markedly improved renal survival with a hazard ratio of 0.48 (95% confidence interval 0.24-0.96:p=0.04)[17]. ESKD is associated with poor quality of life and a risk of death exceeding most cancers[18] and is expensive for the NHS[19].

6.2.2 Other immunosuppression: cyclophosphamide calcineurin inhibitors (CNIs) and mycophenolate mofetil (MMF)

Due to the morbidity of prolonged/repeated course of steroids, steroid sparing agents are regularly used in MCD/FSGS. Cyclophosphamide is thought to be most effective at maintaining remission in SD/FR MCD/FSGS[20], but the severe cumulative toxicity precludes long term use. CNIs are also effective at maintaining remission. In an open-label prospective cohort study of 26 adults with FR or SD NS, tacrolimus was as effective as cyclophosphamide at inducing and maintaining remission at 2 years[21]. However, relapse rates are high on stopping therapy, so repeated prolonged courses are often required for many years. CNIs themselves are nephrotoxic, and patients require long term follow up with close monitoring of levels in the clinic for the duration of their therapy. In an open-label prospective cohort study, 12 adult patients with SD MCD were treated with tacrolimus therapy[22].The authors reported complete remission in 10 patients and partial remission in an additional patient. During a mean follow-up period of 24 months, 50% experienced relapse. In a small trial in children with steroid resistant NS, Choudhry and colleagues randomised 41 patients to tacrolimus or cyclosporine[23]. Remission occurred in 85.7% and 80% of patients treated with tacrolimus and cyclosporine respectively, although relapses were more common in the cyclosporine group.

Mycophenolate is also used in clinical practice as a steroid sparing agent for patients with SD/FR MCD/FSGS. However there is little evidence to support this - in a Randomised Controlled Trial (RCT)[24] by Gipson et al, mycophenolate was combined with high dose steroids in the form of dexamethasone. In this trial of 138 children and young adults with relapsing FSGS, cyclosporine was compared to dexamethasone and MMF and showed no significant difference in remission rates between trial arms (cyclosporine 46%, mycophenolate and dexamethasone 33%). The efficacy of using MMF alone instead of steroids is not clear.

6.2.3 Rituximab

Rituximab is a monoclonal antibody that targets and depletes B cells via CD20. It is a licensed treatment for other auto-immune diseases, rheumatoid arthritis and antineutrophil cytoplasmic antibody (ANCA) associated vasculitis, where it has an excellent safety profile. In a recent pooled analysis of 3,194 patients with rheumatoid arthritis exposed to 11,962 patient years of rituximab[25], serious infections occurred at a rate of 3.94 per 100 patient years (comparable to both methotrexate and placebo). Importantly, the risk of serious infections was stable over time, even with multiple courses of treatment. Opportunistic infections were very rare, and only two cases of pulmonary tuberculosis occurred. Rates of viral infections, including herpes zoster reactivation (9/1000 patient years), were low. Rituximab may therefore be considerably safer than existing treatments for MCD/FSGS.

As the pathogenesis of MCD/FSGS is not fully understood, the mechanism of action of rituximab is debated. Rituximab may exert its effect in MCD/FSGS through targeting B cells,[26] but there is evidence that rituximab may prevent podocyte apoptosis by binding directly to podocyte sphingomyelin phosphodiesterase acid-like 3b (SMPDL-3b) and stabilising the cytoskeleton although the clinical impact of this observation is uncertain[27].

Randomised trial evidence to support the use of rituximab in MCD/FSGS is limited to paediatric studies. Here, NS is almost always due to MCD/FSGS, and kidney biopsy is rarely done. Instead, patients are treated on the basis of their clinical presentation with SD/FR NS. Ravani et al randomised 54 children with SDNS in an open-label non-inferiority design to receive rituximab and reduced dose steroid and cyclosporine, or standard steroid and cyclosporine doses[28]. Proteinuria was lower after 3 months in the rituximab arm, and relapses occurred in only 18.5% of the rituximab arm compared with 48% in the control arm (p=0.029). Notably, the probability of being drug free at 3 months were 62.9% and 3.7%, respectively (p<0.001). In a double-blind randomised trial, Iijima and colleagues randomly assigned 48 children with SDNS to rituximab or placebo. Patients in the rituximab arm demonstrated a 50% increase in the median time to relapse (p-0.0001) [29]. In a further non-inferiority open-label randomised trial[30], Ravani et al assigned 30 children with SDNS to receive either continued steroid therapy or a single IV infusion of rituximab. After 3 months, proteinuria was 42% lower with rituximab than control. The median time to relapse with rituximab was 18 months, whereas 14/15 control patients relapsed within 6 months. Several observational cohort studies in children[31-35] support the findings from the above randomised trials.

Evidence to support the use of rituximab in adults with MCD/FSGS is more limited, but several observational studies have reported encouraging results. In a series of 17 adult patients with SD or FR MCD treated with rituximab, 11 patients remained relapse free (median follow-up 27 months), of whom 9 discontinued all immunosuppression[36]. In another series of 41 adult rituximab treated patients with SD or FR MCD, complete remission was achieved in 25 and

partial remission in a further 7 patients. After a median follow-up of 39 months, 18 responders had experienced a relapse (mean time to relapse 18 (3-36) months). Thirteen of 17 patients retreated with rituximab again achieved remission[37]. A prospective study of 25 adult patients with SD or FR MCD identified only 4 relapses within 12 months of rituximab treatment, and a reduction in the maintenance steroid dose in most patients[38,39]. Papakrivopoulou et al reported a reduction in the frequency of relapse from 2.9 to 0.4 per patient year after treating 15 adult patients with SD or FR MCD with rituximab[40]. In a series of 8 SD or FR FSGS adult cases treated with rituximab, 5 patients demonstrated no response. The three responders received higher doses of rituximab, suggesting that non-responders may have been under-treated[41]. Ruggenenti describes a cohort of 30 patients (10 children and 20 adults) with SD/FR NS secondary to MCD/FSGS and mesangiocapillary glomerulonephritis (MesGN). Nineteen patients had MCD, 8 FSGS and 3 MesGN. Following treatment with rituximab, the number of relapses fell from 88 in the year prior to rituximab to 22 during 1 year of follow up $p<0.001$[42]. This reduction in relapse risk was significant when children and adults, and MCD/MesGN and FSGS patients were considered separately.

7 RATIONALE FOR TRIAL

7.1 Clinical need

MCD/FSGS are rare auto-immune kidney diseases. Untreated, they result in considerable morbidity including kidney failure and death. Current treatments have serious limitations: whilst glucocorticoids are effective in the majority of patients, repeated courses are usually required, and the side effect burden of cumulative steroid exposure itself carries extensive morbidity and mortality for patients. In other autoimmune diseases where immunosuppression is required, steroid sparing regimens (including the use of biologics) are now the norm. In MCD/FSGS there is an urgent need for such steroid sparing regimes, that are both effective and safe, and do not adversely affect kidney function. Rituximab is the most promising candidate treatment. Indeed, it is currently used where other treatments have failed, or where the risk of toxicity from existing treatments is deemed unacceptably high. However, such use lacks supporting evidence from robust interventional trials, leading the KDIGO expert group to identify trials with rituximab in adults with MCD/FSGS as a key research priority. Given pressures on healthcare resources, studies assessing both the efficacy and cost-effectiveness of rituximab as treatment for MCD/FSGS within the context of the NHS are urgently needed.

7.2 Financial considerations

MCD/FSGS often affects patients younger than 65 years[5], when they are economically active. The symptoms of NS and the requirement for hospital-based treatment result in time off work and loss of income. Complications arising from the disease or its treatment, such as serious infections, may require further hospital admissions. Loss of kidney function can result in ESKD requiring dialysis at great cost. Indeed, glomerulonephritides account for 18.9% of patients requiring RRT, and this is higher in those under 65 years of age (21.3%). Although MCD/FSGS make up only a proportion of this number, treatments that delay the onset of ESKD should result in a significant cost saving. Currently, patients are treated with multiple courses of a variety of different immunosuppressants over many years or even decades in an effort to reduce steroid exposure. Even if these treatments are successful in a steroid sparing capacity, they have significant toxicity themselves - oral cyclophosphamide in particular carries a high

risk of leucopenia and sepsis. There is a clear need for alternative effective treatment such as rituximab for MCD/FSGS. Given that rituximab is now off patent and biosimilar preparations are available, the cost of rituximab is likely to reduce further. TURING is therefore designed to assess the efficacy, safety, cost and cost effectiveness of rituximab at inducing sustained remission in patients with NS caused by primary MCD/FSGS.

8 TRIAL DESIGN

8.1 Statement of design

This is a randomised, two-arm (1:1 ratio), double blind, placebo controlled phase III trial to assess the efficacy, safety, cost and cost-effectiveness of rituximab in treating *de novo* or relapsing NS in patients with MCD/FSGS.

In addition to the main phase of the trial, the participating Renal Units will also offer the Open Label Phase to the participants on the placebo arm who relapse after achievement of partial or complete remission.

8.2 Number of centres

We plan to include approximately 40 UK Renal Units within this trial; however we may increase the number of centres if required in order to recruit the required number of participants.

8.3 Number of participants

We plan to randomise approximately 130-150 participants in this trial. Please see Section 16.2 Number of patients to be enrolled for details.

8.4 Participants trial duration

Participants will have a minimum of 24 months follow up from Day 0 (Start of Protocolised Prednisolone Regimen) unless a partial remission is not achieved at week 16 or the primary outcome measure event is observed earlier (see section 11.6 end of trial visit). Participants who achieve the primary endpoint and subsequently enter the Open Label Phase (OLP) will have a minimum of 26 weeks follow up in the OLP.

All participants in whom the primary outcome measure event is not observed will remain in the trial, in follow-up for a minimum of 24 months.

All participants will return to their standard of care after end of trial participation.

8.5 Trial objectives

8.5.1 Primary objective:

- Assess the efficacy of rituximab on the time from partial / complete remission to relapse of NS.

8.5.2 *Secondary objectives*

- Assess the effect of rituximab on NHS and societal resource use and hence cost.
- Assess the effect of rituximab on safety and on secondary measures of efficacy, such as achievement of partial and complete remission of NS and the preservation of renal function.
- Assess the effect of rituximab on health status of participant.

8.5.3 *Exploratory objective*

- To explore the interaction between age of onset with the efficacy of rituximab.
- To explore efficacy of delayed rituximab in nephrotic syndrome (data from OLP)

8.6 **Trial outcome measures**

8.6.1 *Primary outcome measure*

Primary outcome measure: Time from partial or complete remission (whichever documented first) to relapse of NS as defined below (Appendix 6):

- COMPLETE REMISSION (CR): PCR<50mg/mmol (only one result required).

- PARTIAL REMISSION (PR): Reduction in Urinary PCR≥ 50% from Day 0 - Start of Protocolised Prednisolone Regimen (SPPR) AND PCR ≥50mg/mmol and ≤300mg/mmol on two consecutive results (performed within 14 days of each other). The date of first assessment is the date of partial remission.

- RELAPSE: recurrent NS as defined by albumin < 35 g/l, PCR >300mg/mmol and an increase in 100% over the nadir value during remission on two consecutive results (performed within 14 days of each other). The date of first assessment is the date of relapse. For patients with an albumin ≥35g/l prior to relapse, only one PCR result is required (i.e. PCR > 300mg/mmol that has risen by 100% over the nadir value and an albumin < 35g/L).

8.6.2 *Secondary outcome measures*

Secondary assessments will include an evaluation of the effect of rituximab on:

- Proportion of patients achieving partial or complete remission
- Time to partial or complete remission from Day 0 (SPPR)
- Time to treatment failure (not achieving remission by week 16 or relapse) from randomisation
- Serious AEs
- AE of Special Interest, including infection and steroid-associated side effects
- Change in urinary PCR/24 hour proteinuria
- Change in serum albumin
- Kidney function as assessed by the change in Glomerular filtration rate (GFR) from Day 0 - Start of Protocolised Prednisolone Regimen (SPPR) to 24 months and to trial end
- Health Status (EQ-5D-5L)
- NHS resource use, out of pocket expenditure and morbidity-related lost productivity, (Additional Health Costs)

9 SELECTION AND WITHDRAWAL OF PARTICIPANTS

9.1 Inclusion criteria

To be included in the trial the participant must:

- Have given written informed consent to participate
- Age 16 years or older at the time of consent
- Have confirmed NS at Day 0 (Start of Protocolised Prednisolone regimen (SPPR)) (defined as serum albumin < 35g/l and PCR>300mg/mmol) secondary to MCD/FSGS with,
  - *De novo* disease, or;
  - Relapsing disease in a patient previously steroid or CNI responsive (this is defined as patients who have achieved complete or partial remission from their NS in response to treatment with steroid or CNI).
- Biopsy proven MCD or FSGS (from medical records)
- Agreed to be enrolled in the National Registry of Rare Renal Diseases (RaDaR)

9.2 Exclusion criteria

The presence of any of the following will preclude participant inclusion:

- MCD or FSGS due to secondary causes, including obesity-driven hyperfiltration, remnant kidneys, malignancy of a type likely to be associated with MCD /FSGS and genetic polymorphisms known to be associated with nephrosis
- MCD/FSGS secondary to malignancy, including lymphoproliferative disorders
- Family history of MCD or FSGS in a first degree relative unless previously shown to be steroid responsive
- Previous rituximab within 18 months preceding Day 0 (SPPR), or 12 months if there is evidence of B cell return in peripheral lymphocyte subsets
- Previous cyclophosphamide within 6 months preceding Day 0 (SPPR)
- Prednisolone daily dose equal to or greater than 60mg, with a course length of greater than 4 weeks, immediately prior to randomisation
- Evidence of current or past infection with Hepatitis B, C or HIV (unless appropriate prophylaxis is given and no replicating virus is detected).
- Positive serum pregnancy test (within 14 days prior to treatment with IMP in main trial and rituximab in OLP)
- Evidence of active severe infection
- Severe heart failure or severe, uncontrolled cardiac disease
- Pregnant or breast-feeding women
- Live vaccine administration in the four weeks prior to enrolment and while remaining on IMP treatment
- Previous/known hypersensitivity to prednisolone or IMP or to murine proteins (and any excipients as described in section 6.1 of the SmPC).
- Co-enrolment in another clinical trial of an investigational medicinal product
- Any other reason which, in the opinion of the Principal Investigator (PI), renders the patient unsuitable for the trial.

9.3 Treatment assignment and randomisation number

9.3.1 <u>Method of randomisation</u>

Eligible participants will be randomly assigned to either the standard of care + placebo

(control arm) or the standard of care + rituximab (research arm) in a 1:1 ratio using the stratified random block method. Stratification factors are:

- Patient type
- Disease type
- Prednisolone regimen

9.3.2 Procedure of randomisation

A web-based central randomisation system will allocate each participant a Trial Subject ID and treatment kit code.

At site initiation meetings, the trial co-ordinator will arrange for the suitably trained and delegated members of trial team to have access to a unique system username and password, which will allow them to access the central randomisation system. The trial co-ordinator will also train site staff in how to access and use the randomisation system throughout the trial.

In order to enrol a patient, data will be entered onto the central randomisation system via an internet-based interface. The following data will be required in order to randomise a patient:

- Confirmation that the patient satisfies all the eligibility criteria
- Patient's date of birth
- Patient type (*De novo* disease vs. relapsing disease)
- Disease type (MCD vs. FSGS)
- Prednisolone regime (Regimen 1 vs Regimen 2)

A unique treatment kit code will be allocated and a unique trial Subject ID will be assigned by the randomisation system for the participant. The treatment kit code will be used by the unblinded pharmacy or other authorised site personnel to determine the treatment arm the participant has been randomised to.

A paper-based randomisation system will also be in place as a back-up if the online randomisation system in unavailable. See section 9.3.2.1 for further details.

9.3.2.1 Paper randomisation

If the web-based randomisation system is unavailable to randomise an eligible participant at any participating site, the site team can contact the trial coordinators based in Cambridge to a request paper randomisation be carried out.

In order to randomise a participant, the following data will be required:

- Confirmation that the patient satisfies all the eligibility criteria
- Patient's date of birth
- Patient type (*De novo* disease vs. relapsing disease)
- Disease type (MCD vs. FSGS)
- Prednisolone regime (Regimen 1 vs Regimen 2)

A unique treatment kit code will be allocated and a unique trial Subject ID will be assigned by the trial coordinator via paper-based randomisation system. The email containing details of Subject ID, kitcode and site details will be sent to site team, site pharmacy, CI and co-lead.

The treatment kit code will be used by the unblinded pharmacy or other authorised site personnel to determine the treatment arm the participant has been randomised to.

The data used to randomise participants by this method will be retrospectively added onto the web-based system when it is available again by authorised delegated individuals and site teams will be made aware they can access this data via the web.

9.4 Method of blinding

During the double-blind phase of the trial, participants and research teams (excluding the site pharmacy or other authorised site personnel) will be blinded to the treatment group until participants reach the primary end point of relapse or end of trial. At the point of relapse, participants and the local Principal Investigator will be unblinded to assess whether they are eligible to progress into the Open Label Phase. If the trial is stopped earlier (e.g. for safety reasons), at the recommendation of the Independent DMC and the approval of the TSC, all participants would be unblinded. Unblinding could also occur at the request of the local investigator if participants have failed to achieve at least partial remission at 16 weeks, and if unblinding is necessary for patient safety when further treatment decisions are made. The physical appearance of the IMPs is matched and the IMPs will be presented in identical packaging at the point of release by site pharmacies.

9.5 Participant Withdrawal Criteria

Participants who are withdrawn or choose to withdraw from the trial will not be replaced.

9.5.1 Withdrawal from protocol treatment

A participant may withdraw, or be withdrawn, from the trial treatment for the following reasons:

- Participants who do not achieve partial remission by 16 weeks after Day 0 (SPPR). The treating clinician may request unblinding of withdrawn participants if required for safety consideration of future treatment – this unblinding will be performed at local site level only
- Participants who enter the trial and remit and relapse before receiving the first dose of trial IMP. In this case the participant can be withdrawn from the trial and re-screened as a new participant. It is recommended the clinician chooses Regimen 1 steroid taper for this participant when re-entering them into the trial
- Progressed or relapsed disease whilst on therapy
- Unacceptable toxicity
- Participant choice
- Addition of other immunosuppressants for treatment of MCD/FSGS
- Any alterations in the participant’s condition which justifies the discontinuation of treatment in the investigator’s opinion.
- Failure to adhere to infusion visit schedule. With ongoing consent, patients should remain in the trial and be followed up according to the protocol visit schedules. Samples and data collected up to the time of withdrawal will be included in the data reported for the trial.

9.5.2 Duration of safety reporting

Safety reporting of SARs/SUSARs is required for trial participants withdrawn from trial treatment (including those withdrawn from trial treatment eg for non-response) for a minimum of 12 months following the last dose of IMP. Rituximab causes B cell depletion, with B cell recovery beginning within 6 months. By 12 months after treatment, B cell counts have generally returned to normal (Truxima SmPC).

If a trial participant has withdrawn his/her consent all trial-specific tests and assessments should be stopped from the point of withdrawal, including safety reporting.

They should be followed up by their treating clinicians until the event resolution but no new data should be collected from them for the trial purposes

9.5.3 Withdrawal of consent

Patients may withdraw their consent to participate in the trial at any time. If the patient explicitly states their wish not to contribute further data to the trial, the investigator should inform the co-ordinating centre in writing and the withdrawal of consent should be documented by the investigator in the patient's case report forms (CRF). However, data collected up to the time of consent withdrawal will be included in the data reported for the trial.

10 TRIAL TREATMENT

10.1 Treatment summary

For the purpose of this trial, the following are considered as IMPs:

Double-blind phase
- Rituximab
- Placebo to match Rituximab (sodium chloride 0.9%)

Open label phase
- Rituximab

10.1.1 Supply

All IMPs and Non-IMPs in this trial will utilize standard commercial stock. Biosimilar rituximab preparations are permitted.

10.1.2 Name and description of Rituximab

Rituximab is a monoclonal antibody that targets CD20, a protein found on the surface of B lymphocytes. Once rituximab has bound CD20, it causes depletion of B cells circulating in the blood by three mechanisms:
- Antibody dependent cellular cytotoxicity
- Complement dependent cytotoxicity
- Apoptosis

Rituximab was approved for medical use in 1997. It is on the World Health Organisation's List of Essential Medicines[43], the most important medications needed in a basic health care system. It is licensed for use in refractory rheumatoid arthritis in both USA and Europe with extensive RCT and registry data supporting its efficacy and safety. There are also data

supporting the use of rituximab to treat other autoimmune diseases affecting the kidneys including lupus nephritis[44,45] and ANCA associated vasculitis (AAV)[46,47]. NHS England currently fund rituximab treatment for patients with refractory systemic lupus erythematosus (SLE) and AAV, hence the UK nephrologists are familiar with using the drug. In these diseases, rituximab is given as 2 doses of 1g two weeks apart inducing B cell depletion for 6-12 months. Rituximab is also being used as a maintenance agent, with repeat dosing every 6 months shown to be safe and effective[48,49].

10.1.3 Legal status

Rituximab is licensed for the treatment of a number of conditions, including B cell lymphoma, Rheumatoid Arthritis and ANCA Vasculitis. It has previously been studied in paediatric populations. Within this trial, the IMP is being used in a new population (adults with de novo or relapsing nephrotic syndrome secondary to MCD/FSGS).

The trial is being carried out under a Clinical Trial Authorisation. The drug is therefore only to be used by the named investigators, for the participants specified in this protocol, and within the trial.

10.1.4 Packing and labelling

IMP infusions will be supplied with a blinded label at the point of dispensing.

10.1.5 Storage conditions

Rituximab will be stored in accordance with the requirement of its respective SmPC.

10.1.6 Maximum duration of treatment of a participant

Patients will receive rituximab treatment at the start of the trial for up to 26 (+/- 2) weeks from Day 0 (SPPR). Those patients in the control arm who consent to the Open Label Phase of the trial will receive treatment with the intervention described for the rituximab arm in the main trial – i.e. for a further 26 (+/- 2) weeks.

10.1.7 Dose

The rituximab dose used is 1g for all treatments. Rituximab 1g will be administered within 4 weeks of Day 0 (SPPR) and is then repeated after 2 weeks (- 1 week or + 2 weeks). Provided the participant achieves partial or complete remission by week 16, a further 1g is administered at 26 weeks (+/- 2 weeks) from Day 0 (SPPR).

10.1.8 Administration

Rituximab will be administered intravenously as per SmPC instructions. To reduce the risk of infusion reactions, pre-medication will be given prior to each dose as per local practice – the recommended regime is methylprednisolone 100mg intravenously, chlorpheniramine 10mg intravenously and paracetamol 1g orally.

10.1.9 Known drug reactions

Known drug reactions for rituximab are summarised in Section 4.8 of the SmPC for Rituximab approved by the Medicines and Healthcare products Regulatory Agency (MHRA) for use in this trial. Currently, there is limited data on possible drug interactions with Rituximab.

10.1.10 Dose modifications

Dose modifications to IMP treatment are not permitted. Treatment with rituximab will not

proceed if a participant suffers an Adverse Reaction to the drug (at the discretion of the treating clinician).

10.1.11 Placebo

Patients randomised to placebo will receive a NaCl 0.9% infusion to match the rituximab infusion, administered at the same time points and prepared by unblinded site pharmacy or other authorised site personnel. Patients in the placebo arm will also receive pre-medication as per local practice, the recommended regime is methylprednisolone 100mg intravenously, chlorpheniramine 10mg intravenously and paracetamol 1g orally.

10.2 **Open Label Phase (OLP)**

Patients randomised to placebo who achieve partial/complete remission by week 16, followed by disease relapse (the primary endpoint event) within 24 months of Day 0 (SPPR) will be invited to participate in the Open Label Phase (OLP) of the trial.

Participants who fail to remit by week 16 will be ineligible for the OLP, as patients who are not steroid responsive are unlikely to respond to rituximab therapy. There is limited data in this group, but a single open label RCT in children whose nephrotic syndrome was unresponsive to steroids and CNIs did not show any benefit of rituximab[50]. Data arising from the OLP will not contribute to either the efficacy or safety assessments in the main phase of the trial but will allow exploratory observational analyses, including a comparison of time in remission combining both standard of care or placebo treatment and rituximab treatment.

Those participants in the control arm who consent to the OLP will receive treatment with the intervention described for the rituximab arm in the main trial – i.e. receive rituximab 1g within 4 weeks of relapse and restarting treatment prednisolone, after 2 weeks (+/-7 days) and, if they continue in remission, after 26 weeks (+/- 2 weeks) of restarting treatment dose prednisolone for relapse. Thus participants will attend 3 additional visits on top of standard care for the rituximab infusions. SAE reviews and concomitant medication reviews will be conducted during these visits.

All participants that enter the OLP will recommence protocolised prednisolone therapy as in the main trial (with the regimen at the discretion of the local investigator).

10.3 **Non IMPs**

10.3.1 Prednisolone

Prednisolone is a glucocorticoid used very widely used to treat a multitude of conditions. Side effects are also well described, including increased risk of infection and diabetes, osteoporosis, weight gain, fluid and salt retention, gastritis, skin thinning, poor wound healing, weight gain and a moon face.

In the trial, patients will be prescribed daily oral prednisolone to take at home on a reducing schedule according to the trial protocol. The Protocolised Prednisolone Regimen (PPR) will be started at Day 0, which may be combined with the screening visit if it is clinically appropriate to start glucocorticoid therapy at this time point.

Prednisolone will be dosed as follows:

REGIMEN 1: is based on National and International guidelines for the treatment of nephrotic FSGS. Prednisolone 1mg/kg/day (max 60mg) started (or continued) at Day 0, and continued daily for up to 16 weeks. A maximum of two weeks after obtaining partial or complete remission of proteinuria the prednisolone dose will be reduced to:

40mg daily for 2 weeks
30mg daily for 2 weeks
25mg daily for 2 weeks
20mg daily for 2 weeks
15mg daily for 2 weeks
10mg daily for 2 weeks

Thereafter, reduction will be 5mg daily for 2 weeks, 5mg alternate days for 2 weeks then stop or reduction to a maintenance dose of <10mg daily for patients on long term prednisolone (with further reduction determined by the local investigator as clinically appropriate). This regimen is recommended for patients with a first presentation of the nephrotic syndrome.

*REGIMEN 2***:** In patients at risk of steroid toxicity or who have previously responded rapidly to prednisolone, local investigators can choose a lower dose regime. Patients will start treatment with prednisolone 1mg/kg/day (max 60mg) continued daily for up to 16 weeks, and a maximum of two weeks after obtaining partial or complete remission of proteinuria will reduce the dose to:

30mg daily for 2 weeks
20mg daily for 2 weeks
15mg daily for 2 weeks
10mg daily for 2 weeks

Thereafter, reduction will be 5mg daily for 2 weeks, 5mg alternate days for 2 weeks then stop or reduction to a maintenance dose of <10mg daily for patients on long term prednisolone (with further reduction determined by the local investigator as clinically appropriate).

Local investigators will select the appropriate prednisolone regime prior to randomisation based on previous toxicity and steroid responsiveness. The regimen selection will be stratified at randomisation. Variations in prednisolone doses of up to one third of the selected regime will be permitted and will not be considered protocol violations. However, any increase in prednisolone dose should be discussed with the trial management team before being prescribed.

Tapering steroid diaries will be provided for participant’s use - completion of these is not mandatory. The diaries should be reviewed at trial visits to aid CRF completion.

10.3.2 Calcineurin inhibitors (CNIs)

Tacrolimus and cyclosporine are immunosuppressive medications widely used after organ transplantation. They are also used to treat primary glomerulonephritis, and patients with SD/FR MCD/FSGS are often treated with these drugs. These drugs will be permitted in patients enrolled into the trial.

Following randomisation, the daily CNI dose will be reduced as follows:

25% dose reduction within 48 hours of randomisation
50% dose reduction (from dose at randomisation) at week 4 (+/-2 weeks) (from Day 0 SPPR)
75% dose reduction (from dose at randomisation) at week 8 (+/-2 weeks) from Day 0 SPPR)

The CNI will then be stopped at week 12 (+/-2 weeks) from Day 0 SPPR.

Variations in the daily CNI dose of up to 25% at each of these time points will be permitted and will not be considered protocol violations.

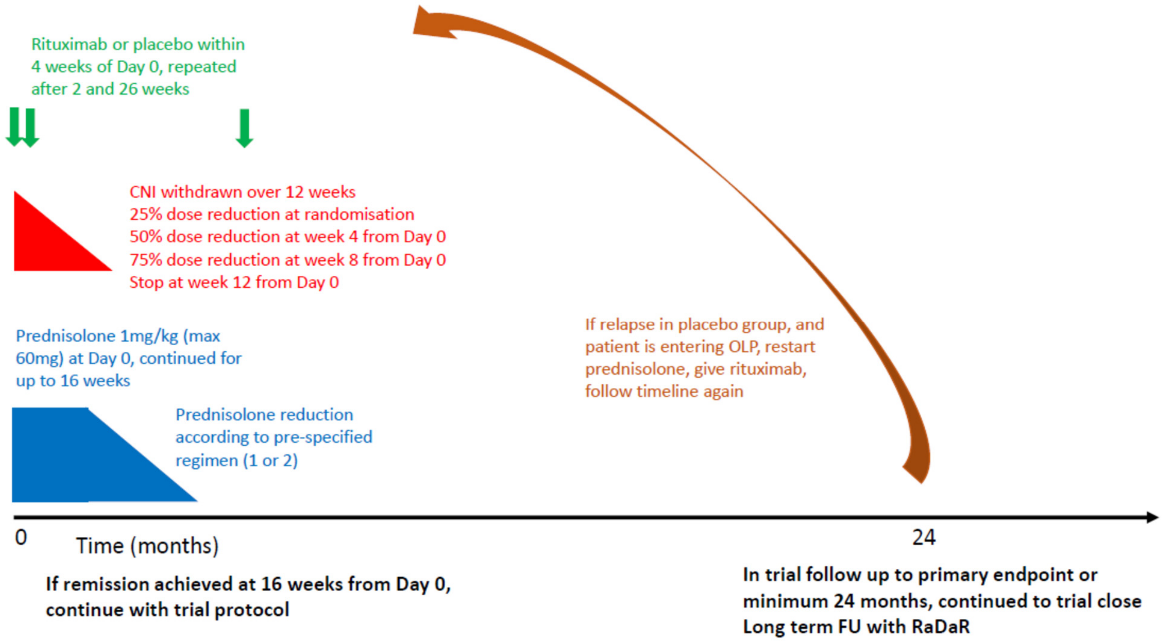

10.3.3 Concomitant therapy

Participants will continue to be treated with routine concomitant medications for other co-morbidities as appropriate within their current clinical condition, with modifications e.g. oral to IV switches, or temporary cessation as necessary. Appropriate supportive care may also be prescribed, including fluids, nutritional supplements and other medications as necessary. Other immune suppression for NS (excluding prednisolone and CNIs), e.g. mycophenolate mofetil will be stopped at randomisation.

All concomitant medications will be reviewed by local direct care team as per preferred local practice but only specified concomitant medications detailed below will be recorded in the CRF.

Prophylaxis against *Pneumocystis Jiroveci* pneumonia should be given for at least 6 months from starting trial treatment, as per local practice. It is recommended that blood pressure should be controlled to a target of < 130/80mm Hg. The use of Angiotensin Converting Enzyme inhibitors (ACEi) and Angiotensin Receptor Blockers (ARB) at the highest dose tolerated is strongly recommended and the use will be recorded.

Prophylaxis against glucocorticoid induced peptic ulceration and osteoporosis is also recommended as per local practice. Consideration should be given to performing a bone density assessment at the start of the trial and at six months to assess glucocorticoid induced bone loss.

Prophylaxis against venous thromboembolic disease is recommended in patients with serum albumin below 20g/l as per local practice.

Prophylaxis against tuberculosis is also recommended in high risk patients according to local practice.

The use of live viral vaccines during IMP treatment or while remaining B-cell depleted is not advised; should any vaccines be required during the study, it is recommended these should be completed at least 4 weeks prior to commencing the next dose of IMP.

10.4 Emergency Unblinding

During the double-blind phase of the trial, in the event of a valid medical or safety reason (e.g. in the case of an SAR where it is necessary for the investigator or treating health care professional to know which treatment the participant is receiving before the participant can be treated), the responsibility to break the treatment code resides with the treating clinician or a delegated member of the trial team. The chief investigator and co-lead investigator may be approached for emergency unblinding.

The online randomisation system will also be used for emergency unblinding. Appropriately trained and delegated site staff will be given the necessary access rights and permissions to access this facility. The name, contact details of the unblinder and reason for unblinding will be recorded within the system. An email stating that an unblinding has taken place will be automatically sent to the coordination team for oversight purposes. Site pharmacists may carry our emergency unblinding if the web based randomisation system is not available.

10.5 Accountability and Dispensing

10.5.1 Pharmacy responsibilities

Sites will maintain a full accountability record documenting receipt, storage, dispensing, return and destructions as appropriate, as specified in the pharmacy manual.

There will no accountability records maintained for nIMPs listed in section 10.3.

11 PROCEDURES AND ASSESSMENTS

11.1 Participant Identification

The trial will recruit adult patients with NS due to primary MCD/FSGS. Patients will be identified by local nephrologists from histology meetings and internal database review within each Renal Unit. Potential participants will be approached by their direct clinical care team in the first instance.

The trial will be publicised on the trial-specific website managed by the central co-ordination team at Cambridge Clinical Trials Unit, and via Patient View. Patient View gives patients secure access to their healthcare records including blood and urine results and diagnoses. Patients can also see trials for which they may be eligible - TURING will be visible to all patients who use this service and have been diagnosed with FSGS/MCD.

11.2 **Consent**

The Informed Consent form (ICF) must be approved by the REC and must be in compliance with GCP, local regulatory requirements and legal requirements. The investigator or designee must ensure that each trial participant is fully informed about the nature and objectives of the trial and possible risks associated with their participation.

The investigator or designee will obtain written informed consent from each participant before any trial-specific activity is performed. The ICF used for this trial and any relevant change made during the course of this trial, must be prospectively approved by the Research Ethics Committee (REC). The investigator at each site will retain the original of each participant signed ICF.

Should a participant require a verbal translation of the trial documentation by a locally approved interpreter/translator, it is the responsibility of the individual investigator to use locally approved translators.

Any new information which becomes available, which might affect the participant's willingness to continue participating in the trial will be communicated to the participant as soon as possible.

11.2.1 Recruitment to RaDaR and other research studies including NephroS

At screening, all participants will be enrolled in RaDaR. RaDaR is a registry study that collects laboratory data from patients with kidney disease. Data from patients enrolled in the RaDaR trial will be uploaded to the TURING trial database at the end of the TURING trial to improve completeness of the dataset. It will allow on-going data collection to be performed remotely after participants have completed the active phase of the trial, particularly with regard to long term effect on kidney function and progression to ESKD.

RaDaR data will be requested by the central team and patient identifiers will be required initially to link the RaDaR database with the TURING database. This identifiable information will be stored in the secure data hosting server hosted by the University of Cambridge and will only be accessed to ensure the correct participant is being linked with the correct record. Local trial teams will initially collect the identifiers on the eCRF but this information will not be visible to them after they have completed the required tabs in the e-CRF.

If available at the participating centre, participants will also be offered enrolment in PatientView. This not only allows patients to view their own results, but also allows the automatic capture and collation of results via RaDaR.

Participants will also be offered the opportunity to take part in other observational trials such as National Study of Nephrotic Syndrome (NephroS, REC number 09/H0106/90). This UK wide registry is an observational study of steroid-resistant and sensitive MCD/FSGS which

aims to provide a comprehensive genotype/phenotype correlation of MCD/FSGS. Recruiting trial participants into NephroS will allow blood and urine samples to be banked for future potential biomarker studies.

11.3 Screening Evaluation

11.3.1 Screening and Day 0

Screening and Day 0 (SPPR) visits may be performed separately, within a period of four weeks, or in a single combined visit wherever possible. Patients will start treatment with the pre-specified prednisolone regime (see Section 10.3) at the Day 0 (SPPR) visit, which may occur simultaneously with the screening visit if the treating clinician considers this clinically appropriate according to usual care. This may be before all data are available to determine eligibility.

Patients who have started Standard of Care treatment with prednisolone prior to consent may be enrolled in the trial if:

- the prednisolone was started within 4 weeks,
- the dosing used is consistent with the SPPR (section 10.3.1),
- patients are randomised and receive IMP within 4 weeks of starting SPPR.

In this situation, Day 0 would be when they started treatment with prednisolone for new onset/relapse of nephrotic syndrome. For these patients, serum albumin and urinary PCR tests will have been performed within the 7 days preceding SPPR (i.e. Day 0 -7). Patients may have been receiving more than 60mg prednisolone daily prior to randomisation. These patients are still eligible for the trial (if they have been receiving this dose for less than 4 weeks). In this group of patients, the prednisolone will be reduced to 60mg within 48 hours of randomisation.

Eligible participants will subsequently be randomised by a suitably trained and delegated member of the research team as described in Section 9.3 and the trial manual.

Trial visits, including screening and Day 0 assessments will be performed at the outpatient clinics.

Trial specific assessments will only be conducted after participants have given written informed consent. Where possible, eligibility blood and urine results will be obtained from standard of care visits.

Participants will be provided a container to collect 24 hour urine sample which they may bring back to the clinic during their next visit (< 4 weeks from Day 0). It is recommended that participants collect all urine produced starting from second urine of any day up until first urine of the following day.

Screening and Day 0 (SPPR) visits (which may be combined or occur over a period of up to 4 weeks) will include the following assessments:

- Demographics information
- Relevant medical history
- Height and weight

- Blood pressure
- Haematology
- Biochemical analysis
- Serum pregnancy test (if appropriate for female participants)
- Immunoglobulins G and M
- Urinary PCR
- Serology for hepatitis B, C and HIV
- Health status questionnaire: EQ-5D-5L
- Additional Health Costs questionnaire: out of pocket expenditure related to kidney disease over the previous 3 months, employment status, work productivity and activity impairment questionnaire, carer time
- A 24 hour urine collection for proteinuria
- Concomitant medications – see section 10.3.3
- Adverse event review
- Prednisolone dosing
- CNI dosing (if applicable)

Subsequent visit timings are based on the date of Day 0 (SPPR) i.e. when the standard prednisolone regime is started for treatment of the new onset/relapse of nephrotic syndrome, except for the post-remission assessments.

11.4 First and second dose of IMP

Within 4 weeks of Day 0 (SPPR), participants are expected to have the first dose of treatment administered. This may be a research visit at which only the IMP administration will take place, or may be combined with a trial visit if appropriate.

The second dose of IMP should be scheduled for two weeks after the first IMP dose, however a window of -1 week or +2 weeks can be utilised.

If appropriate for female participants, serum or urinary pregnancy tests will be carried out ≤ 14 days before each IMP infusion and recorded in the CRF.

No additional procedures will be mandated at these two visits; however AEs and concomitant medications will be assessed as standard. These visits may also be combined with trial visits if appropriate.

11.5 Pre-remission trial visits – aligned with standard of care

The following tests will be carried out every 4 (+/- 1 week) weeks until remission is achieved or until Week 16, whichever is earlier:

- Weight
- Blood pressure
- Haematology
- Biochemical analysis
- Urinary PCR
- 24 hour urine collection for proteinuria –week 16 only
- Concomitant medications - see section 10.3.3
- AE assessments

- Health status questionnaire: EQ-5D-5L
- Additional Health Costs questionnaire – week 16 only
- Prednisolone dosing
- CNI dosing (if applicable) at weeks 4, 8, 12, and 16

These procedures may be performed at a single visit, or may take place over a period of up to two weeks.

11.5.1 Week 16 Visit

Patients who have not responded to treatment by achieving partial or complete remission at the week 16 visit (16 weeks from Day 0, +/- 1 week) will leave the trial and return to standard of care treatment. These patients will not receive the third dose of rituximab or placebo at week 26 as they have not demonstrated steroid responsiveness, and on-going treatment with prednisolone with or without rituximab is likely to be futile.

11.6 **Remission Visit**

The remission trial visit will:

a. Be scheduled within two weeks of nephrotic symptoms resolving and urinalysis showing less +++ proteinuria on home or hospital testing
b. Be taken to have occurred if blood and urine tests at a trial visit confirm remission

At the remission visit (or over a period of up to 2 weeks from resolution of nephrotic syndrome), procedures performed will be:

- Weight
- Blood pressure
- Haematology
- Biochemical analysis
- Urinary PCR
- 24 hour urine collection for proteinuria (within two weeks of remission visit)
- Concomitant medications - see section 10.3.3
- AE assessments
- Issue patients with urine dipsticks for monitoring
- Health status questionnaire: EQ-5D-5L
- Additional Health Costs questionnaire: out of pocket expenditure related to kidney, employment status, work productivity and activity impairment questionnaire, carer time
- Prednisolone dosing
- CNI dosing (if applicable)
- Immunoglobulins G and M

11.7 **Post-remission trial visits - aligned with standard of care**

Following remission, trial visits will be performed every 4 weeks (+/- 1 week) for 12 weeks, then every 3 months (+/- 2 weeks) till trial end or relapse. Procedures will be performed (over a period of up to 2 weeks) as per the remission visit:

- Weight
- Blood pressure

- Haematology
- Biochemical analysis
- Urinary PCR
- 24 hour urine collection for proteinuria (performed at Month 24 only)
- Concomitant medications - see section 10.3.3
- AE assessments
- Urine protein stick testing compliance check – only once participants have achieved complete remission
- Health status questionnaire: EQ-5D-5L
- Additional Health Costs questionnaire (performed at 12 weeks post remission & then 6 monthly)
- Prednisolone dosing
- Immunoglobulins G and M (performed every 6 months post remission)

11.8 Week 24 (pre 3rd IMP dose) assessment

For the trial patients who achieved remission within 12 weeks from SPPR an extra trial visit will be mandated within 2 weeks preceding the 26 week treatment visit (or combined with the 26 week 3rd dose treatment visit). No extra visit will be required for patients who achieved remission after 12 weeks from SPPR if a trial visit is already scheduled in the two weeks preceding IMP administration, and the procedures for both visits will be combined. These patients will be attending the standard post-remission visits schedule as detailed above.

At the trial visit performed in the 2 weeks preceding the third IMP dose administration (or combined with the 26 week 3rd dose treatment visit), both questionnaires will be completed and 24 hour urine collection containers will be handed out. Female participants must have serum or urine pregnancy test performed and continued eligibility confirmed before the third dose of IMP is given.

11.9 Third dose of treatment

Patients in remission will receive the third dose of IMP at 26 weeks (+/- 2 weeks). No additional trial assessments will be mandated at this visit as patients will have had a trial visit in the preceding two weeks (unless it is combined with Week 24 (pre 3rd IMP dose) assessment as above)

11.10 24 months assessment

This trial specific visit will occur 24 months (+/- 2 weeks) after Day 0 (or day 0 in the OLP). The following procedures will be performed as part of this visit (over a period of up to 2 weeks):

- Weight
- Blood pressure
- Haematology
- Biochemical analysis
- Immunoglobulins G and M
- Urinary PCR
- 24 hour urine collection for proteinuria

- Concomitant medications - see section 10.3.3
- AE assessments
- Urine protein stick testing compliance check – only once participants have achieved complete remission
- Health status questionnaire: EQ-5D-5L
- Additional Health Costs questionnaire (performed at 12 weekly post remission & then 6 monthly)
- Prednisolone dosing

11.11 Relapse Visit

Patients will be asked to contact their local trial team for review if they have symptoms of relapse including increasing oedema and/or change in proteinuria on dipstick (patients in CR only). Relapse will be confirmed with blood and urine tests, in a trial visit within 2 weeks of indication of relapse. The assessments to be carried out at this visit will be identical to post-remission visit as well as a check for immunoglobulins G and M. However, no further urine dipstick testing will be required.

To help patients identify if they are relapsing, patients in CR will be shown how to test their urine with dipsticks. They will be asked to check their urine every week and to contact the trial team if "+++" or "++++" are visible on 2 consecutive days. This is purely an aid to help patients identify a relapse, and dipstick monitoring will not be recorded as part of the trial documentation. Patients in PR will not test their urine, as dipsticks are not able to detect relapse in this situation.

11.12 Final trial visit

The final trial visit will either be:

- At 16 weeks (+/- 1 week) from Day 0 (SPPR) for participants who have not entered complete or partial remission.
- At relapse (+ up to 2 week) for participants who have previously achieved complete or partial remission by week 16. Participants who have received placebo may then enter the Open Label Phase.
- Taken as the last 3 monthly visit they have had when the trial closes for participants who achieved complete or partial remission and did not relapse
- Please note: Trial participants withdrawn from treatment will require safety reporting (SAR/SUSAR only) for up to 12 months post last IMP dose providing that consent to participate in this trial is ongoing.

11.13 Open label phase visits (See table 11.15)

Participants who have achieved remission by week 16 and gone on to meet the primary endpoint of relapse will be unblinded by the local PI or a delegated member of the trial team (sub-investigator/associate PI) in a restricted fashion. This is to determine eligibility for the OLP. Consent for the OLP will be optional and will be sought from participants at entry to the main trial. Once participants have relapsed and become eligible for the OLP, a currently REC and or MHRA approved version of consent form must be signed by participant to confirm their

informed consent. The delegated clinician must confirm that there has been no change in clinical condition of the trial participants that could preclude rituximab treatment. A serum/urine pregnancy test must be performed before rituximab infusions can be given (if appropriate). The negative pregnancy test should be confirmed only at the local site and not communicated to central coordination team via CRF to preserve the blind.

Participants who are eligible to go into the OLP will be required to attend 3 research visits to receive rituximab infusions. AE and concomitant medication reviews will take place at standard of care clinic visits which will precede these infusion visits. Follow up visits and procedures will be performed at the same time points as during the RCT phase of the trial, with blood and urine results collected as per local clinical care guidelines and supplemented with data collected via RaDaR at the end of the trial. Protocolised prednisolone regimen has to be selected at OLP initiation and followed as per protocol. This regimen can be different to regimen previously tried in main trial. The date of restarting high dose steroids is taken as Day 0 in the OLP. Additional Health Costs questionnaire (out of pocket expenditure related to kidney, employment status, work productivity and activity impairment questionnaire, carer time) to assess impact of kidney disease on costs and productivity and EQ-5D-5L will also be completed as per the visit schedule for the RCT phase of the trial. As in the RCT phase, the third dose of rituximab at 26 weeks will not be administered if the patient does not achieve at least partial remission sustained to 26 weeks.

Participants in the OLP, who enter and remain in remission, will have 26 weeks of treatment (+/- 2 weeks) in the OLP with ongoing review 3 monthly until trial close. Participants will have the right to withdraw from the OLP at any time point without giving a reason for withdrawal.

After the trial has closed, the OLP data may also be analysed as a prospective cohort study.

11.14 All Trial Visits except IMP infusions:

At the discretion of the PI, any trial visit except IMP infusion visits may be done by telephone or NHS-approved videoconferencing facility instead of face to face. Remote assessments may be preferable, for instance, when the risk of SARS Cov2 infection is considered high.

Blood and urine tests may be collected at face to face visits, or at more local facilities, if possible. But, if the PI concludes that the risk of attending any healthcare setting is unacceptably high, blood and urine tests not critical for the primary endpoint may be omitted but should be done as soon as the risk level is reduced.
Trial participants will have to attend hospital or designated infusion unit to have IMP infusions (as per local policy).

Where trial visits and procedures take place over a number of days (up to a maximum of 14 days), the date that laboratory samples are collected shall be taken as the date of the trial visit.

11.15 Table of assessments for main phase of trial

Assessment	Screening / Eligibility check /Baseline			Pre remission phase[2]				REMISSION[3]	Post remission phase				Week 24 (+/- 2 weeks) (From Day 0[1]) (Pre 3rd Dose of IMP)	Month 24 (+/- 2 weeks) (From Day 0[1])	RELAPSE[3]
	Day 0[1]	Eligibility confirmation	Randomisation	Week 4 (+/- 1 wk)	Week 8 (+/- 1 wk)	Week 12 (+/- 1 wk)	Week 16[3 & 8] (+/- 1 wk)	≤2 weeks indication	REMISSION Week 4 (+/- 1wk)	REMISSION Week 8 (+/- 1wk)	REMISSION Week 12 (+/- 1wk)	REMISSION 3 monthly (+/- 2 wks)[4]			≤2 weeks indication
Informed consent		x													
Demographics		x													
Medical history		x													
Height		x													
Weight		x		x	x	x	x	x	x	x	x	x		x	x
Blood pressure		x		x	x	x	x	x	x	x	x	x		x	x
Haematology[5]		x		x	x	x	x	x	x	x	x	x		x	x
Biochemistry[6]	x			x	x	x	x	x	x	x	x	x		x	x
Pregnancy test[7]		x											x		
Immunoglobulins G & M		x						x				x[12]		x	x
Urinary PCR	x			x	x	x	x	x	x	x	x	x		x	x
24 hour urine collection for proteinuria		x					x	x					x	x	x
Serology for hepatitis B,C and HIV		x	x												

Concomitant medications check[11]		x		x	x	x	x	x	x	x	x	x		x	x
AEs assessments[11]		x		x	x	x	x	x	x	x	x	x		x	x
Optional urine protein stick testing (self-monitoring)[9]									x	x	x	x		x	
Health status questionnaire: EQ-5D-5L		x		x	x	x	x	x	x	x	x	x	x	x	x
Additional Health Costs questionnaire		x					x	x			x	x[10]	x	x	x
IMP administration				≤4 weeks after SPPR (dose 1), repeated 2 weeks later (- 1 week or + 2 weeks allowed)(dose 2), and in week 26 (+/- 2 weeks) (dose 3) if remission by week 16 achieved and maintained. *The week 24 visit could be combined with the week 26 IMP administration.*											
Prednisolone dosing/ compliance	x			x	x	x	x	x	x	x	x	x		x	x
CNI dosing (if taking at entry)				x	x	x	x								

1. *Day 0 is commencement of steroid treatment: eligibility blood and urine test results can follow. However randomisation must be after eligibility is confirmed. Day 0 (screening) and eligibility visits will be combined wherever possible, but must occur within a 4 week period if assessments performed over multiple visits.*
2. *Timings of treatment phase visits based on when first prednisolone dose given (SPPR). Once remission is achieved, move to post remission follow up visits*
3. *Please refer to section 11.12 of protocol for participant final visit timing*
4. *3 monthly post-remission visit starting 3 months after the week 12 post remission visit till trial close*
5. *Neutrophils, Lymphocytes.*
6. *Urea, creatinine, e-GFR and albumin.*
7. *Serum pregnancy test required for eligibility. The week 24 visit will be performed in the 2 weeks prior to administration of IMP, or the same day, and can be combined with a post remission trial visit if appropriate. Serum or urine pregnancy needs to be carried out ≤ 14 days before IMP administration in all women of child-bearing potential.*
8. *Participant should be withdrawn from the trial in case he/she hasn't achieved partial remission by week 16.*
9. *Optional dipstick testing encouraged for patients in complete remission only. See section 11.7 for further details.*
10. *Additional Health Cost Questionnaires performed 6 monthly in these visits.*
11. *AE assessments/ Con med check will be carried out in clinic visits before prescription for IMP infusion is given. They will not be checked on day of infusion unless combined with another trial visit. Trial participants withdrawn from treatment will require safety reporting (SAR/SUSAR only) for up to 12 months post last IMP dose.*
12. *Ig G & M done 6 monthly at these visits*

11.16 Table of assessments for Open Label Phase

Assessment	RELAPSE	Eligibility for OLP	Pre remission phase[1]				REMISSION	Post remission phase				Week 24 (+/- 2 weeks) (From Day of relapse[1]) (Pre 3rd Dose of IMP)	Month 24 (+/- 2wks) (From Day of relapse1)	RELAPSE (OLP- End of Trial)
	≤2 weeks indication		Week 4 (+/- 1 wk)	Week 8 (+/- 1 wk)	Week 12 (+/- 1 wk)	Week 16[5] (+/- 1wk)	≤2 weeks indication	REMISSION Week 4 (+/- 1 wk)	REMISSION Week 8 (+/- 1 wk)	REMISSION Week 12 (+/- 1wk)	REMISSION 3 monthly (+/- 2 wks)			≤2 weeks indication
Continued consent		x												
Weight	x		x	x	x	x	x	x	x	x	x		x	x
Blood pressure	x		x	x	x	x	x	x	x	x	x		x	x
Haematology[2]	x		x	x	x	x	x	x	x	x	x		x	x
Biochemistry[3]	x		x	x	x	x	x	x	x	x	x		x	x
Pregnancy test[4]		x[4]										x[4]		
Immunoglobulins G & M	x						x			x[9]	x		x	x
Urinary PCR	x		x	x	x	x	x	x	x	x	x		x	x
24 hour urine collection for proteinuria	x					x						x		x
Concomitant medications check[7]	x		x	x	x	x	x	x	x	x	x		x	x
AEs assessments[7]	x		x	x	x	x	x	x	x	x	x		x	x
Optional urine protein stick testing (self-monitoring)[6]								x	x	x	x		x	
Health status questionnaire: EQ-5D-5L	x		x	x	x	x	x	x	x	x	x	x	x	x
Additional Health Costs questionnaire	x					x	x			x	x[5]	x	x	x
Prednisolone Dosing/ Compliance	x[8]		x	x	x	x	x	x	x	x	x		x	x

IMP administration			≤4 weeks after RESTARTING TREATMENT DOSE PREDNISOLONE (dose 1), repeated 2 weeks later (- 1 week or + 2 weeks allowed)(dose 2), and after 26 weeks (+/- 2 weeks) (dose 3) if remission by week 16 achieved and maintained	

1. *Timings of pre-remission visits based on when treatment dose prednisolone is restarted for relapse. Once remission is achieved, move to post remission follow up visit*
2. *Neutrophils, Lymphocyte collected via RaDaR*
3. *Urea, creatinine, e-GFR and albumin collected via RaDaR*
4. *Serum pregnancy will be re-tested in female participants who are eligible for OLP before administration of first dose of rituximab. Urinary or serum pregnancy test will be done ≤14 days before third dose of rituximab. These should stay at site-level and should not be fed back to central team.*
5. *Additional Health Costs questionnaires performed 6 monthly in these visits.*
6. *Optional dipstick testing encouraged for patients in complete remission only. See section 11.7 for further detail.*
7. *AE assessments/ Con med check will be carried out in clinic visits before prescription for IMP infusion is given. They will not be checked on day of infusion unless combined with another trial visit.. Trial participants withdrawn from treatment will require safety reporting (SAR/SUSAR only) for up to 12 months post last IMP dose.*
8. *Choose regimen to follow. This can be different to one previously used in main trial. Reduction in dose once remission is achieved is as per protocol – as per section10.3.1*
9. *IgG and IgM checked 6 monthly post remission*

12 END OF TRIAL PARTICIPATION

All participants who enter remission will continue in trial follow up until relapse or trial closure when the total number of primary endpoint events have been observed (refer to section 16.2), whichever occurs first. It is anticipated that the end of trial will be approximately 30 months after the last patient recruited.

Once participants have completed their participation in the trial, they will return to normal standard of care.

12.1 Trial Restrictions

Women of childbearing potential are required to use adequate contraception for 12 months after the last dose of rituximab. This includes:

- Intrauterine Device (IUD).
- Hormonal based contraception (pill, contraceptive injection or implant etc.).
- Barrier contraception (condom and occlusive cap e.g. diaphragm or cervical cap with spermicide).
- True abstinence (where this is in accordance with the participant's preferred and usual lifestyle).

13 ASSESSMENT OF SAFETY

13.1 Definitions

13.1.1 Adverse event (AE)

Any untoward medical occurrence in a participant or clinical trial participant administered a medicinal product and which does not necessarily have a causal relationship with this treatment. An AE can therefore be any unfavorable and unintended sign (including an abnormal laboratory finding), symptom, or disease temporally associated with the use of an IMP, whether or not considered related to the IMP.

Please note: Recording of all SAEs and selected AEs of interest must start from the point of Informed Consent regardless of whether a participant has yet received a medicinal product.

13.1.2 Adverse Reaction (AR) to an IMP

All untoward and unintended responses to an IMP related to any dose administered. All AEs judged by either the reporting investigator or the sponsor as having a reasonable causal relationship to a medicinal product qualify as ARs. The expression reasonable causal relationship means to convey in general that there is evidence or argument to suggest a causal relationship.

13.1.3 Unexpected Adverse Reaction

An AR, the nature, or severity of which is not consistent with the applicable reference safety information (RSI) (e.g. the summary of product characteristics (SmPC) for an authorised product as specified in section 4.8. When the outcome of the AR is not consistent with the applicable RSI this AR should be considered as unexpected.

The term "severe" is often used to describe the intensity (severity) of a specific event. This is not the same as "serious," which is based on participant /event outcome or action criteria.

13.1.4 Serious Adverse Event or Serious Adverse Reaction (SAE / SAR)

Any untoward medical occurrence that at any dose:

- results in death
- is life-threatening
- requires hospitalisation or prolongation of existing inpatients´ hospitalization
- results in persistent or significant disability or incapacity
- is a congenital anomaly or birth defect
- is an important medical event - Some medical events may jeopardise the participant or may require an intervention to prevent one of the above characteristics/ consequences. Such events (hereinafter referred to as 'important medical events') should also be considered as 'serious'

Life-threatening in the definition of a SAE or SAR refers to an event in which the participant was at risk of death at the time of event; it does not refer to an event which hypothetically might have caused death if it were more severe.

13.1.5 Suspected Unexpected Serious Adverse Reaction (SUSAR)

A SAR, the nature and severity of which is not consistent with the information set out in the RSI.

13.1.6 Reference Safety Information (RSI)

A list of medical events that defines which reactions are expected for the IMP within a given trial and thus determining which SARs require expedited reporting.

The RSI is contained in a clearly identified section of the SmPC or the Investigator's Brochure (IB).

For this trial the RSI is: Section 4.8 (table 2) of the SmPC for Truxima, updated 09/08/2018 approved by the MHRA for use in this trial.

13.2 **Expected Adverse Reactions/Serious Adverse Reactions (AR /SARs)**

Detailed information about the established risks of rituximab, including expected adverse reactions (ARs), may be found in the latest MHRA approved version of the RSI as specified in section 13.1.6. Expectedness should relate to IMP and not underlying disease.

If the adverse reaction meets the criteria for seriousness, this must be reported as per section 13.5

The investigator or delegated members of the trial team will be responsible for detecting, documenting and reporting safety events that meet the definition of an AE/AR or SAE/SAR. Adverse Reactions of Special Interest related to trial treatment (Rituximab):

- Systemic infusion/injection reactions
- Hypogammaglobulinaemia (IgG <400 mg/dL (Grade 3) and <250 mg/dL (Grade 4))
- Hypersensitivity reactions

A number of other adverse reactions of special interest have been identified as being possibly related to the IMP (rituximab) or non-IMP (glucocorticoids or CNIs). – These will be monitored for the duration of the trial. These AERIs are as follows:

- Infections requiring antimicrobial, antiviral or antifungal treatment (including opportunistic, Progressive multifocal leukoencephalopathy)
- Malignancy

13.3 Expected Adverse Events/Serious Adverse Events (AE/SAE)

13.3.1 Reporting of expected adverse events

Due to the underlying clinical condition of the trial population it is not practicable to report all adverse events in this trial and it is thought that excessive safety reporting may detract from the main objectives of the trial. Rather, only AEs of special interest (AESI) should be reported as detailed in section 13.3.
Serious Adverse Events should be reported to the sponsor as detailed in section 13.5.

13.3.2 Laboratory and other safety assessment abnormalities reported as AEs and SAEs

Abnormal laboratory test results will not be recorded and/or reported as AEs unless they meet the criteria for SAE or AESI. Abnormal laboratory test results should be recorded in the medical notes.

13.3.3 Adverse events of special interest

A number of adverse events of special interest have been identified as being possibly related to trial treatment (rituximab) and non-IMP (glucocorticoids or CNIs). – These will be monitored for the duration of the trial. These AESIs are as follows:

Related to trial treatment (Rituximab):

- Systemic infusion/injection reactions
- Hypogammaglobulinaemia (IgG <400 mg/dL (Grade 3) and <250 mg/dL (Grade 4))
- Hypersensitivity reactions

Possibly related to the IMP (Rituximab) or non-IMP (glucocorticoids or CNIs):

- Infections requiring antimicrobial, antiviral or antifungal treatment (including opportunistic, Progressive multi-focal leukoencephalopathy)
- Malignancy

Possibly related to non-IMP (glucocorticoids or CNIs):

- New onset Diabetes Mellitus
- Osteoporosis/fracture
- Peptic ulceration/gastritis requiring medication/endoscopy
- Glaucoma
- Cataracts
- Weight gain > 10% (not attributed to oedema)
- Mood changes requiring medication

Hospital admissions – Only those related:

- D1. Hypovolaemia
- D2. Fluid overload secondary to nephrotic syndrome
- D3. Thromboembolic events (due to underlying disease)

Patients with nephrotic syndrome have frequent hospital admissions related to their underlying disease. Hospital admissions related to hypovolaemia or fluid overload secondary to nephrotic syndrome, or to thromboembolic events, will be **exempt from** expedited SAE reporting. However, these events will be designated as Adverse Events of Special Interest and details will be collected in the CRFs.

Each Principal Investigator must report all AESIs to the CI using the CRF in a timely manner. If the AESI is deemed to be serious, then the reporting procedure for an SAE should be followed as detailed in section 13.4, unless the AESI is related to hypovolaemia or fluid overload secondary to nephrotic syndrome.

13.4 Evaluation of AEs

The Sponsor expects that AEs are recorded in the medical notes from the point of Informed Consent regardless of whether a participant has yet received a medicinal product. Individual AEs should be evaluated by the investigator. This includes the evaluation of its seriousness, and any relationship between the IMP(s) and/or concomitant therapy and the AE (causality).

13.4.1 Assessment of seriousness

Seriousness is assessed against the criteria in section 13.1.4. This defines whether the event is an AE, SAE or SAR.

13.4.2 Assessment of causality

Definitely: A causal relationship is clinically/biologically certain. **This is therefore an Adverse Reaction**

Probable: A causal relationship is clinically / biologically highly plausible and there is a plausible time sequence between onset of the AE and administration of the IMP and there is a reasonable response on withdrawal. **This is therefore an Adverse Reaction.**

Possible: A causal relationship is clinically / biologically plausible and there is a plausible time sequence between onset of the AE and administration of the IMP. **This is therefore an Adverse Reaction.**

Unlikely: A causal relation is improbable and another documented cause of the AE is most plausible. **This is therefore an Adverse Event.**

Unrelated: A causal relationship can be definitely excluded and another documented cause of the AE is most plausible. **This is therefore an Adverse Event.**

Unlikely and Unrelated causalities are considered NOT to be trial drug related.

Definitely, Probable and Possible causalities are considered to be trial drug related.

A pre-existing condition must not be recorded as an AE or reported as an SAE unless the condition worsens during the trial and meets the criteria for reporting or recording in the appropriate section of the CRF.

13.4.3 *Clinical assessment of severity*

Mild: The participant is aware of the event or symptom, but the event or symptom is easily tolerated

Moderate: The participant experiences sufficient discomfort to interfere with or reduce his or her usual level of activity

Severe: Significant impairment of functioning; the subject is unable to carry out usual activities and / or the participant's life is at risk from the event.

13.4.4 *Recording of AEs*

AEs should be recorded in the medical notes. ARs and AESI should also be recorded in the appropriate section of the CRF. SAEs and SARs should be reported to the sponsor as detailed in section 13.4.

13.5 **Reporting SAEs/SARs**

Each PI needs to record all AEs in the medical notes and report all serious AEs to the CI and TURING trial team using the trial specific SAE form within 24 hours of knowledge of the event. However, SAEs related to the underlying disease, as indicated in Section 13.2.4, will be exempt from expedited SAE reporting. These events will be designated as Adverse Events of Special Interest and details will be collected in the CRFs.
The Chief Investigator (CI) is responsible for ensuring the assessment of all SAEs for expectedness and relatedness is completed and the onward notification of all SAEs to the Sponsor immediately but not more than 24 hours of site investigator awareness of the event. The sponsor has to keep detailed records of all SAEs reported to them by the trial team.

The CI is also responsible for prompt reporting of all SAEs findings to the competent authority (e.g. MHRA) of each concerned Member State if they could:

- adversely affect the health of participants,
- impact on the conduct of the trial,
- alter the risk to benefit ratio of the trial,
- alter the competent authority's authorisation to continue the trial in accordance with Directive 2001/20/EC.

The completed SAE form can be faxed or emailed. Details of where to report the SAE's can be found on the TURING SAE form and the front cover of the protocol.

13.6 **Reporting of SUSARs**

All suspected ARs related to an IMP (the tested IMP and comparators) which occur in the concerned trial, and that are both unexpected and serious (SUSARs) are subject to expedited reporting. Please see Section 13.1.6 for the RSI to be used in this trial.

13.6.1 *Who should report and whom to report to?*

The Sponsor delegates the responsibility of notification of SUSARs to the CI. The Chief Investigator must report all the relevant safety information previously described, to the:
- Sponsor

- Competent authorities in the concerned member states (e.g. MHRA)
- Ethics Committee in the concerned member states

The CI shall inform all investigators concerned of relevant information about SUSARs that could adversely affect the safety of participants.

13.6.2 When to report?

13.6.2.1 Fatal or life-threatening SUSARs

The CI must inform the Sponsor of any fatal SUSAR immediately but within 24 hours of the site investigator awareness of the event. The MHRA and Ethics Committee must be notified as soon as possible but no later than **7 calendar days** after the trial team and Sponsor has first knowledge of the minimum criteria for expedited reporting.

In each case relevant follow-up information should be sought and a report completed as soon as possible. It should be communicated to all parties within an additional **8 calendar days**.

13.6.2.2 Non-fatal and non-life-threatening SUSARs

All other SUSARs and safety issues must be reported to the Sponsor immediately but within 24 hours of the site investigator awareness of the event. The MHRA and Ethics Committee must be notified as soon as possible but no later than **15 calendar days** after first knowledge of the minimum criteria for expedited reporting. Further relevant follow-up information should be given as soon as possible.

13.6.3 Minimum criteria for initial expedited reporting of SUSARs

Information on the final description and evaluation of an adverse reaction report may not be available within the required time frames for reporting. For regulatory purposes, initial expedited reports should be submitted within the time limits as soon as the minimum following criteria are met:
a) a suspected investigational medicinal product
b) an identifiable participant (e.g. trial participant code number)
c) an adverse event assessed as serious and unexpected, and for which there is a reasonable suspected causal relationship
d) an identifiable reporting source

And, when available and applicable:
- a unique clinical trial identification (EudraCT number or in case of non-European Community trials the sponsor's trial protocol code number)
- a unique case identification (i.e. sponsor's case identification number)

13.6.3.1 Follow-up reports of SUSARs

In case of incomplete information at the time of initial reporting, all the appropriate information for an adequate analysis of causality should be actively sought from the reporter or other available sources. Further available relevant information should be reported as follow-up reports.

In certain cases, it may be appropriate to conduct follow-up of the long-term outcome of a particular reaction.

13.6.3.2 Format of the SUSAR reports

Electronic reporting is the expected method for expedited reporting of SUSARs to the competent authority. The format and content as defined by the competent authority should be adhered to.

13.7 Pregnancy reporting

All pregnancies within the trial should be reported to the CI/TURING Trial coordination center and the Sponsor using the relevant Pregnancy Reporting Form within 24 hours of notification. Pregnancies will be reported until trial close.

Pregnancy is not considered an AE unless a negative or consequential outcome is recorded for the mother or child/fetus. If the outcome meets the serious criteria, this would be considered an SAE.

14 EVALUATION OF RESULTS (Definitions and response/evaluation of outcome measures)

14.1 Definitions of Remission and Relapse

Primary outcome measure: Time from partial or complete remission (whichever documented first) to relapse as defined below (see appendix 6):

- COMPLETE REMISSION (CR): PCR<50mg/mmol (only one result required).

- PARTIAL REMISSION (PR): Reduction in Urinary PCR≥ 50% from Day 0 - Start of Protocolised Prednisolone Regimen (SPPR) AND PCR ≥50mg/mmol and ≤300mg/mmol on two consecutive results (performed within 14 days of each other). The date of first assessment is the date of partial remission.

- RELAPSE: recurrent NS as defined by albumin < 35 g/l, PCR >300mg/mmol and an increase in 100% over the nadir value during remission on two consecutive results (performed within 14 days of each other). The date of first assessment is the date of relapse. For patients with an albumin ≥35g/l prior to relapse, only one PCR result is required (i.e. PCR > 300mg/mmol that has risen by 100% over the nadir value and an albumin < 35g/L).

14.2 Evaluation Procedure

Remission will be assessed 4 (+/- 1) weekly from Day 0 (SPPR) until week 16. After remission, relapse will be assessed 4 (+/- 1) weekly for 12 weeks and then 3 (+/- 2 weeks) monthly from the date of remission. Additional assessments could be performed if clinically indicated or from the patient self-monitoring of urine protein stick testing results.

15 STORAGE AND ANALYSIS OF SAMPLES

No samples will be stored as part of TURING. All specimens will be analysed at each participating site. The exception to this will the urinalysis which will be performed by the

participant in their own homes and the results will be recorded in the urinalysis diary provided.

16 STATISTICS

16.1 Statistical Methods

16.1.1 Analysis populations

The following populations will be defined for efficacy and safety analyses:

- Intent-to-treat population (ITT)
  The ITT population is defined as all patients randomised in the trial, regardless of whether they actually received treatment. The treatment group will be analysed as randomised.
- Responding patient population
  Responding patient population is defined as all patients randomised in the trial, received allocated protocol treatment and achieved partial/complete remission. The treatment group will be analysed as randomised.
- Safety population
  The safety population comprises all patients randomised and having received at least one dose of trial treatment. The treatment group will be analysed as treated.

16.1.2 Primary assessment of the efficacy of rituximab at trial end

The primary objective of the trial is to assess the effect of rituximab on the time from remission to relapse of NS. The most clinically relevant benefit is to maintain remission as long as possible whilst minimising steroid toxicity, that is, the duration of remission in responding patients.

There is no evidence of difference in the distribution of time to remission between the two arms. The stratified log-rank test stratifying the factors used for randomisation will be used.

Otherwise, the probability-of-being-in-response function (PBRF)[51,52] will be used as a means of estimating the expected duration of remission (EDoR) across all randomised patients given as the estimated response rate times mean duration of remission for responding patients. The analysis will be based on ITT population.

Briefly, considering a stochastic process in which a patient must start in State 0 (that is at Day 0) and eventually progress to an absorbing state, 2 (progression, death in the absence of progression, treatment failure at week 16, relapse (or died from disease), possibly passing through a transient state 1 (remission). Response and duration of response are assumed to be independent.

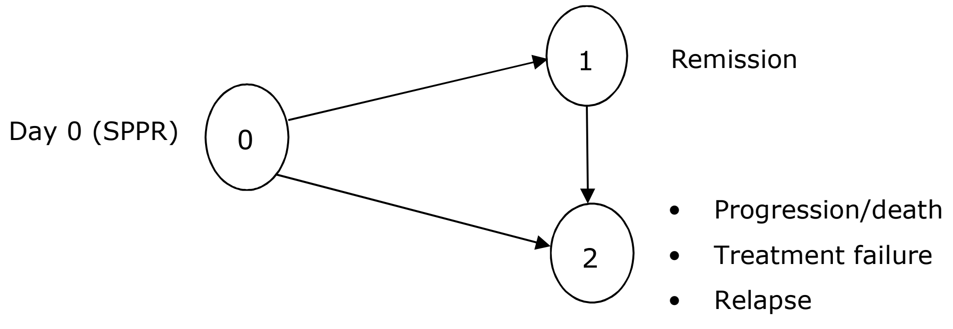

Summary statistics together with the Kaplan-Meier curves for the duration of remission in responding patients by treatment arm and estimated will also be presented. Detailed statistical analysis methods will be included in the statistical analysis plan.

16.1.3 *Censoring*

For patients who have achieved partial/complete remission but neither relapsed or died from the disease at the time of the analysis or who are lost to follow up, their data will be censored at the date that they were last known not to have relapsed.

16.1.4 *Safety analysis*

The safety analyses will be based on the safety population. All safety parameters will be summarised. Summary tables will be presented for incidence rates (number of patients with at least one incidence) of, AESIs, and SAEs.

16.1.5 *Statistical analysis plan*

A detailed statistical analysis plan will be finalised prior to any analyses being performed.

16.2 **Number of Patients to be Enrolled**

The primary outcome measure is time from remission to relapse from partial/complete remission. With 78 patients randomised at a steady rate over a period of 30 months, who subsequently achieved a partial/complete remission, and an additional 24 months follow-up after the last patient randomised (estimated at 54 months after the first patient was enrolled), the trial will have 80% power (two-sided test, significance level of 5%) to show a 50% change in time to relapse from a median value of 9 months in the control arm to 18 months in the research arm, (i.e. a hazard ratio of 0.50). It is expected that the 67 primary outcome measure events required will have occurred at this point. To achieve 85% power (two-sided test, significance level of 5%) requires 76 events. To achieve 90% power (two-sided test, significance level of 5%) requires 89 events. See appendix 5 for sample size output.

With an estimated 75% remission rate, allowing for non-compliance of the order of 5%, a total of approximately 112 (112*70%=78.4) would be needed. Further allowing for an early drop out (prior to remission assessment) of the order of 5% and withdrawal due to COVID-19 pandemic, approximately a minimum of 130 and up to 150 patients in total will be enrolled.

Although the effect size of HR=0.50 upon which sample size calculations are based appears large, two placebo RCTs of rituximab in children with FR NS have given lower HR at 0.267 (CI 0.135-0.528)[28] and 0.02 (CI 0.01-0.15)[29].

The assumptions used in the sample size estimation will be monitored by the Independent Data Monitoring Committee (IDMC). A sample size re-estimation might be performed if this is recommended by the trial IDMC.

16.3 **Interim Analyses**

We will conduct an interim feasibility assessment following 18 months of recruitment after enrolment of the first patient. The impact of a temporary pause in recruitment due to COVID-

19 will be considered. There are 5 potential outcomes at this stage:

1. More than 45 patients have been enrolled by month 18, the trial is feasible and will continue.
2. Between 36 and 45 patients have been recruited but site activation is slower than anticipated. Sites that have been opened are recruiting at an average rate of 2 patients per year. The trial will continue as recommended by the TSC and in discussion with the funding board.
3. Between 36 and 45 patients have been recruited. Sites that have been opened are recruiting at an average rate of less than 2 patients per year, but there are other mitigating factors that can be addressed to improve recruitment rate. Consideration will also be given to including more UK centres. These measures will be reviewed by the TSC and in discussion with the funding board to determine whether the trial is feasible.
4. Fewer than 36 patients have been recruited, with site activation slower than anticipated. Sites that have been opened are recruiting at an average rate of 2 patients per year. The trial team will seek to mitigate delays in site activation. Consideration will also be given to including more UK centres. These measures will be reviewed by the TSC and in discussion with the funding board to determine whether the trial is feasible.
5. Fewer than 36 patients have been recruited. Sites that have been opened are recruiting at an average rate of less than 2 patients per year. The trial will be considered infeasible unless there are other mitigating factors which the TSC and the NIHR board agree can be addressed.

16.4 Economic Evaluation

Cost-effectiveness, cost-utility and value of information analyses will be conducted from the perspectives of NHS and society over the trial duration (within-trial analysis), and over a patient lifetime (decision model-based analyses) comparing rituximab versus placebo.

Cost categories will be drugs (sourced from 'concomitant medications' measurement), secondary care contacts (sourced from HES), out of pocket costs and lost productivity accrued by the patient plus carer where applicable (patient questionnaires). The NHS perspective is defined as drugs and secondary care contacts. The societal perspective is defined as NHS plus out of pocket costs and lost productivity.

Outcome for the cost-effectiveness analysis will be adapted from the primary outcome (reported as disease and adverse event free period), and for the cost-utility analysis will be QALYs, using utilities calculated from the EQ5D5L using the recommended valuation set at the time of analysis, and integrated with respect to time.

A decision model will combine evidence from the clinical trial with other relevant evidence obtained from the literature to predict longer term costs and outcomes and hence cost-effectiveness. The model will be used to perform a value of information analysis which will predict the expected return on investment from further research into rituximab in MCD/FSGS.

16.5 Definition of the End of the Trial

The end of trial will be 30 months after the last patient has been recruited or when a minimum of 76 primary outcome events (section 16.2) have been observed, whichever occurs later.

17 DATA HANDLING AND RECORD KEEPING

17.1 CRF

All data will be transferred into a Case Report Form (CRF) (electronic or paper) which will be link anonymised using Trial subject ID and participant date of birth. All trial data in the CRF must be extracted from and be consistent with the relevant source documents. The CRFs must be completed, dated and signed by the investigator or designee in a timely manner. It remains the responsibility of the investigator for the timing, completeness, legibility and accuracy of the CRF pages. The CRF will be accessible to trial coordinators, data managers, the investigators, Clinical Trial Monitors, Auditors and Inspectors as required.

Copies of completed CRF's should be returned in timely manner to the trial coordinating centre at Cambridge Clinical Trials Unit in a timely manner of the pages being completed.

The investigator will retain a copy of each completed CRF page at site. The investigator will also supply the trial coordination centre with any required, anonymised background information with Trial subject ID from the medical records as required.

The investigators must ensure that the CRFs and other trial related documentation is sent to the trial coordination centre containing no patient identifiable data.

If completed on paper, all CRF pages must be clear and legible. Any errors should be crossed with a single stroke so that the original entry can still be seen. Corrections should be inserted and the change dated and initialled by the investigator or designee. If it is not clear why the change has been made, an explanation should be written next to the change. Typing correction fluid must not be used.

The electronic CRF system provides an edit feature that records the identity of the person making the change and retains a record of the before and after values of the data field(s) in question. In addition, all eCRF changes require electronic review and signoff by the investigator associated with the visit

17.2 Source data

To enable peer review, monitoring, audit and/or inspection the principal investigators must agree to keep records of all participating participants (sufficient information to link records e.g., CRFs, hospital records and samples), all original signed ICFs and copies of the CRF pages.

Source data/documents will include, but are not limited to: Patient Medical Records, signed ICFs, On-line test results (especially if only held electronically), sample logs, patient diaries and questionnaires

17.3 Data protection and participant confidentiality

All investigators and trial site staff involved in this trial must comply with the requirements of the EU General Data Protection Regulation (GDPR), Data Protection Act 2018 and Trust Policy with regards to the collection, storage, processing, transfer and disclosure of personal information and will uphold the Act's core principles.

Electronic case report forms (eCRFs) will be used for recording linked -anonymised trial data. eCRFs will be completed in accordance with GCP and ISO 15197: 2013 Guidelines. The trial identification number and participant date of birth will be used on eCRF.

18 TRIAL COMMITTEES

18.1 Trial Management Group (TMG)

The TMG will meet on a weekly basis during initial set up, and then at least monthly face to face or by teleconference to oversee the running of the trial. TMG members will review SAEs which have occurred in the trial. If there are specific safety concerns these may be raised with the TSC and IDMC. TMG members will include all TURING trial co-investigators, trial statistician, trial pharmacist, the trial co-ordinator and data manager at the Cambridge Clinical Trials Unit (CCTU).

18.2 Independent Trial Steering Committee (TSC)

Overall supervision of the trial will rest with the TSC. The TSC will be constituted in accordance with NIHR guidance. In particular, 66% of the TSC members will be required in order to achieve quorum; 75% of members voting must be independent; the TSC chair will be independent. A patient representative will serve as a member of the TSC. The TSC will meet every 6 months while participants receive the trial IMP and annually thereafter as fully detailed in the TSC Charter.

TSC will receive reports from the central co-ordination team at Cambridge CTU, TMG and IDMC.

18.3 Independent Data Monitoring Committee (IDMC)

The IDMC is independent of trial investigators and the central co-ordination team at Cambridge. The composition of the IDMC will be defined in the IDMC charter. The group will meet approximately every 6 months while patients are receiving trial treatment or until end of trial. The IDMC will review reports from the central co-ordination team at Cambridge and give advice on continuing recruitment. There are no formal stopping rules for efficacy.

During the trial, interim analyses of all serious adverse events (SAEs) will be provided regularly in strict confidence to the chairman of the independent DMC.

In light of these reports and any other information considered relevant, the IDMC will advise the TSC if, in their view, the randomised comparisons have provided both (i) "proof beyond

reasonable doubt" that for all, or some specific types of, patients rituximab is clearly indicated or clearly contraindicated; and (ii) evidence that might reasonably be expected to influence materially the patient management of many clinicians who are already aware of the results of other trials. The TSC can then decide whether the trial should be modified, or to seek additional data according to the IDMC recommendation. Unless this happens, the TSC, all investigators, trial participants and all trial staff (except those who provide the confidential analyses to the IDMC) will remain blind to any interim results until the end of the trial. If a decision is made to continue, the IDMC will advise on the frequency of future reviews of the data on the basis of accrual and event rates.

The IDMC will also monitor trial recruitment at 18 months after the enrolment of the first patient, the interim feasibility assessment.

19 ETHICAL AND REGULATORY CONSIDERATIONS

19.1 Consent

Please see section 11.2 for Informed Consent processes.

Consent to participate in other non-interventional trials including the National Study of Nephrotic Syndrome (NephroS) will be sought separately. Patients will be free to elect to participate in the main trial but to decline participation in other non-interventional trials such as the NephroS study.

19.2 Ethical Committee Review

Before the start of the trial or implementation of any relevant amendment, approval of the trial protocol, protocol amendments, ICFs and other relevant documents (e.g., advertisements and GP information letters if applicable) will be obtained from the REC. All relevant correspondence with the REC will be retained in the Trial Master File/Investigator Site File (TMF/ISF).

Annual reports will be submitted to the REC in accordance with national requirements. It is the CI's responsibility to produce the annual reports as required.

19.3 Regulatory compliance

The trial will not commence until a CTA is obtained from the MHRA. The protocol and trial conduct will comply with the Medicines for Human Use (Clinical Trials) Regulations 2004 and any relevant amendments.

Development Safety Update Reports (DSURs) will be submitted to the MHRA in accordance with national requirements. It is the CIs responsibility to produce the annual reports as required.

19.4 Protocol amendments

Protocol amendments must be reviewed and agreement received from the Sponsor for all

proposed amendments prior to submission to the Health Research Authority (HRA), REC and/or MHRA.

The only circumstance in which an amendment may be initiated prior to HRA, REC and/or MHRA approval is where the change is necessary to eliminate apparent, immediate risks to the participants (Urgent Safety Measures). In this case, accrual of new participants will be halted until the HRA, REC and/or MHRA approval has been obtained.

In the event of an Urgent Safety Measure, participating sites/investigators will be notified by email within 24 hours of awareness of the event necessitating the Urgent Safety Measures. The Sponsor, MHRA and REC will also be notified immediately but no more than 3 days following implementation.

19.5 Peer review

The research proposal this protocol has been based on has been peer reviewed by the National Institute for Health Research (NIHR) Health Technology Assessment (HTA) programme funding committee. A patient advisor has been involved in the design of this trial. Aspects of the trial have also been reviewed by patients with this disease, including the use of 24 hour urine collections and self-monitoring of urinalysis.

19.6 Declaration of Helsinki and Good Clinical Practice

The trial will be performed in accordance with the spirit and the letter of the declaration of Helsinki, the conditions and principles of GCP, the protocol and applicable local regulatory requirements and laws.

19.7 GCP training

All trial staff must hold evidence of appropriate GCP training or undergo GCP training prior to undertaking any responsibilities on this trial. This training should be updated every 2 years or in accordance with local Trust's policy.

20 SPONSORSHIP, FINANCIAL AND INSURANCE

The trial is sponsored by Cambridge University Hospitals NHS Foundation Trust. The trial is funded by NIHR HTA.

Cambridge University Hospitals NHS Foundation Trust, as a member of the NHS Clinical Negligence Scheme for Trusts, will accept full financial liability for harm caused to participants in the clinical trial caused through the negligence of its employees and honorary contract holders. There are no specific arrangements for compensation should a participant be harmed through participation in the trial, but no-one has acted negligently.

Participating sites will receive a capitation fee for each participant enrolled. Participants will not receive any expenses where trial visits align with standard of care visits, but will be eligible

for reimbursement of certain travel expenses incurred for visits for IMP/placebo. Participating centres will be paid via site specific trial agreements.

21 MONITORING, AUDIT AND INSPECTION

The investigator must make all trial documentation and related records available should an MHRA Inspection occur. Should a monitoring visit or audit be requested, the investigator must make the trial documentation and source data available to the Sponsor's representative. All participant data must be handled and treated confidentially.

The Sponsor's monitoring frequency will be determined by an initial risk assessment performed prior to the start of the trial. A detailed monitoring plan will be generated detailing the frequency and scope of the monitoring for the trial. Throughout the course of the trial, the risk assessment will be reviewed and the monitoring frequency adjusted as necessary.

Remote monitoring will be conducted for all participating sites. The scope and frequency of the monitoring will be determined by the risk assessment and detailed in the Monitoring Plan for the trial.

22 PROTOCOL COMPLIANCE AND BREACHES OF GCP

Prospective, planned deviations or waivers to the protocol are not allowed under the UK regulations on Clinical Trials and must not be used.

Participants will not be enrolled if they do not meet one or more eligibility criteria or restrictions specified in the trial protocol. In the event that eligibility criteria need to be changed /amended then they MUST first be approved by the relevant regulatory bodies via a protocol amendment before they can be implemented.

Protocol deviations, non-compliances, or breaches are departures from the approved protocol. They can happen at any time, but are not planned. They must be adequately documented on the relevant forms and reported to the CI and Sponsor immediately.

Deviations from the protocol which are found to occur constantly again and again will not be accepted and will require immediate action and could potentially be classified as a serious breach.

Any potential/suspected serious breaches of GCP must be reported immediately to the Sponsor without any delay.

23 PUBLICATIONS POLICY

Ownership of the data arising from this trial resides with the TMG. On completion of the trial the data will be analysed and tabulated and a Final Trial Report prepared. Draft copies of all trial manuscripts will be circulated to all collaborators for review prior to their submission for publication. Responsibility for all trial publications will rest with the TMG.

The main trial results will be presented at national and international conferences and published in a peer-reviewed journal, on behalf of all collaborators. All presentations and

publications related to the trial must be authorised by the TMG. The members of the TSC and IDMC will be listed with their affiliations in the Acknowledgements /Appendix of the main publication.

NIHR HTA, as the funding body, will be acknowledged within all publications.

24 REFERENCES

1. Rydel JJ, et al. Focal segmental glomerular sclerosis in adults: presentation, course, and response to treatment. Am J Kidney Dis. 1995; 25(4):534-42
2. Korbet SM, et al. Primary focal segmental glomerulosclerosis: clinical course and response to therapy. Am J Kidney Dis. 1994; 23(6):773-83.
3. Korbet SM. Angiotensin antagonists and steroids in the treatment of focal segmental glomerulosclerosis. Semin Nephrol. 2003; 23(2):219-28.
4. Stirling CM, et al. Treatment and outcome of adult patients with primary focal segmental glomerulosclerosis in five UK renal units. QJM. 2005; 98(6):443-9.
5. Hull RP, Goldsmith DJ. Nephrotic syndrome in adults. BMJ. 2008; 24, 336 (7654):1185-9.
6. Ponticelli C. Recurrence of focal segmental glomerular sclerosis (FSGS) after renal transplantation. Nephrol Dial Transplant. 2010; 25, 25–31.
7. Maas R, et al. Permeability factors in idiopathic nephrotic syndrome: historical perspectives and lessons for the future. Nephrol Dial Transplant. 2014; 29; 2207–2216.
8. Araya C, et al. T regulatory cell function in idiopathic minimal lesion nephrotic syndrome. - PubMed - NCBI. Pediatric Nephrology (Berlin, Germany). 2009; 24, 1691–1698.
9. Prasad N, et al. Differential alteration in peripheral T-regulatory and T-effector cells with change in P-glycoprotein expression in Childhood NS: A longitudinal study. Cytokine. 2015; 72, 190–196.
10. Le Berre L, et al. Induction of T Regulatory Cells Attenuates Idiopathic Nephrotic Syndrome. J Am Soc Nephrol. 2009; 20, 57–67.
11. Benz K, et al. Characterisation of renal immune cell infiltrates in children with nephrotic syndrome. Pediatr Nephrol. 2010; 25(7):1291-8.
12. Kim AH, et al. B cell-derived IL-4 acts on podocytes to induce proteinuria and foot process effacement. JCI Insight. 2017; 2, 2(21).
13. KDIGO Clinical Practice Guideline for the Treatment of Glomerulonephritis Chapter 5: Minimal-change disease in adults. Kidney International Supplements 2. 2012; 177–180.
14. KDIGO Clinical Practice Guideline for the Treatment of Glomerulonephritis Chapter 6: Idiopathic focal segmental glomerulosclerosis in adults. Kidney International Supplements 2. 2012; 181–185.
15. Floege, J. & Amann, K. Primary glomerulonephritides. The Lancet. 2016; 387, 2036–2048.
16. Thomas DB, et al Clinical and pathologic characteristic of focal segmental glomerulosclerosis variants Kidney International. 2006; 69(5):920.
17. Troyanov S, et al. Toronto Glomerulonephritis Registry Group. Focal and segmental glomerulosclerosis: definition and relevance of a partial remission. J Am Soc Nephrol. 2005; 16(4):1061-8.
18. Steenkamp, R et al. UK Renal Registry 15th annual report: Chapter 5 survival and causes of death of UK adult patients on renal replacement therapy in 2011: national and centre-specific analyses. Nephron Clin Pract. 2013; 123 (Suppl 1): 93–123.
19. Kerr M, et al. Estimating the financial cost of chronic kidney disease to the NHS in England.

Nephrol Dial Transplant. 2012; 27 (Suppl 3): iii73–iii80.
20. Ponticelli, C. et al. Cyclosporin versus cyclophosphamide for patients with steroid-dependent and frequently relapsing idiopathic nephrotic syndrome: a multicentre randomized controlled trial. Nephrology Dialysis Transplantation. 1993; 8, 1326–1332.
21. Li X, et al. Tacrolimus as a steroid-sparing agent for adults with steroid-dependent minimal change nephrotic syndrome. Nephrol Dial Transplant. 2008; 23, 1919–1925.
22. Li X, et al. Tacrolimus as rescue therapy for adult-onset refractory minimal change nephrotic syndrome with reversible acute renal failure. Nephrol Dial Transplant. 2013; 28, 2306–2312.
23. Choudhry, S, et al. Efficacy and Safety of Tacrolimus Versus Cyclosporine in Children With Steroid-Resistant Nephrotic Syndrome: A Randomized Controlled Trial. American Journal of Kidney Diseases. 2009; 53, 760–769.
24. Gipson DS, et al. Clinical trial of focal segmental glomerulosclerosis in children and young adults. Kidney international. 2011; 80, 868–878.
25. Van Vollenhoven RF, et al. Long-term safety of rituximab in rheumatoid arthritis: 9.5-year follow-up of the global clinical trial programme with a focus on adverse events of interest in RA patients. Ann Rheum Dis. 2013; 72, 1496–1502.
26. Glassock RJ. Therapy of relapsing minimal-change disease in adults: a new approach? Kidney international. 2013; 83, 343–345.
27. Fornoni A, et al. Rituximab Targets Podocytes in Recurrent Focal Segmental Glomerulosclerosis. Sci Transl Med. 2011; 3, 85ra46–85ra46.
28. Ravani P, et al. Short-term effects of rituximab in children with steroid- and calcineurin-dependent nephrotic syndrome: a randomized controlled trial. Clin J Am Soc Nephrol. 2011; 6, 1308–1315.
29. Iijima K, et al. Rituximab for childhood-onset, complicated, frequently relapsing nephrotic syndrome or steroid-dependent nephrotic syndrome: a multicentre, double-blind, randomised, placebo-controlled trial. The Lancet. 2014; 384, 1273–1281.
30. Ravani P, et al. Rituximab in Children with Steroid-Dependent Nephrotic Syndrome: A Multicenter, Open-Label, Noninferiority, Randomized Controlled Trial. J Am Soc Nephrol. 2015; 26 (9):2259-66.
31. Gulati A, et al. Efficacy and safety of treatment with rituximab for difficult steroid-resistant and -dependent nephrotic syndrome: multicentric report. Clin J Am Soc Nephrol. 2010; 5, 2207–2212.
32. Guigonis V, et al. Rituximab treatment for severe steroid- or cyclosporine-dependent nephrotic syndrome: a multicentric series of 22 cases. Pediatric nephrology (Berlin, Germany). 2008; 23, 1269–1279.
33. Kamei K, et al. Single dose of rituximab for refractory steroid-dependent nephrotic syndrome in children. Pediatric nephrology (Berlin, Germany). 2009; 24, 1321–1328.
34. Fujinaga S, et al. Single infusion of rituximab for persistent steroid-dependent minimal-change nephrotic syndrome after long-term cyclosporine. Pediatric nephrology (Berlin, Germany). 2010; 25, 539–544.
35. Kemper M, et al. Long-term follow-up after rituximab for steroid-dependent idiopathic nephrotic syndrome. Nephrology Dialysis Transplantation. 2012; 27, 1910–1915.
36. Munyentwali H, et al. Rituximab is an efficient and safe treatment in adults with steroid-dependent minimal change disease. Kidney international. 2013; 83, 511–516.
37. Guitard J, et al. Rituximab for minimal-change nephrotic syndrome in adulthood: predictive factors for response, long-term outcomes and tolerance. Nephrol Dial Transplant. 2014; 29, 2084–2091.
38. Takei T, et al. Effect of single-dose rituximab on steroid-dependent minimal-change nephrotic syndrome in adults. Nephrol Dial Transplant. 2013; 28, 1225–1232.

39. Iwabuchi Y, et al. Long-Term Prognosis of Adult Patients with Steroid-Dependent Minimal Change Nephrotic Syndrome Following Rituximab Treatment. Medicine (Baltimore). 2014; 93, e300.
40. Papakrivopoulou E, et al. Effective treatment with rituximab for the maintenance of remission in frequently relapsing minimal change disease. Nephrology (Carlton). 2016; 21, 893–900.
41. Fernandez-Fresnedo G, et al. Rituximab Treatment of Adult Patients with Steroid-Resistant Focal Segmental Glomerulosclerosis. Clinical Journal of the American Society of Nephrology. 2009; 4, 1317–1323.
42. Ruggenenti P, et al. Rituximab in steroid-dependent or frequently relapsing idiopathic nephrotic syndrome. J Am Soc Nephrol. 2014; 25, 850–863.
43. WHO Model List of Essential Medicines. Available at: http://www.who.int/medicines/publications/essentialmedicines/EML_2015_FINAL_amended_NOV2015.pdf?ua=1. (Accessed: 3rd January 2017)
44. Weidenbusch M, et al. Beyond the LUNAR trial. Efficacy of rituximab in refractory lupus nephritis. Nephrol Dial Transplant. 2013; 28, 106–111.
45. Condon M, et al. Prospective observational single-centre cohort study to evaluate the effectiveness of treating lupus nephritis with rituximab and mycophenolate mofetil but no oral steroids. Ann Rheum Dis. 2013; 72, 1280–1286.
46. Jones R, et al. Rituximab versus Cyclophosphamide in ANCA-Associated Renal Vasculitis. N Engl J Med. 2010; 363, 211–220.
47. Stone J, et al. Rituximab versus Cyclophosphamide for ANCA-Associated Vasculitis. N Engl J Med. 2010; 363, 221–232.
48. Smith R, et al. Rituximab for remission maintenance in relapsing antineutrophil cytoplasmic antibody–associated vasculitis. Arthritis Rheum. 2012; 64, 3760–3769.
49. Alberici F, et al. Long-term follow-up of patients who received repeat-dose rituximab as maintenance therapy for ANCA-associated vasculitis. Rheumatology (Oxford). 2015; 54 (7):1153-60.
50. Magnasco A, et al. Rituximab in children with resistant idiopathic nephrotic syndrome. J Am Soc Nephrol. 2012;23:1117-24
51. Temkin. An analysis for transient states with application to tumor shrinkage. Biometrics. 1978; 34: 571-580.
52. Begg and Larson. A study of the use of the probability-of-being-in-response functions as a summary of tumor response data. Biometrics. 1982; 38: 59-66.

25 APPENDICES

25.1 Appendix 1 - Trial management / responsibilities

25.1.1 Patient registration/ randomisation procedure

Following informed consent and confirmation of eligibility patients will be randomised into either rituximab or placebo group of the trial via a web- based randomisation system. This will be done by the CI/PI and suitably trained delegated site staff through a secure log-in. A paper-based randomisation system will also be in place as a back-up if the online randomisation system is unavailable. The CCTU coordinator(s), data manager, site research teams will receive notifications of randomisations.

25.1.2 CRF completion and data management

All CRFs should be completed in the manner and timelines detailed in the CRF completion guidelines and sent to the CCTU coordinator by or email. The local PI will be responsible for overseeing the collection of accurate data at each participating site. The completed CRFs will be signed by either the PI or a suitably qualified and delegated member of their trial team. The Data management team at the CCTU will be responsible for checking CRFs, entering data into a trial specific database and issuing queries according to the trial Data Management Plan. Queries will be sent to participating sites for resolution.

The electronic CRF system provides an audit trail feature that records the identity of the person making the change and retains a record of the before and after values of the data field(s) in question. In addition, all eCRF changes require electronic review and signoff by the investigator associated with the visit.

25.1.3 Preparation and submission of amendments

The trial coordinator(s) will be responsible for the preparation and submission of amendment documentation to the sponsor and appropriate authorities. Approvals of these amendments will be disseminated by the trial coordinator(s) to all participating sites for implementation.

25.1.4 Preparation and submission of annual safety report/annual progress reports

In collaboration with the CI, the trial coordinator(s) will prepare the annual progress report (APR), DSUR and funder reports, and submit these within the appropriate timelines.

25.1.5 Trial monitoring

The frequency, scope and method of monitoring will be determined by the Sponsor's trial level risk assessment and will be detailed in the trial monitoring plan. All participating sites will be monitored in accordance with the Sponsor's SOP.

25.1.6 Data protection/ confidentiality

All investigators and trial site staff involved in this trial must comply with the requirements of the Data Protection Act 2018 and Trust policy with regards to the collection, storage, processing and disclosure of personal information and will uphold the Act's core principles.

25.1.7 Trial documentation and archiving

All trial documentation will be retained in a secure location during the conduct of the trial and following end of trial, archived in accordance with the Sponsor's SOPs. Each participating site will be responsible for archiving the ISF and associated trial documentation.

25.2 Appendix 2 – Authorisation of participating sites

25.2.1 Required documentation

Prior to initiating a participating site, the following documentation is required:

- PI and other key trial team staff CV (signed and dated) and GCP certificate
- Competent Authority approval (HRA, REC, MHRA)
- Local R & D capability and capacity approval
- Participating Site Agreement executed, including pharmacy participating site agreement
- Patient Information Sheets and consent forms on local headed paper
- Protocol signed and dated by PI
- Delegation of Authority Log
- Confirmation of randomisation system training

25.2.2 Procedure for initiating/opening a new site

When all the regulatory paperwork is in place, prior to site opening, an initiation meeting will take place, either face-to-face or via a teleconference. This will be led by the clinical trial coordinator or delegate with as many of the local team present as is practicable. This initiation meeting constitutes training for the trial and it is therefore imperative that all members of the trial team who will be involved in the trial are represented at the meeting. A log of attendees will be completed during the meeting. The presentation slides will be provided to the site in advance of the meeting. A trial initiation form will be completed for each site initiation meeting. Copies of all initiation documentation must be retained in the ISF and TMF.

The sponsor's regulatory green light procedure will be followed.

25.2.2.1 Principal Investigator (PI) responsibilities

The PI has overall responsibility for the conduct of the trial at the participating site.
In particular, the PI has responsibilities which include (but are not limited to):

- Ensuring the appropriate approvals are sought and obtained
- Continuing oversight of the trial conduct at their site
- Ensuring the trial is conducted according to the protocol
- Ensuring consent is received in accordance with the protocol and national requirements
- Ensuring that the ISF is accurately maintained
- Delegation of activities to appropriately trained staff (this must be documented on the Delegation of Authority Log)
- Providing protocol or specialised training to new members of the trial team and ensuring that if tasks are delegated, the member of staff is appropriately trained and qualified
- Appropriate attendance at the initiation meeting
- Ensuring appropriate attendance at the TSC/IDMC meetings if required and ensuring appropriate safety information is made available to the coordinating centre team in advance of the meetings
- Dissemination of important safety or trial-related information to all stakeholders at the participating site

- Safety reporting within the timelines and assessment of causality and expectedness of all SAEs

25.3 Appendix 3 – Safety Reporting Flow Chart

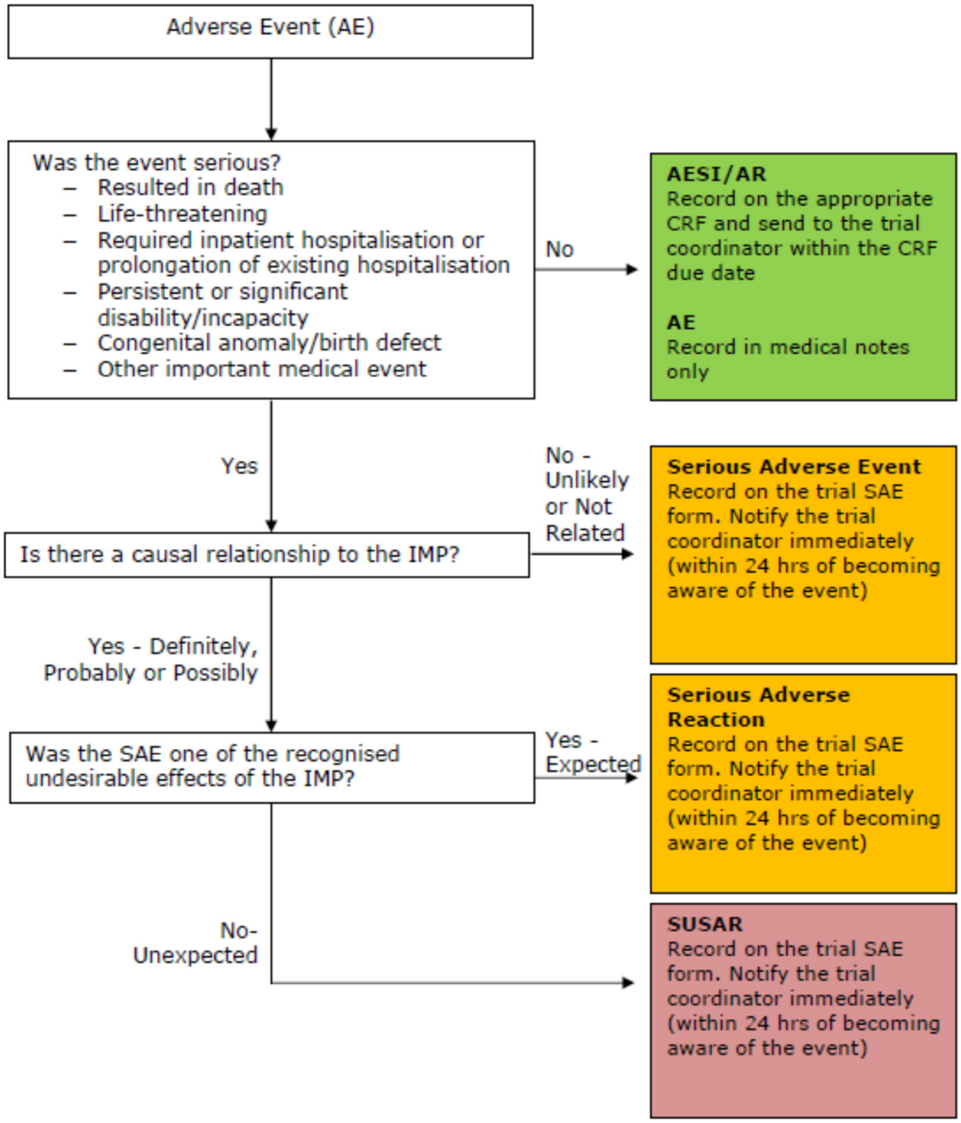

25.4 **Appendix 4 – EQ-5D-5L Questionnaire**

Under each heading, please tick the ONE box that best describes your health TODAY.

MOBILITY

I have no problems in walking about ❑

I have slight problems in walking about ❑

I have moderate problems in walking about ❑

I have severe problems in walking about ❑

I am unable to walk about ❑

SELF-CARE

I have no problems washing or dressing myself ❑

I have slight problems washing or dressing myself ❑

I have moderate problems washing or dressing myself ❑

I have severe problems washing or dressing myself ❑

I am unable to wash or dress myself ❑

USUAL ACTIVITIES *(e.g. work, study, housework, family or leisure activities)*

I have no problems doing my usual activities ❑

I have slight problems doing my usual activities ❑

I have moderate problems doing my usual activities ❑

I have severe problems doing my usual activities ❑

I am unable to do my usual activities ❑

PAIN / DISCOMFORT

I have no pain or discomfort ❑

I have slight pain or discomfort ❑

I have moderate pain or discomfort ❑

I have severe pain or discomfort ❑

I have extreme pain or discomfort ❑

ANXIETY / DEPRESSION

I am not anxious or depressed ❑

I am slightly anxious or depressed ❑

I am moderately anxious or depressed ❑

I am severely anxious or depressed ❑

I am extremely anxious or depressed ❑

The best health you can imagine

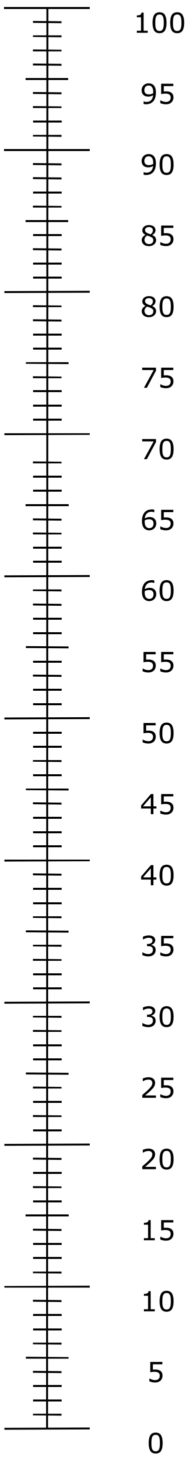

The worst health you can imagine

- We would like to know how good or bad your health is TODAY.
- This scale is numbered from 0 to 100.
- 100 means the best health you can imagine.
  0 means the worst health you can imagine.
- Mark an X on the scale to indicate how your health is TODAY.
- Now, please write the number you marked on the scale in the box below.

YOUR HEALTH TODAY =

25.5 Appendix 5 - Sample size output

. artsurv, method(l) nperiod(9) ngroups(2) fp(0) median(1.5) hratio(1, 0.5) alpha(0.05) power(0.8) aratios(1 1) recrt(5 0, 1, 0) distant(0) detail(0) onesided(0) ni(0) tunit(2) trend(0)

ART - ANALYSIS OF RESOURCES FOR TRIALS (version 1.1.0, 10 December 2013)

A sample size program by Abdel G Babiker, Patrick Royston & Friederike Barthel,
MRC Clinical Trials Unit at UCL, London WC2B 6NH, UK.

Type of trial Superiority - time-to-event outcome
Statistical test assumed Unweighted logrank test (local)
Number of groups 2
Allocation ratio Equal group sizes

Total number of periods 9
Length of each period 6 months

Baseline median survival time 1.5 six-month periods
Survival probs per period (group 1) 0.630 0.397 0.250 0.157 0.099 0.063
0.039 0.025 0.016
Survival probs per period (group 2) 0.794 0.630 0.500 0.397 0.315 0.250
0.198 0.157 0.125
Number of recruitment periods 5
Number of follow-up periods 4
Method of accrual Uniform
Recruitment period-weights 1 1 1 1 1 0 0 0 0

Hazard ratios as entered (groups 1,2) 1, 0.5
Alpha 0.050 (two-sided)
Power (designed) 0.800

Total sample size (calculated) 78
Expected total number of events 67

. artsurv, method(l) nperiod(9) ngroups(2) fp(0) median(1.5) hratio(1, 0.5) alpha(0.05)

power(0.85) aratios(1 1) recrt(5 0, 1, 0) distant(0) detail(0) onesided(0) ni(0) tunit(2) trend(0)

ART - ANALYSIS OF RESOURCES FOR TRIALS (version 1.1.0, 10 December 2013)

A sample size program by Abdel G Babiker, Patrick Royston & Friederike Barthel,

MRC Clinical Trials Unit at UCL, London WC2B 6NH, UK.

Type of trial Superiority - time-to-event outcome

Statistical test assumed Unweighted logrank test (local)

Number of groups 2

Allocation ratio Equal group sizes

Total number of periods 9

Length of each period 6 months

Baseline median survival time 1.5 six-month periods

Survival probs per period (group 1) 0.630 0.397 0.250 0.157 0.099 0.063

0.039 0.025 0.016

Survival probs per period (group 2) 0.794 0.630 0.500 0.397 0.315 0.250

0.198 0.157 0.125

Number of recruitment periods 5

Number of follow-up periods 4

Method of accrual Uniform

Recruitment period-weights 1 1 1 1 1 0 0 0 0

Hazard ratios as entered (groups 1,2) 1, 0.5

Alpha 0.050 (two-sided)

Power (designed) 0.850

Total sample size (calculated) 89

Expected total number of events 76

. artsurv, method(l) nperiod(9) ngroups(2) fp(0) median(1.5) hratio(1, 0.5) alpha(0.05) power(0.90) aratios(1 1) recrt(5 0, 1, 0) distant(0) detail(0) onesided(0) ni(0) tunit(2) trend(0)

ART - ANALYSIS OF RESOURCES FOR TRIALS (version 1.1.0, 10 December 2013)

A sample size program by Abdel G Babiker, Patrick Royston & Friederike Barthel,
MRC Clinical Trials Unit at UCL, London WC2B 6NH, UK.

Type of trial Superiority - time-to-event outcome
Statistical test assumed Unweighted logrank test (local)
Number of groups 2
Allocation ratio Equal group sizes

Total number of periods 9
Length of each period 6 months

Baseline median survival time 1.5 six-month periods
Survival probs per period (group 1) 0.630 0.397 0.250 0.157 0.099 0.063
0.039 0.025 0.016
Survival probs per period (group 2) 0.794 0.630 0.500 0.397 0.315 0.250
0.198 0.157 0.125
Number of recruitment periods 5
Number of follow-up periods 4
Method of accrual Uniform
Recruitment period-weights 1 1 1 1 1 0 0 0 0

Hazard ratios as entered (groups 1,2) 1, 0.5
Alpha 0.050 (two-sided)
Power (designed) 0.900

Total sample size (calculated) 105
Expected total number of events 89

25.6 **Appendix 6 – Primary Outcome Measure Events**

<table>
<tr><td></td><td colspan="4">Pre Achieving Complete/Partial Remission</td><td colspan="5">Post Achieving Complete/Partial Remission</td></tr>
<tr><td>PCR level</td><td>PCR≤50mg/mmol</td><td colspan="3">50<PCR≤300 mg/mmol</td><td colspan="5">PCR>300 mg/mmol</td></tr>
<tr><td>PCR change from baseline or nadir</td><td></td><td colspan="2">PCR reduction from baseline ≥50%</td><td>PCR reduction from baseline <50%</td><td colspan="4">PCR increase from nadir value >100%</td><td>PCR increase from nadir value 100%</td></tr>
<tr><td>Albumin level</td><td>Any</td><td colspan="2">Any</td><td></td><td colspan="3">Albumin<35g/l</td><td>Albumin≥35g/l</td><td rowspan="2">Any</td></tr>
<tr><td rowspan="2">No. of PRC assessments required</td><td>One</td><td colspan="2">Two consecutive (within 14 days of each other)</td><td>Any</td><td colspan="3">Two consecutive (within 14 days of each other)</td><td>Any</td></tr>
<tr><td></td><td>Yes</td><td>No</td><td></td><td>Yes</td><td colspan="2">No</td><td rowspan="4"></td><td rowspan="4"></td></tr>
<tr><td rowspan="3">OUTCOME</td><td rowspan="3"><u>Complete Remission</u></td><td rowspan="3"><u>Partial Remission</u>
Date of first PCR is the date of Partial remission</td><td rowspan="3"></td><td rowspan="3"></td><td rowspan="3"><u>Relapse</u>
Date of first PCR is the date of relapse</td><td colspan="2">Albumin≥35g/l in the previous assessment</td></tr>
<tr><td>Yes</td><td>No</td></tr>
<tr><td><u>Relapse</u></td><td></td></tr>
</table>
